# Exploring the potential mechanisms of Danshen against COVID-19 via network pharmacology analysis and molecular docking

**DOI:** 10.1038/s41598-024-62363-x

**Published:** 2024-06-04

**Authors:** Qiang Zhang, Zongsuo Liang, Xiaoqing Wang, Siyu Zhang, Zongqi Yang

**Affiliations:** 1 College of Life Sciences, Key Laboratory of Plant Secondary Metabolism and Regulation of Zhejiang Province, Zhejiang Sci-Tech University, Hangzhou, 310018 China; 2 School of Art and Design, Zhejiang Sci-Tech University, Hangzhou, 310018 China; 3 Shaoxing Biomedical Research Institute of Zhejiang Sci-Tech University Co., Ltd, Zhejiang Engineering Research Center for the Development Technology of Medicinal and Edible Homologous Health Food, Shaoxing, 312075 China

**Keywords:** Network pharmacology, Danshen, COVID-19, Gene enrichment, Molecular dynamics simulations, Pharmaceutics, Biochemistry, Drug discovery

## Abstract

Danshen, a prominent herb in traditional Chinese medicine (TCM), is known for its potential to enhance physiological functions such as blood circulation, immune response, and resolve blood stasis. Despite the effectiveness of COVID-19 vaccination efforts, some individuals still face severe complications post-infection, including pulmonary fibrosis, myocarditis arrhythmias and stroke. This study employs a network pharmacology and molecular docking approach to investigate the potential mechanisms underlying the therapeutic effects of candidate components and targets from Danshen in the treatment of complications in COVID-19. Candidate components and targets from Danshen were extracted from the TCMSP Database, while COVID-19-related targets were obtained from Genecards. Venn diagram analysis identified common targets. A Protein–Protein interaction (PPI) network and gene enrichment analysis elucidated potential therapeutic mechanisms. Molecular docking evaluated interactions between core targets and candidate components, followed by molecular dynamics simulations to assess stability. We identified 59 potential candidate components and 123 targets in Danshen for COVID-19 treatment. PPI analysis revealed 12 core targets, and gene enrichment analysis highlighted modulated pathways. Molecular docking showed favorable interactions, with molecular dynamics simulations indicating high stability of key complexes. Receiver operating characteristic (ROC) curves validated the docking protocol. Our study unveils candidate compounds, core targets, and molecular mechanisms of Danshen in COVID-19 treatment. These findings provide a scientific foundation for further research and potential development of therapeutic drugs.

## Introduction

According to the World Health Organization (WHO), coronavirus disease-2019 (COVID-19) is an infectious disease caused by the severe acute respiratory syndrome coronavirus 2 (SARS-CoV-2). While vaccination efforts have demonstrated effectiveness in mitigating the risk of severe illness, hospitalization, and COVID-19-related fatalities to a certain extent, it is crucial to recognize that individuals previously infected with coronavirus 2 may still encounter notable long-term consequences, including lung damage, pulmonary fibrosis, myocarditis, arrhythmias, and stroke^1^. Therefore, there is an urgent need to develop effective approaches for the treatment of COVID-19-associated pneumonia. Traditional Chinese Medicine (TCM) has previously demonstrated unique advantages in the treatment of severe acute respiratory syndrome (SARS) in 2003 and H1N1 influenza in 2009^2,3^. Among the various medicinal plants used in TCM, Danshen holds a prominent position and has been applied for approximately two millennia since its first documentation in Shennong's herbal classic of materia medica^4,5^. The primary function of Danshen is to promote blood circulation^6^ and alleviate blood stasis^7,8^, making it effective in treating atherosclerosis^9,10^. Additionally, Danshen exhibits another numerous pharmacological activities, including anti-inflammatory^11,12^, antioxidant^13^, anticancer^14^, and antidiabetic effects^15^. Recent studies have suggested the therapeutic potential of Danshen in the context of COVID-19, with tanshinones derived from Danshen exhibiting inhibitory effects on SARS-CoV cysteine proteases^16^. Salvianolic acids have been found to hinder the entry of SARS-CoV-2 (2019-nCoV) spike pseudovirus into cells by binding to the receptor-binding domain of the 2019-nCoV spike protein and ACE2 protein^17^. Additionally, several other TCM herbs have demonstrated potential anti-infective properties in the treatment of COVID-19^18^.

**Figure 1:**
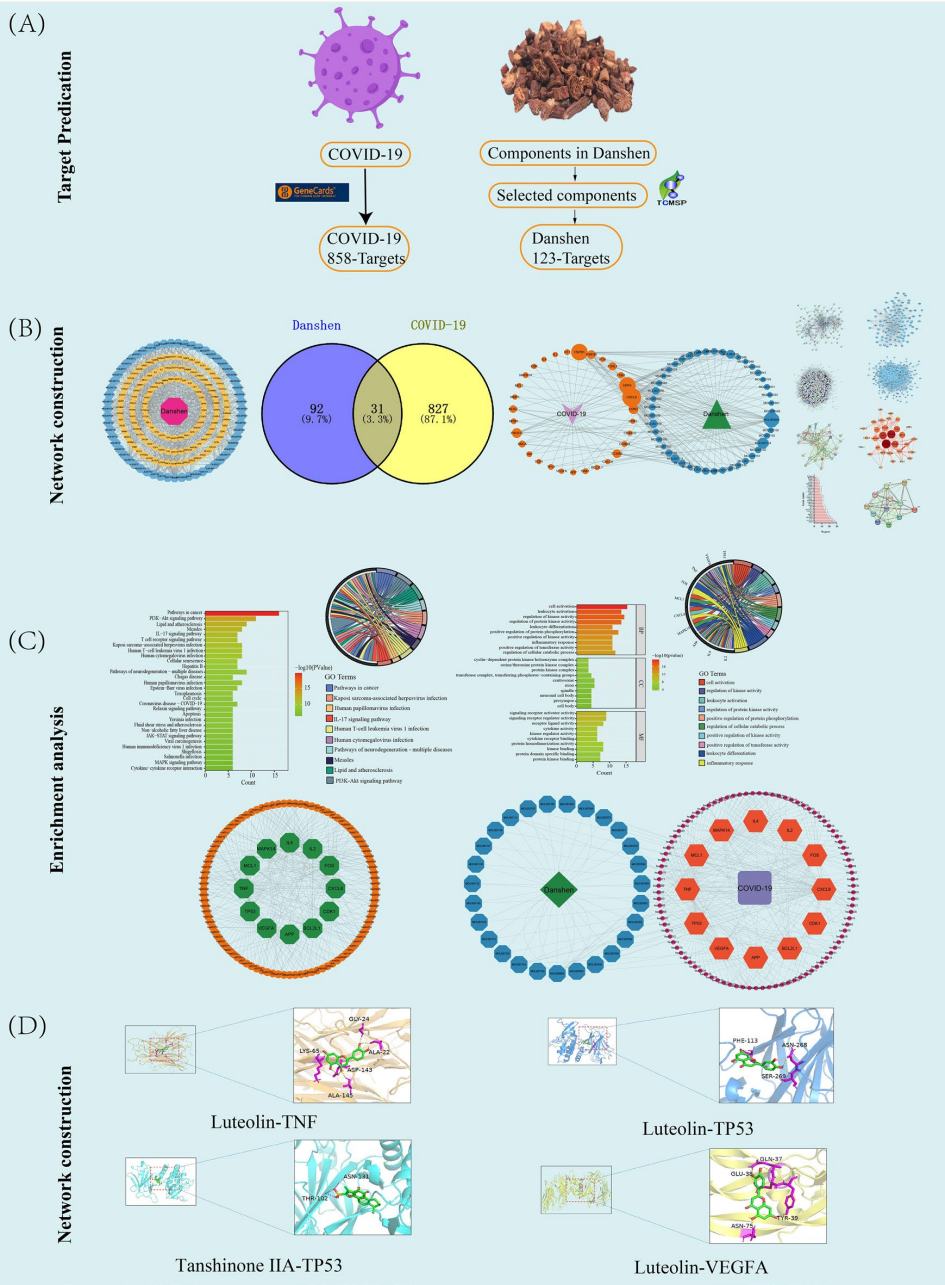
Workflow for Danshen treatment on COVID-19. (**A**) The first step was to predict targets. (**B**) The second step was to construct network. (**C**) The third step was to make enrichment analysis. (**D**) The fourth step was to develop molecular docking.

Network pharmacology, which explores the relationships among multiple components, targets, and pathways, has become a valuable tool in TCM research^19^. Therefore, in this study, we employed a network pharmacology approach to predict the potential therapeutic effects of Danshen in the treatment of COVID-19. Molecular docking and molecular dynamics were subsequently utilized to assess the binding situation between the candidate components from Danshen and core targets, providing a foundation for further investigation into the mechanisms underlying Danshen’s treatment of COVID-19. The workflow chart depicting the study design is presented in Fig. 1.

## Methods

### Identification of candidate components and targets of Danshen

The study employed the keyword "Danshen" to conduct a comprehensive search for pertinent information regarding the chemical constituents of Danshen within the traditional Chinese medicine systems pharmacology database and analysis platform (TCMSP)^20^. Subsequently, a meticulous screening process was implemented, employing strict inclusion criteria, wherein candidate components were required to possess an oral availability (OB) of ≥ 30% and a drug similarity (DL) of ≥ 0.18. The targets of the candidate components in Danshen were also predicted utilizing the TCMSP database. Following the elimination of duplicate entries, a comprehensive set of 123 targets was successfully identified. To ensure consistency and accuracy, the protein names associated with these targets were subsequently mapped to their official names using the UniProt database^21^.

### Prediction of disease targets

The keyword "novel coronavirus pneumonia" was used to search for COVID-19-related targets in the Genecards database^22^. Targets with a relevance score ≥ 2 were selected, resulting in the identification of 858 targets related to COVID-19.

### Construction of components–targets network

A network connecting the candidate components from Danshen and their related targets was established using Cytoscape software^23^. The targets of the candidate components and the COVID-19-related targets were imported into Venny online software to identify common targets. This leads to the development of a network representing the connections between Danshen components, common targets, and COVID-19.

### Construction of protein–protein interactions (PPI) network

To construct Protein–Protein interaction (PPI) networks, the targets of the candidate components from Danshen and the targets related to COVID-19 were inputted into the STRING database^24^. Separate PPI networks were built for each set of targets. Additionally, a PPI network was created using the common targets between the candidate components and COVID-19. The Cytoscape software was utilized for the construction and visualization of these PPI networks. The CytoNCA plugin, with "Degree" chosen as the scoring basis, was employed for further analysis and assessment within the PPI network.

### GO and KEGG pathway enrichment analysis

To elucidate the effects of the candidate components of Danshen on the disease, gene ontology (GO)^25^ and Kyoto encyclopedia of genes and genomes (KEGG)^26^ enrichment analyses were performed using the Metascape database^27^. The results were visualized using the Bioinformatics platform ((bioinformatics.com.cn)). The statistical significance threshold for the enrichment analysis was set at p ≤ 0.05.

### Components-targets molecular docking

The three-dimensional (3D) structure of the TP53 (PDB ID:6RZ3), TNF (PDB ID:1a8m), and VEGFA (PDB ID:4zff) were downloaded from the PDB database^28^. The structure of component luteolin and Tanshinone IIA were obtained from the TCMSP database in mol2 format. After that, molecules and interrelated proteins were imported into the AutoDockTools-1.5.^29^ software to complete the docking process. The docking parameter was set as a Genetic Algorithm for accuracy and comprehensiveness. Binding affinity was automatically calculated at the end of the process. Pymol software^30^ was then employed to visualize polar contacts between molecules and amino acid residues. TP53, TNF, and VEGFA were used as receptors, and the Luteolin and Tanshinone IIA were used as ligands. The ligand coordinate ascertained the candidate site of molecular docking in the target protein complex. The ligand was set to be flexible, and the receptor was rigid. For the docking results, a total of 4 conformations were created each.

### Molecular dynamics simulations

The molecular dynamics (MD) simulations for the TNFLuteolin and TP53-Luteolin systems were performed using Gromacs software package (2020)^31^. The protein and ligand were described by the ff14SB^32^ and GAFF^33^ force fields, respectively. Then, two systems were neutralized with an appropriate amount of counterion (Cl − or Na +), followed by solvation with TIP3P^34^ water molecules in an orthogonal box, leaving at least 10 Å between the solute atoms and the box boundary. Moreover, the particle mesh Ewald (PME)^35^ summation method was implemented to deal with Coulombic interactions, and the Shake^36^ algorithm was applied to restrain the bond vibrations involving hydrogen atoms. The MD simulations mainly included four steps: energy minimization, heating, equilibration, and production runs. Firstly, four minimizations were performed by utilizing the steepest descent and the conjugate gradient algorithm through 25,000 steps with the force constants of 5.0, 2.0, 0.1, and 0 kcal/mol/Å2 to all heavy atoms, protein backbone, and Cα atoms, respectively. Then, each system was heated from 0 to 310 K with a force constant of 5.0 kcal/mol/Å2 for all heavy atoms. Next, all systems were equilibrated through 3000 steps with harmonic restraints of 1.0, 0.5, and 0.1 kcal/mol/Å2 on all heavy atoms, protein backbone, and Cα atoms, respectively. Finally, a 100 ns MD simulations was simulated with randomized initial atomic velocities for each system.

### Binding free energy calculation and enrichment calculations

To evaluate the binding affinity of ligands on protein, the binding free energy was calculated using the MM/GBSA^37^ method for each system. Firstly, water molecules and counter ions were extracted from the MD trajectory, and then 250 snapshots at time intervals of 20 ps were taken from the last 20-ns MD simulation. For each snapshot, the binding free energy ("∆" "G" _"bind") was calculated utilizing the following formula:

$$\Delta {\text{G}}_{{{\text{bind}}}} { = }\Delta {\text{G}}_{{{\text{complex}}}} - \Delta {\text{G}}_{{{\text{receptor}}}} - \Delta {\text{G}}_{{{\text{ligand}}}} .$$

To assess the validity of the docking protocol, we conducted enrichment calculations using PyRx^38^ and SPSS Statistics^39^ software. Specifically, we utilized 500 decoy compounds from the DUD.E^40^ database and 10 validated compounds along with Luteolin. Autodocking was performed on these compounds, and subsequently, we generated receiver operating characteristic (ROC) curves based on the obtained docking scores. The ROC curves provide a visual representation of the trade-off between true-positive (TP) and false-positive (FP) results. To quantify the performance of the docking protocol, we computed the area under the curve (AUC) of the ROC curves. The AUC value ranges between 0 and 1, with a value close to 0.5 indicating random selection of TP and FP results, while a value close to 1 suggests a higher likelihood of correctly identifying TP results before encountering FP results. By analyzing the ROC curve and examining the computed AUC, we can gain valuable insights into the predictive capability and effectiveness of our docking protocol in distinguishing true positives from false positives^41^.

## Results

### Targets prediction and network construction

**Figure 2:**
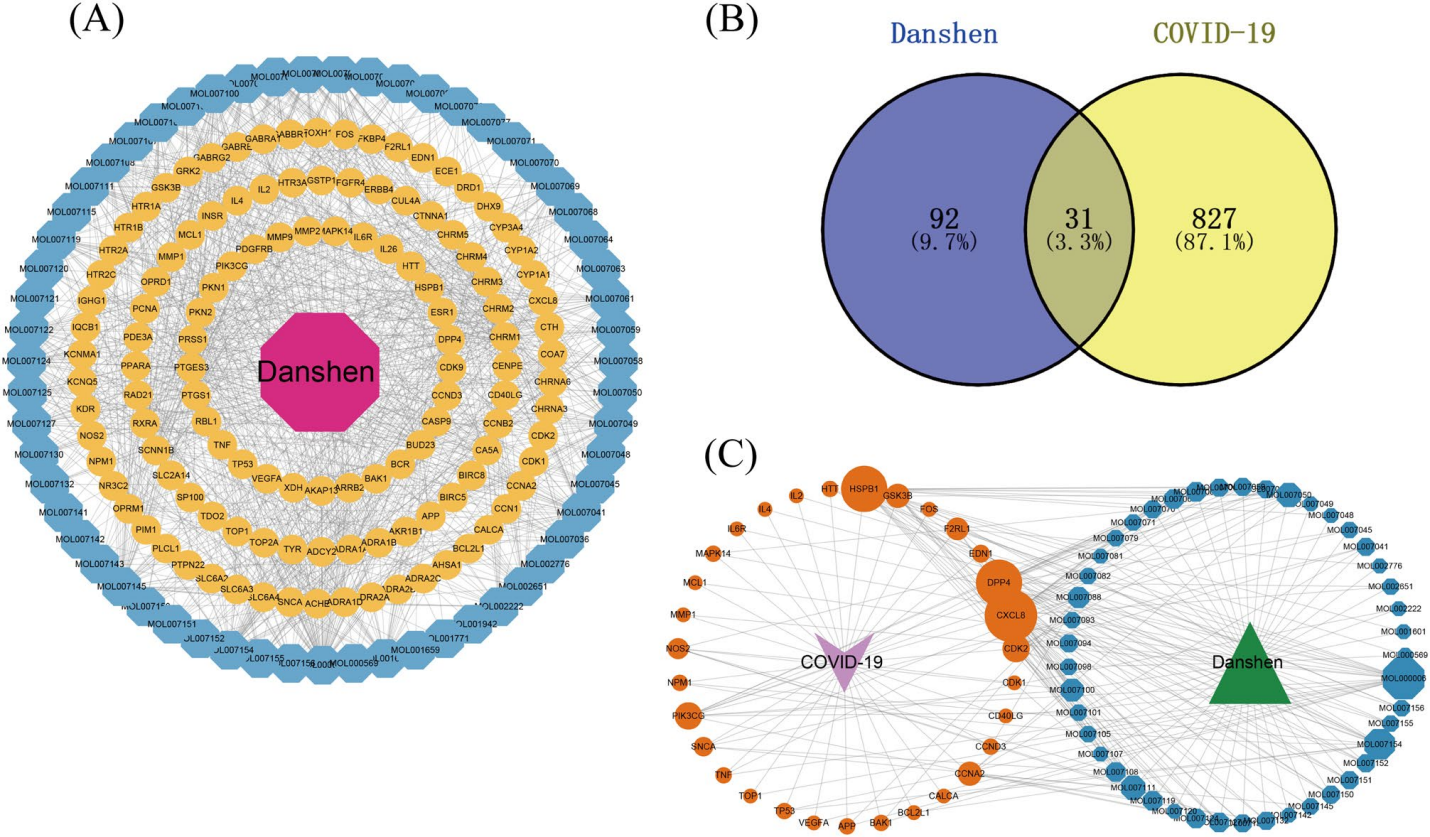
Targets prediction and network construction. (**A**) Danshen-components-targets network. The red octagon represents Danshen. Yellow Ellipse represents targets. The Blue octagon represents candidate components. (**B**) Venn diagram of 31 potential common targets. (**C**) Danshen-components-candidate targets-COVID-19 network. Yellow Ellipse represents common targets. Blue Ellipse represents candidate components. Purple V represents COVID-19. Green Triangle represents Danshen. Nodes from small to large represent the degree value from low to high.

In the TCMSP database, a total of 202 initial components of Danshen were identified (Supplementary Table 1). After removing duplicates and applying the aforementioned filtering criteria, 6 components did not have corresponding targets, resulting in the final selection of 59 candidate components of Danshen (Supplementary Table 2). Utilizing TCMSP predictions, a total of 930 targets for Danshen candidate components were collected. Following the removal of duplicates, 123 targets and their respective symbols were obtained (Supplementary Table 3). We introduced 59 candidate components and 123 targets into Cytoscape and constructed a Danshen-components-targets network (Fig. 2A), containing 183 nodes and 989 edges. To further investigate the relationship between Danshen and COVID-19, we utilized the Genecards database and identified 1355 potential targets by inputting "novel coronavirus pneumonia" as a query. After applying the filtering standards mentioned above, 497 targets were eliminated, and 858 COVID-19-related targets were retained (Supplementary Table 4). By employing a Venn diagram (Fig. 2B), an intersection was generated between the 123 targets of Danshen candidate components and the 858 COVID-19-related targets, resulting in 31 common targets (Supplementary Table 5). These common targets were considered potential targets for Danshen in treating COVID-19 and were utilized for subsequent network construction and pathway enrichment analysis. The 31 common targets and corresponding to 45 components were used to construct the Danshen-45 components-31 common targets-COVID-19 network (Fig. 2C), which consisted of 183 nodes and 989 edges. Nodes with higher degrees, as indicated by a greater number of edges, exhibited larger sizes within the network, signifying their greater importance. Network analysis revealed a threshold degree value of5.6 for the 45 candidate components. Table 1 displays the top 10 compound nodes with the largest degree sizes. Among them, 6 components exhibited degrees higher than 5.6: (MOL000006, degree = 19), (MOL007154, degree = 12), (MOL007111, degree = 8), (MOL007050, degree = 8), (MOL007100, degree = 7), (MOL007088, degree = 6). Thus, these compounds are deemed critical nodes within the network and hold significant potential for treating COVID-19, suggesting that Danshen exerts its effects on COVID-19 through multiple components.

**Table 1:** The top10 candidate components in degree value.

MOL ID	Molecule name	Degree
MOL000006	Luteolin	19
MOL007154	Tanshinone IIA	12
MOL007111	Isotanshinone II	8
MOL007050	2-(4-hydroxy-3-methoxyphenyl)-5-(3-hydroxypropyl)-7-methoxy-3-benzofurancarboxaldehyde	8
MOL007100	dihydrotanshinlactone	7
MOL007088	cryptoTanshinone	6
MOL007119	miltionone I	5
MOL007108	isocryptotanshi-none	5
MOL007127	1-methyl-8,9-dihydro-7H-naphtho[5,6-g]benzofuran-6,10,11-trione	5
MOL007124	neocryptotanshinone II	5

### PPI network construction

**Figure 3:**
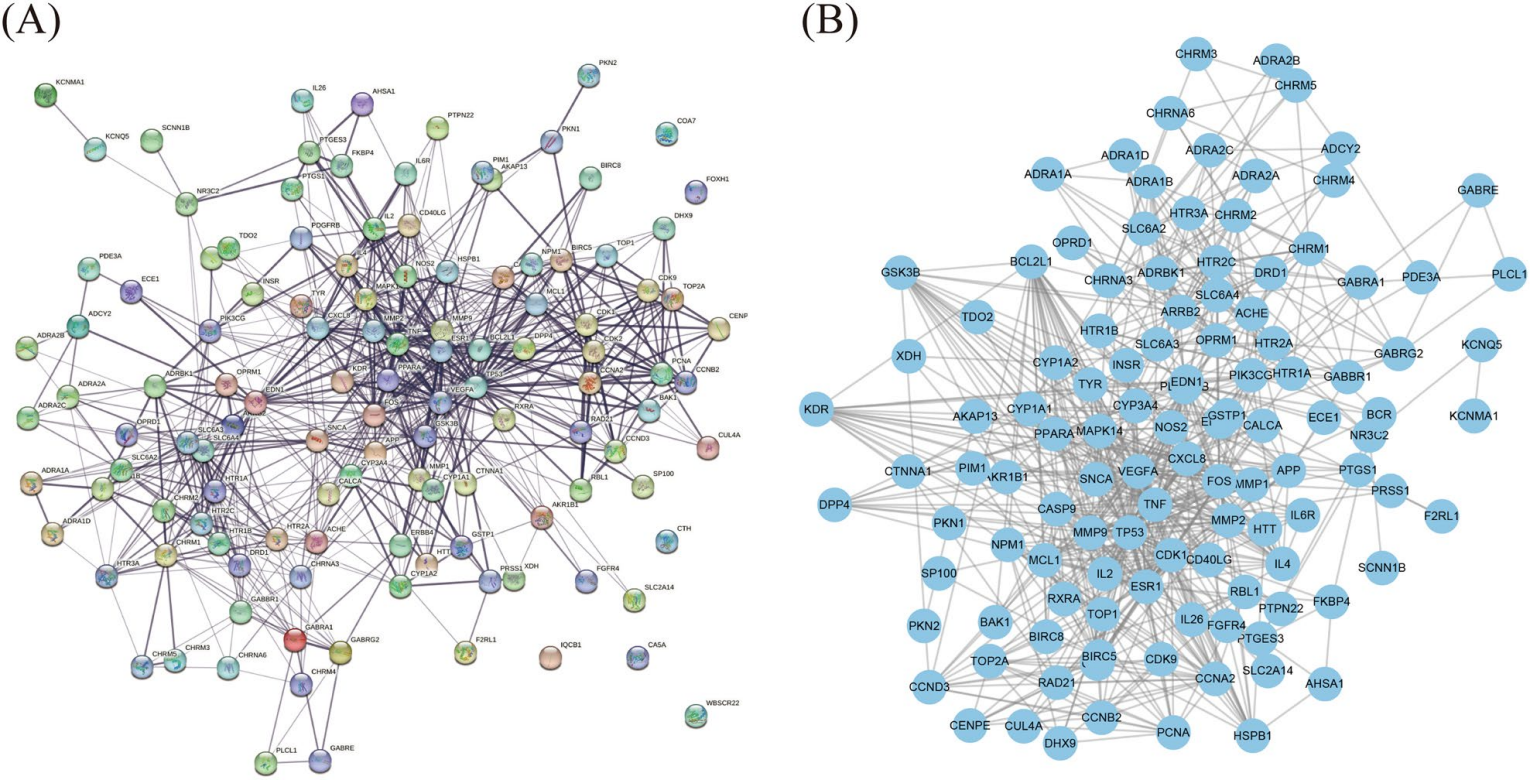
Protein–Protein interaction (PPI) network construction of the candidate components of Danshen. (**A**) Targets PPI network of candidate components in STRING. (**B**) Cytoscape-processed of Targets' PPI network of candidate components. Blue Ellipse represents targets.

**Figure 4:**
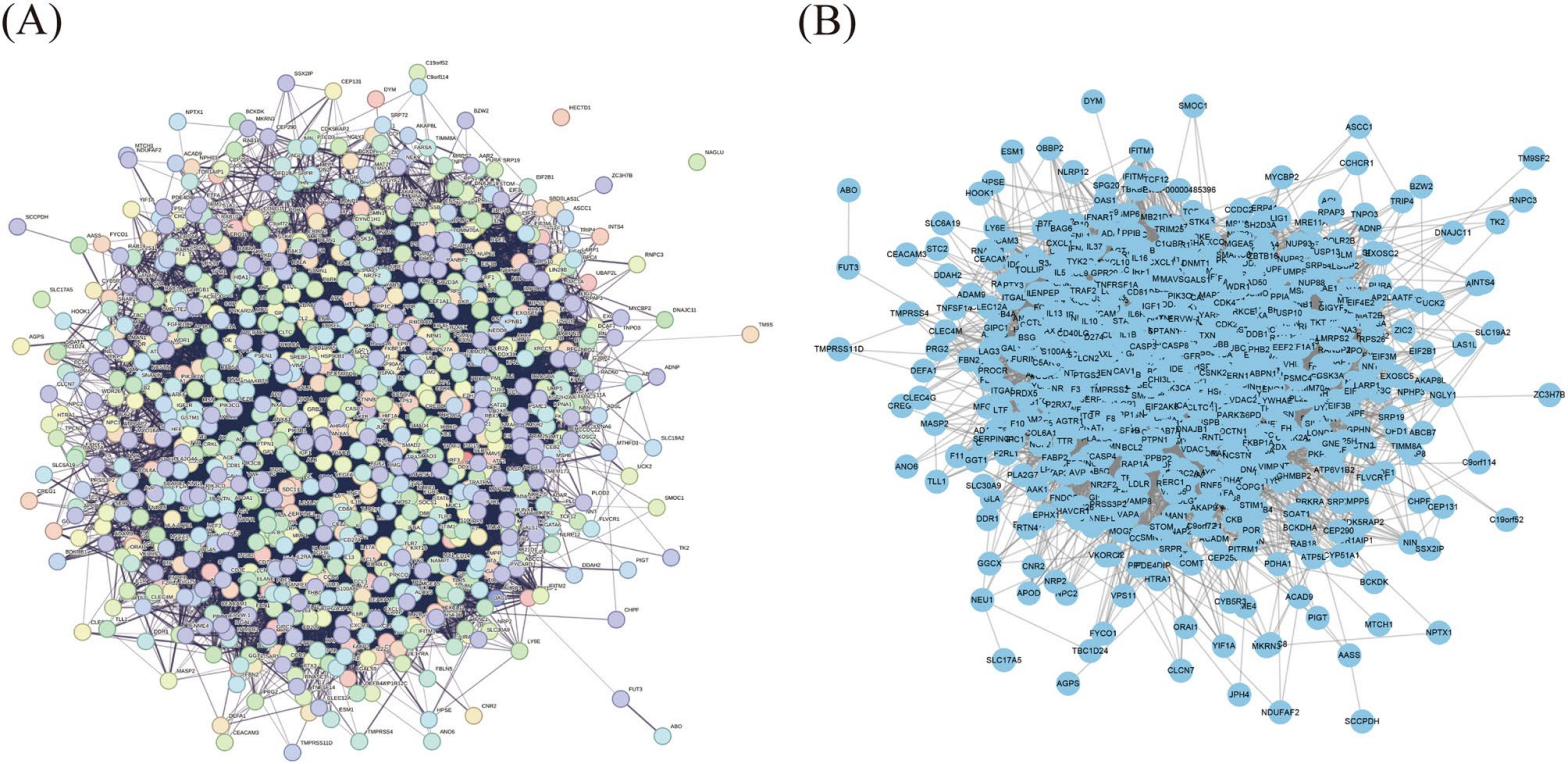
Protein–Protein interaction (PPI) network construction of COVID-19-related targets. (**A**) Targets PPI network of COVID-19 in STRING. (**B**) Cytoscape-processed of Targets PPI network of COVID-19. Blue Ellipse represents targets.

**Figure 5:**
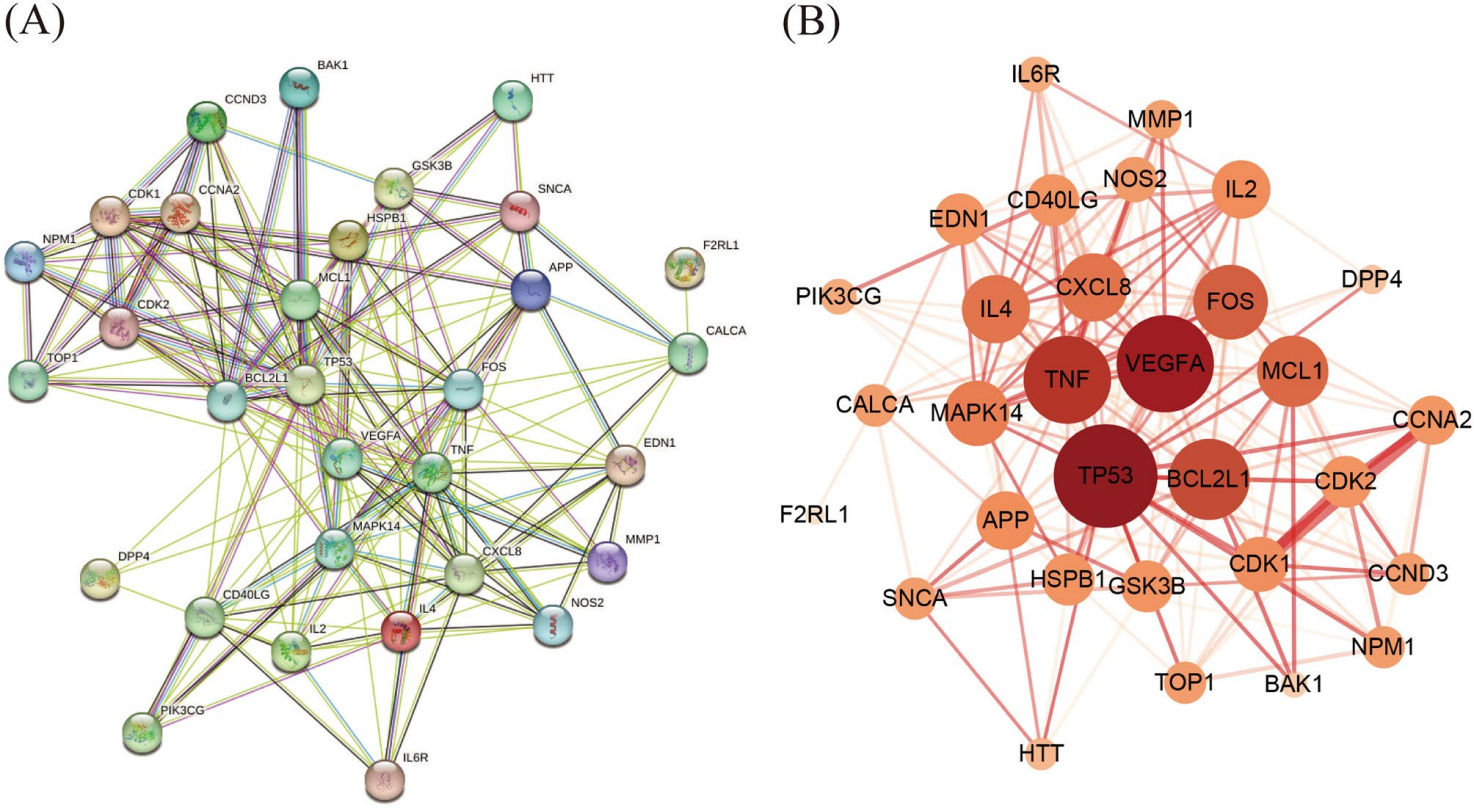
Protein–Protein interaction (PPI) network construction of common targets between candidate components of Danshen and COVID-19. (**A**) Targets PPI network of common targets in STRING. (**B**) Cytoscape-processed Targets PPI network of common targets. Nodes from small to large represent the degree value from low to high. The deeper color represents the higher degree of targets.

**Figure 6:**
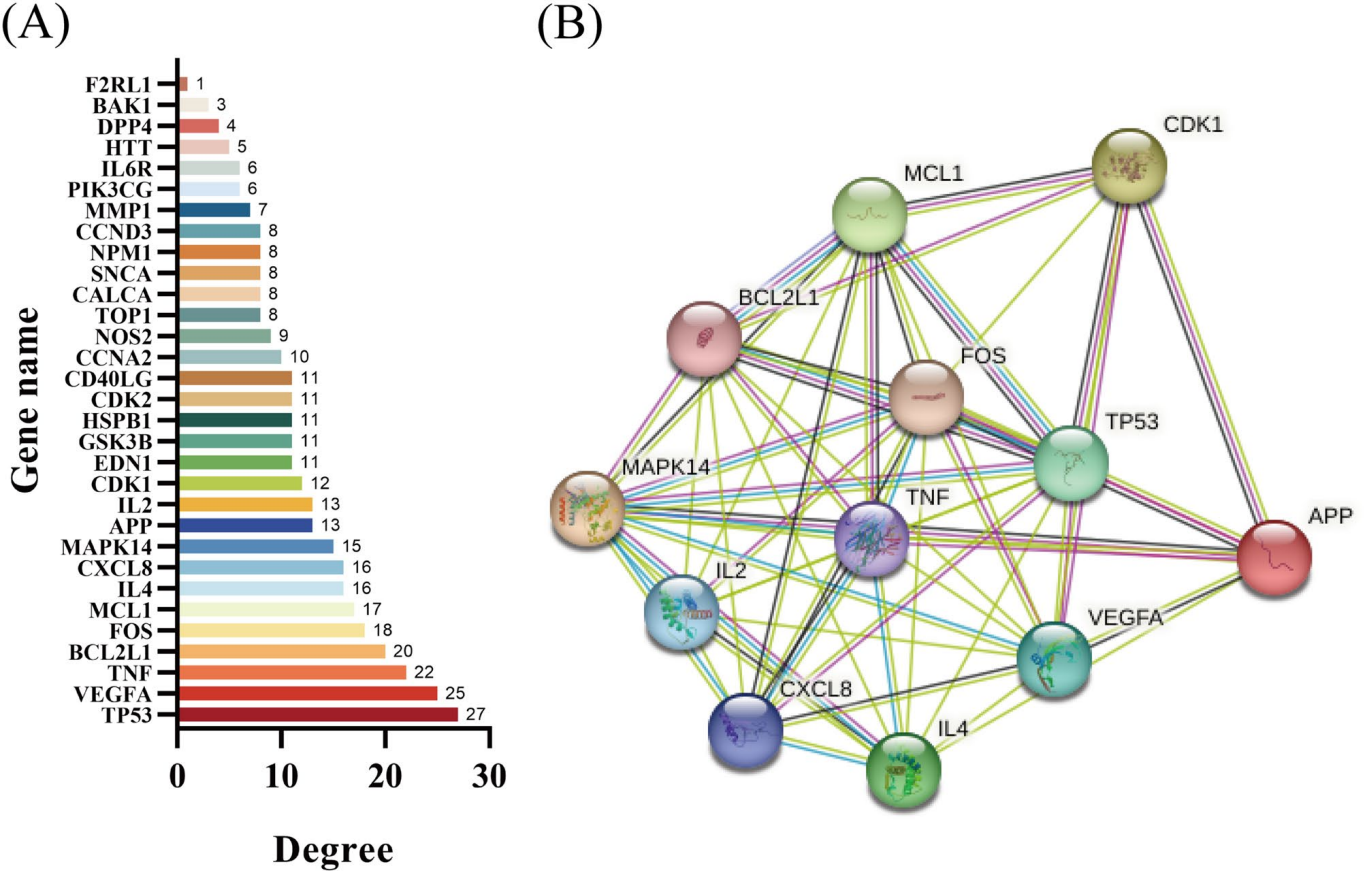
Degree value of core targets and Protein–Protein interaction (PPI) network construction of core targets. (**A**) Degree value of core targets. (**B**) PPI network of core targets.

The PPI network was used to understand the correlation between various targets in Danshen and COVID-19. In this study, we constructed 123 targets PPI network of 59 candidate components (Fig. 3A), then introduced data from STRING to Cytoscape to acquire a visualization result based on the degree value (Fig. 3B). The result showed that the candidate components targets PPI network had 115 nodes and 687 edges. In addition, we constructed the COVID-19-related targets network with PPI data (Fig. 4A) and developed a Cytoscape-processed result (Fig. 4B). The result indicated that the COVID-19 targets PPI network had 838 nodes and 28,307 edges. Then the common targets PPI network (Fig. 5A) and Cytoscape-processed result (Fig. 5B) was established, which consists of 31 nodes and 180 edges. A bigger node and deeper color indicate a greater degree of value. The edge thickness stands for the edge betweenness, and a thicker line indicates a stronger relationship between the two targets. The degree value of each target was shown in (Fig. 6A), and the degree value threshold of common targets' PPI network was 11.61, calculated from Cytoscape-Centiscape 2.2 Menu. Topological analysis of the common targets PPI networks identified 12 genes with scores higher than the threshold as the core targets, following TP53, TNF, VEGFA, APP, MAPK14, IL4, CDK1, FOS, BCL2L1, MCL1, IL2, CXCL8. Then the PPI network of all core targets was produced (Fig. 6B). These dates suggested that candidate components probably acted on COVID-19 mainly through the targets of TP53, TNF, VEGFA, APP, MAPK14, IL4, CDK1, FOS, BCL2L1, MCL1, IL2, CXCL8.

### KEGG enrichment analyses

**Figure 7:**
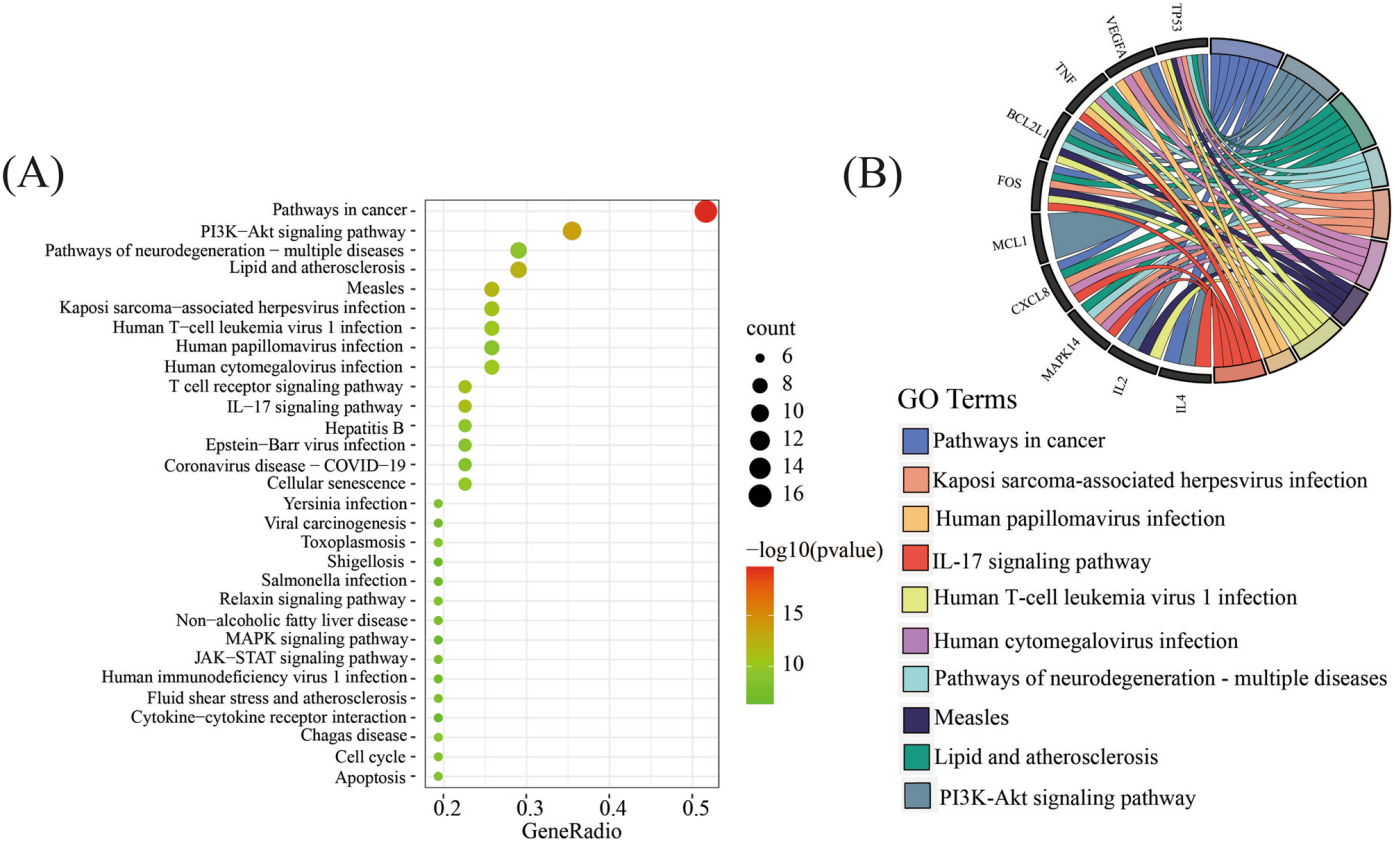
Top 30 most meaningful pathways in KEGG enrichment analysis of common targets with the basis set as P < 0.05. (**A**) Dot plot of KEGG enrichment. The color changes from red to green, indicating that the − log10 (p value) changes from large to small. The larger the surface area, the greater the enrichment degree. (**B**) Circle diagram. The genes are linked to their assigned terms via different colored ribbons, the larger ribbons, the more targets were enriched in pathways.

To investigate the underlying biological processes associated with Danshen treatment for COVID-19, a KEGG analysis was performed on 31 common targets. The analysis identified 96 statistically significant pathways (Supplementary Table 6), from which the top 30 pathways with the highest count value were selected for further analysis (Fig. 7A). These pathways primarily encompassed Pathways in cancer, PI3K-Akt signaling pathway, Lipid and atherosclerosis, Pathways of neurodegeneration-multiple diseases, Kaposi sarcoma-associated herpesvirus infection, Human cytomegalovirus infection, Measles, Human T-cell leukemia virus 1 infection, Human papillomavirus infection, IL-17 signaling pathway, T cell receptor signaling pathway, Hepatitis B, Coronavirus disease—COVID-19, Cellular senescence, Epstein-Barr virus infection, JAK-STAT signaling pathway, Chagas disease, Yersinia infection, Fluid shear stress, and atherosclerosis, Non-alcoholic fatty liver disease, Shigellosis, Salmonella infection, MAPK signaling pathway, Cell cycle, Viral carcinogenesis, Toxoplasmosis, Relaxin signaling pathway, Apoptosis, Human immunodeficiency virus 1 infection, and Cytokine-cytokine receptor interaction. The observed prominence of Pathways in cancer and the PI3K-Akt signaling pathway suggests that Danshen may exert its therapeutic effects on the target disease primarily through its interactions with Pathways in cancer or the PI3K-Akt signaling pathway. Furthermore, a circle diagram was constructed to examine the enrichment analysis of the top 10 KEGG pathways among the 12 core genes (Fig. 7B). The results indicated a significant enrichment of the core genes in pathways related to cancer. In summary, the KEGG analysis of Danshen treatment on COVID-19 identified several key pathways, with a notable emphasis on Pathways in cancer and the PI3K-Akt signaling pathway. Additionally, the enrichment analysis of core genes further supported their involvement of Pathways in cancer.

### GO enrichment analysis

**Figure 8:**
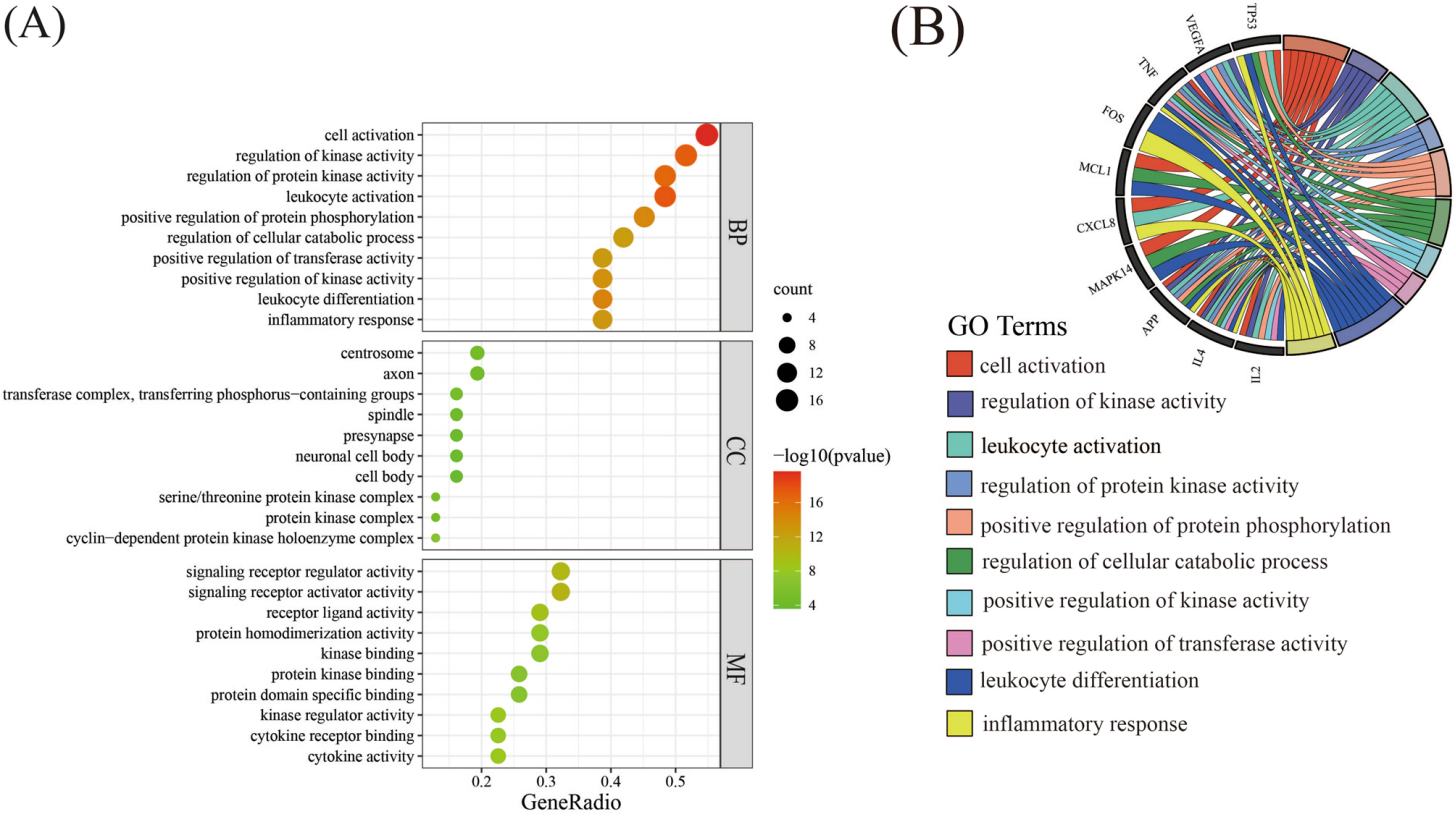
Top thirty most meaningful pathways in GO enrichment analysis of common targets with the basis set as P < 0.05. (**A**) Dot plot of GO enrichment. The color changes from red to green, indicating that the − log10 (p value) changes from large to small. The larger the surface area, the greater the enrichment degree. (**B**) Circle diagram. The genes are linked to their assigned terms via different colored ribbons, the larger ribbons, the more targets were enriched in BP pathways.

**Figure 9:**
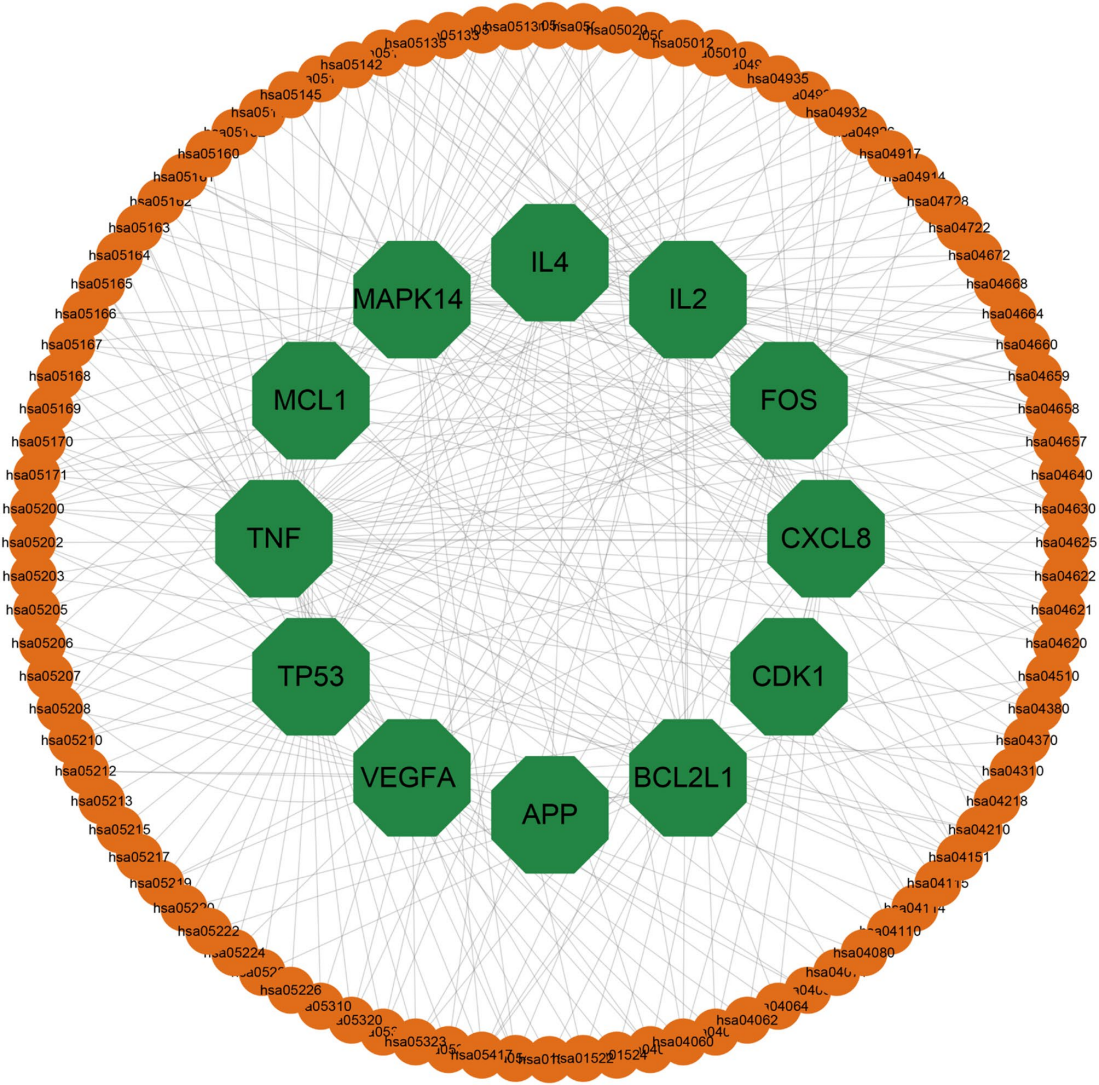
Core targets-pathways. Green octagon represents core targets. Yellow Ellipse represent enriched pathways.

**Figure 10:**
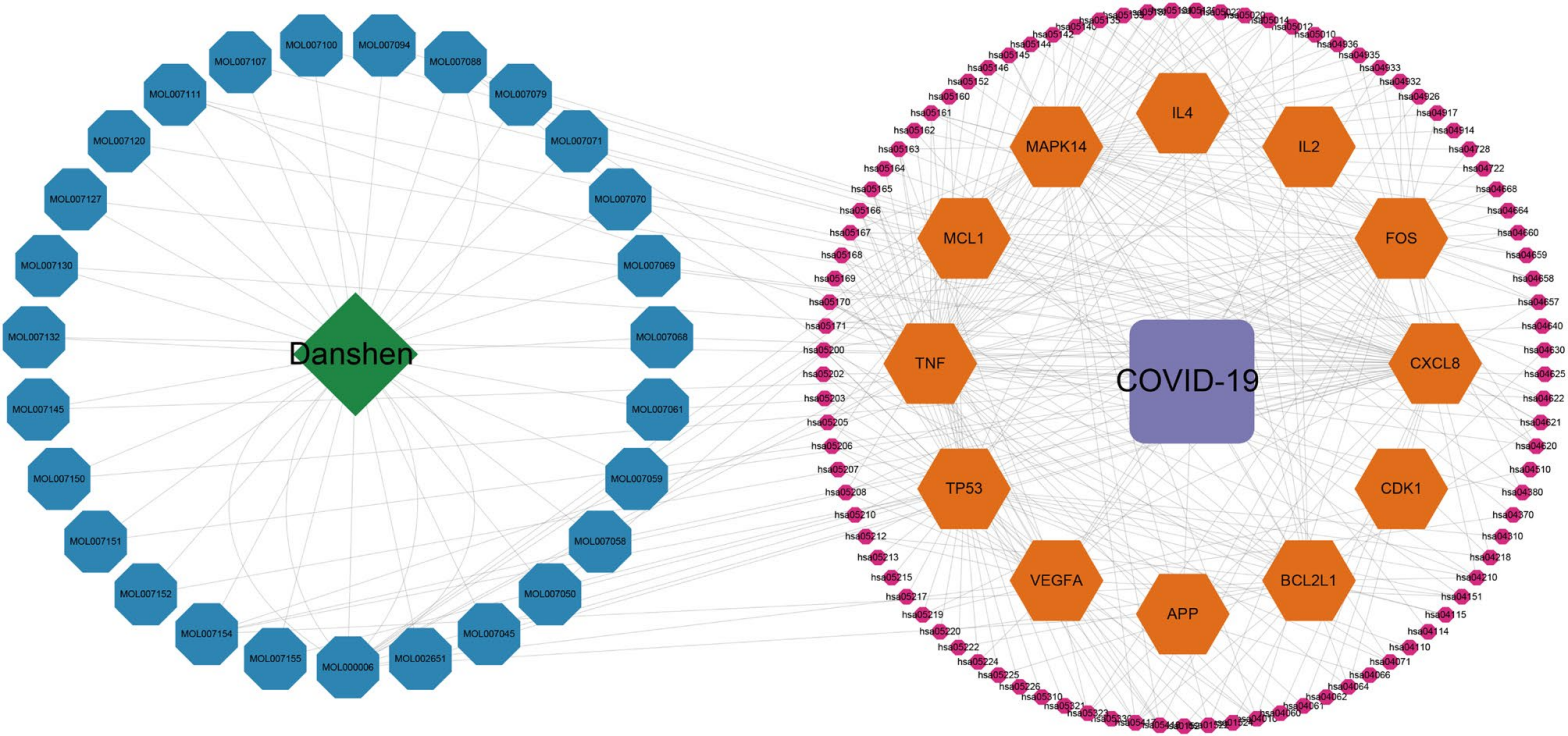
Danshen-compounds-core targets-KEGG pathways-COVID-19.The green diamond represents Danshen. The blue hexagon represents candidate components. Magenta represents enriched pathways. Yellow represents core targets. Purple represents COVID-19.

The GO analysis encompassed the exploration of biological processes (BP), cellular components (CC), and molecular functions (MF) in this study. 764 statistically significant GO terms were derived (Supplementary Table 7), comprising 696 BP terms, 31 CC terms, and 37 MF terms. The visualization of the top 10 highly abundant enrichment terms for BP, CC, and MF (Fig. 8A) facilitated the interpretation of the results. The analysis of biological processes (BP) unveiled the prominent involvement of the common genes in various cellular activities, including cell activation, regulation of kinase activity, leukocyte activation, regulation of protein kinase activity, positive regulation of protein phosphorylation, regulation of cellular catabolic process, positive regulation of kinase activity, positive regulation of transferase activity, leukocyte differentiation, and inflammatory response. The analysis of cellular components (CC) identified the centrosome, axon, transferase complex, and phosphorus-containing groups as the primary cellular locations where the targets were concentrated. In terms of molecular functions (MF), the main functional roles encompassed signaling receptor activator activity, signaling receptor regulator activity, and receptor-ligand activity. Subsequently, a circular diagram (Fig. 8B) was constructed to elucidate the enrichment analysis of biological process pathways among the 12 core genes, revealing a notable enrichment of leukocyte differentiation among the core genes. To further investigate the intricate relationship between Danshen, its compounds, core targets, pathways, and COVID-19, two network diagrams were devised. The "Core targets-BP pathways" network (Fig. 9) and the "Danshen-compounds-core targets-KEGG pathways-COVID-19" network (Fig. 10) provided a comprehensive visualization of the interconnections, underscoring the multi-faceted nature of Danshen's actions, involving multiple targets and diverse components.

### Molecular docking

**Figure 11:**
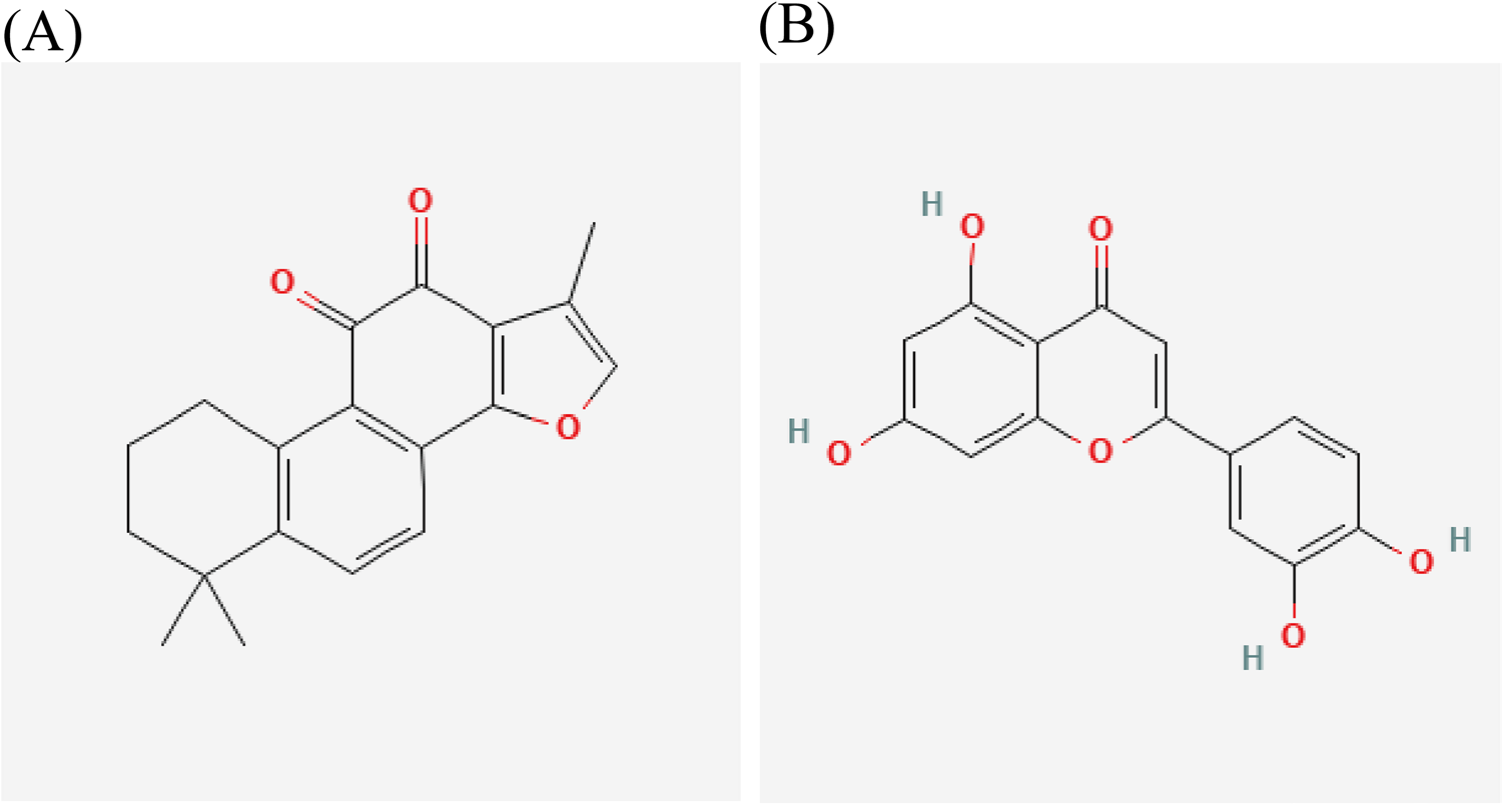
2D structure of components. (**A**) 2D structure of Tanshinone IIA. (**B**) 2D structure of Luteolin.

**Figure 12:**
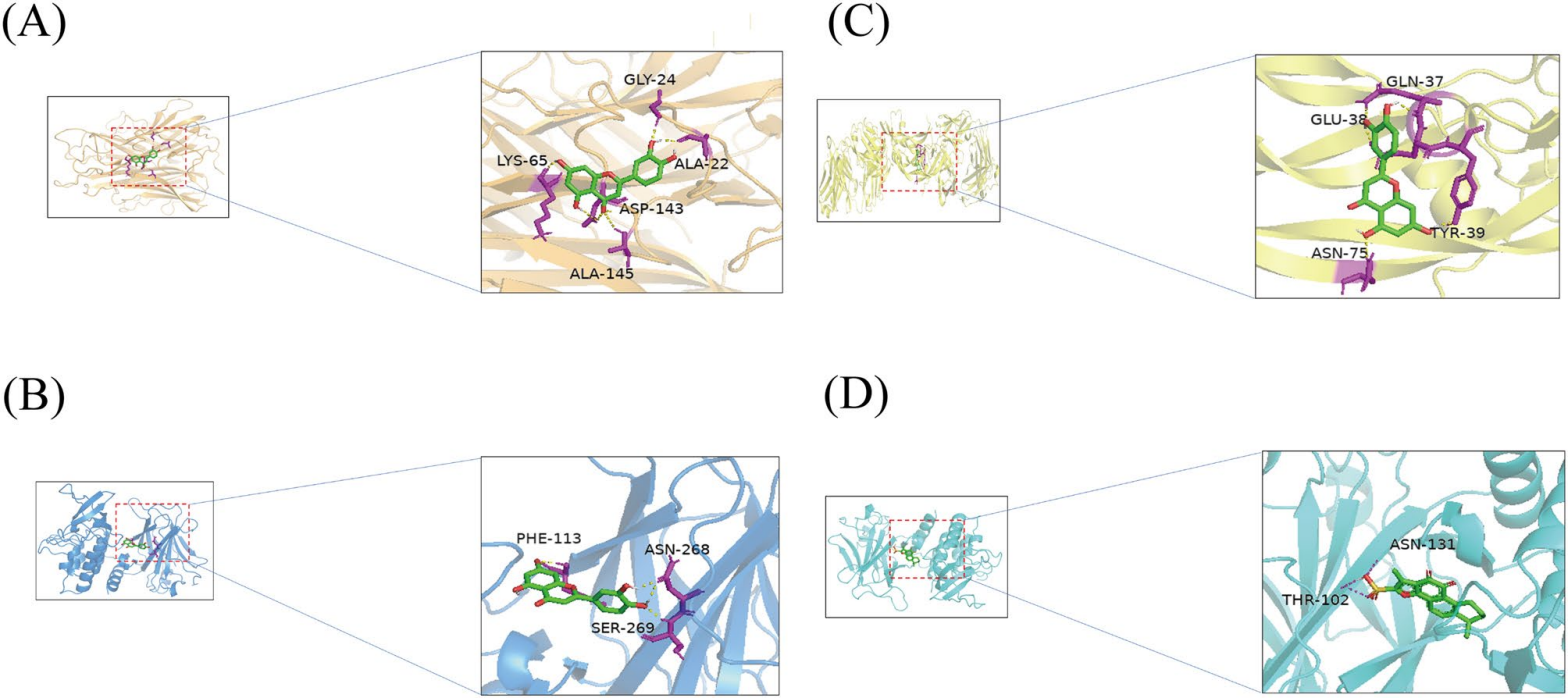
Molecular docking verification. (**A**) Luteolin bound to the candidate pocket of TNF. (**B**) Luteolin bound to the candidate pocket of TP53. (**C**) Luteolin bound to the candidate pocket of VEGFA. (**D**) Tanshinone IIA bound to the candidate pocket of TP53.

The PPI network analysis identified the top 3 targets among the core targets, namely TP53, VEGFA, and TNF. These targets were selected for molecular docking with their corresponding compounds. Specifically, TP53 corresponded to Luteolin and Tanshinone IIA, VEGFA corresponded to Luteolin, and TNF corresponded to Luteolin. The 2D structures of Tanshinone IIA and Luteolin can be found in Fig. 11. The binding affinities of these complexes were evaluated and presented in Table 2. The results of the matching analysis demonstrated that top 3 targets and compound molecules exhibited docking scores below − 5. Notably, three targets structures were utilized: TP53 (PDB ID: 6RZ3), VEGFA (PDB ID: 4zff), and TNF (PDB ID: 1A8M). Luteolin ligand formed five hydrogen bonds with a docking score of − 6.55 kcal/mol against 1A8M (Fig. 12A), three hydrogen bonds with a docking score of − 7.19 kcal/mol against 6RZ3 (Fig. 12B), and four hydrogen bonds with a docking score of − 5.1 kcal/2 mol against 4ZFF (Fig. 12C). On the other hand, Tanshinone IIA ligand formed three hydrogen bonds with a docking score of − 5.29 kcal/mol against 6RZ3 (Fig. 12D).

**Table 2:** Docking results of top3 core targets with corresponding components.

Components	Targets	E-value (Kcal/mol)	NO. H bonds	Binding site
Luteolin	TNF(PDB:1A8m)	− 6.5	5	ALA:22 ASP:143 LYS:65 GLY:24 ALA:145
Luteolin	TP53(PDB:6RZ3)	− 7.19	3	PHE:113 ASN:268 SER:269
Tanshinone IIA	TP53(PDB:6RZ3)	− 5.29	2	THR:102 ASN:131
Luteolin	VEGFA(PDB:4Zff)	− 5.1	4	GLN:37 GLU:38 TYR:39 ASN:75

### Root-mean-square deviations

**Figure 13:**
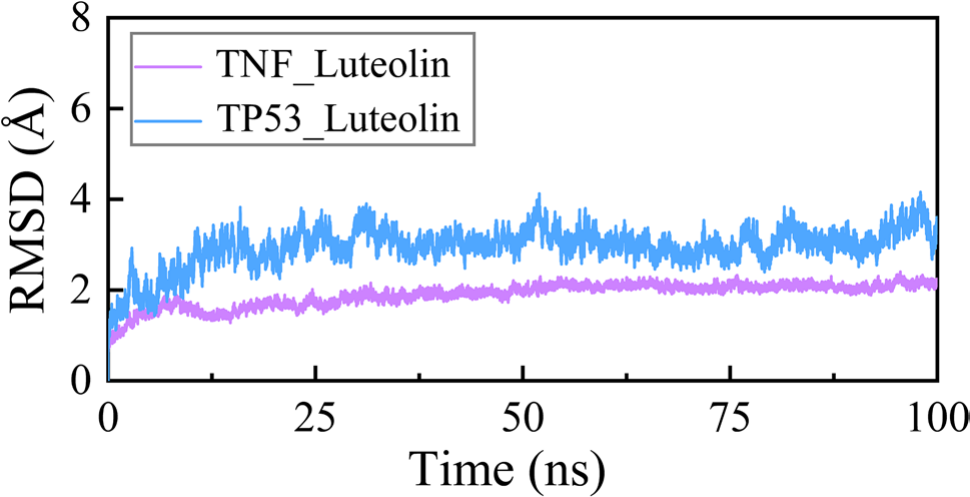
The RMSDs of TNF-Luteolin and TP53-Luteolin during the molecular dynamics simulations.

We selected the TNF-Luteolin and TP53-Luteolin to perform molecular dynamics simulations. The root-mean-square deviations (RMSDs) of the Cα atoms of TNF-Luteolin and TP53-Luteolin were calculated by aligning to their first frame to evaluate the conformational stability of each simulated system (All raw data can be found from Supplementary_Raw data_Molecular Dynamics Simulations). As illustrated in Fig. 13, two simulations converged and displayed RMSD values smaller than 4 Å over the last 50 ns, indicating the high stability of TNF-Luteolin and TP53-Luteolin systems. In addition, TNF-Luteolin exhibited a smaller RMSD value (~ 2 Å) than that of TP53-Luteolin (~ 3.5 Å), indicating TNF-Luteolin should more stable than TP53-Luteolin during the MD simulations. To further evaluate the binding affinity between two proteins and luteolin, the MM-GBSA method was utilized to calculate the binding free energy for the TNF-Luteolin and TP53-Luteolin over the last 20 ns MD trajectories. As listed in Table 3, the binding free energies (ΔGMM/GBSA) were − 11.31, and − 19.84 kcal/mol for TNF-Luteolin and TP53-Luteolin, respectively. Hence, luteolin exhibited a higher binding affinity to TNF compared to TP53. In detail, the van der Waals (ΔEvdw) interaction and the direct electrostatic interaction (ΔEele) were the crucial driving force for the combination between protein and luteolin. In contrast, the polar component (ΔGpol,sol) was unconducive to the ligand binding.

**Table 3:** Components of the binding free energy between two proteins and luteolin averaged in the Last 20 ns (kcal/mol).

Contributions	TNF-Luteolin	TP53-Luteolin
Δ*E*_vdw_	− 12.71	− 27.61
Δ*E*_ele_	− 49.18	− 17.89
Δ*G*_pol,sol_	52.97	29.39
Δ*G*_npol,sol_	− 2.39	− 3.74
Δ*E*_MM_	− 61.89	− 45.50
Δ*G*_sol_	50.58	25.65
Δ*G*_MM/GBSA_	− 11.31	− 19.84

### Enrichment calculations

**Figure 14:**
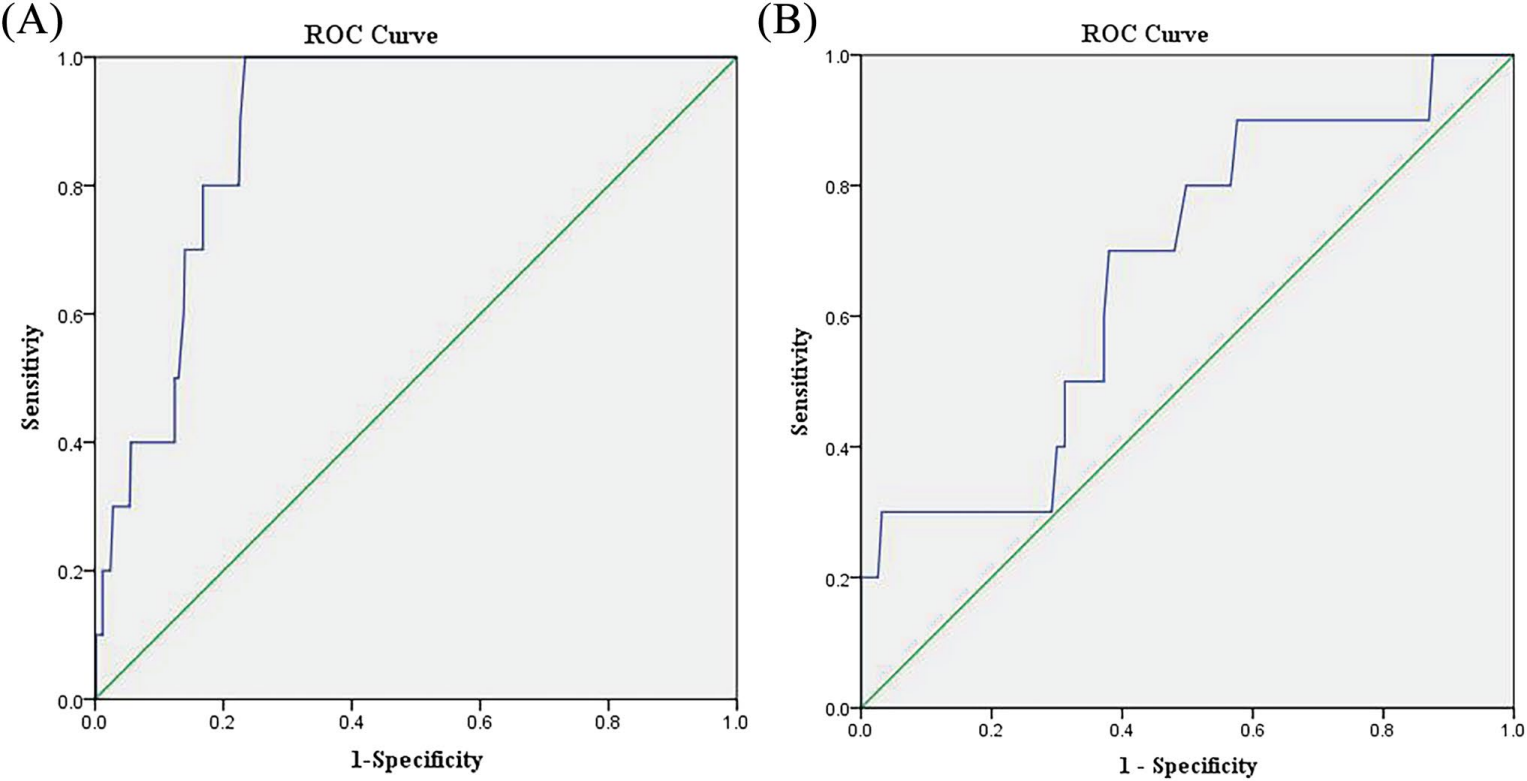
The plot of receiver operator characteristic (ROC) area under the curve: (**A**) Enrichment result for TP53. (**B**) Enrichment result for TNF.

To ensure reliable results and avoid false-positive hits during the docking analysis, enrichment calculations were performed using a set of 500 decoy compounds obtained from the DUD.E database. For the calculation of the enrichment factor, a total of 10 compounds from each enumeration along with Luteolin were selected. These 10 compounds were mixed with the 500 decoy sets and re-docked using the Autodock protocol against the TP53 and TNF receptors. To assess the performance of the docking protocol, a validation factor, such as the receiver operating characteristic (ROC) curve, was calculated. The results obtained from this analysis are summarized in Supplementary Tables 8 and 9. For the TP53 receptor, the results showed that AUC = 0.899 (Fig. 14A). For the TNF receptor, the results showed that AUC = 0.688 (Fig. 14B).

The ROC curve plot confirms the specificity and sensitivity of the employed method in identifying active compounds.

## Discussion

COVID-19, a novel respiratory infectious disease, has rapidly spread worldwide, resulting in significant public health consequences^42^. Clinical observations have indicated that common manifestations in COVID-19 patients include fever, cough, fatigue, and myalgia^43^. Currently, vaccination have reduced the risk of death related to COVID-19 to some extent, but the significant sequelae, such as lung damage, pulmonary fibrosis, myocarditis arrhythmias and stroke are still exist^44^. Traditional Chinese medicine (TCM) has demonstrated potential antiviral properties against various viruses^45^, such as herpes simplex virus, influenza virus, and human immunodeficiency virus^46–48^. Previous studies have also suggested the therapeutic potential of Danshen, a TCM herb, in the context of COVID-19^49^. Moreover, as stated in the Protocol (National Health Commission of the People's Republic of China, 2020. Diagnosis and Treatment Protocol for COVID-19 Trial Version 7), Danshen has been actively employed in clinical settings for managing COVID-19. Specially, during the critical phase of clinical treatment, the administration of Xuebijing injection, comprising Safflower, Red Peony Root, Szechwan Lovage Rhizome, Danshen, and *Angelica sinensis*, was advised for the management of the disease. However, a comprehensive understanding of the detailed mechanisms underlying Danshen's effectiveness against COVID-19 is still lacking.

Network pharmacological analysis offers a promising approach to explore the impact of TCM on diseases by systematically elucidating the complex components, targets, and mechanisms involved. Therefore, in this study, our aim was to explore the potential therapeutic effects of Danshen in the treatment and mitigation of COVID-19 using network pharmacology. We aimed to identify the core targets, biological pathways, and potential mechanisms of Danshen in the treatment of COVID-19. Additionally, in order to assess the reliability of the binding, we calculated the docking score between Danshen components and their corresponding targets, which will provide new insights for the treatment of COVID-19 and vaccine development.

Specifically, our investigation identified 59 candidate components of Danshen that may be effective in treating COVID-19, with Luteolin emerging as the most promising compound due to it’s the largest number of targets interactions in target prediction and network construction. Luteolin is a naturally occurring flavonoid found in various plants^50^, and it possesses various pharmacological activities, including anti-inflammatory^51^, anti-tumor^52^, and anti-allergy effects^53^. Existing studies have demonstrated that Luteolin can attenuate LPS-induced myocardial injury by activating AMPK-mediated autophagy^54^, suppress THP-1 macrophage pyroptosis by inhibiting ROS production through Nrf2 activation and NF-κB inactivation^55^, and mitigate breast cancer hallmarks by downregulating Nrf2-mediated gene expressions^56^. Notably, Luteolin has also shown potential in alleviating long-COVID syndrome symptoms, such as cognitive dysfunction and fatigue, likely through its anti-inflammatory effects^57^. These findings, along with our results underscore the significance of Luteolin in the context of Danshen's efficacy against COVID-19.In addition to Luteolin, other candidate components derived from Danshen exhibited pharmacological activities relevant to COVID-19. Among them, Tanshinone IIA displayed a higher degree value in target prediction and network construction. Tanshinone IIA is a fat-soluble quinone compound widely used in the treatment and adjuvant therapy of cardiovascular diseases^58,59^, non-alcoholic fatty liver disease^60^, and cancers^61^. Notably, Tanshinone IIA has been shown to suppress gastric cancer proliferation by inducing ferroptosis, inhibit glucose metabolism leading to apoptosis in cervical cancer, and promote cell apoptosis in ovarian carcinoma cells through direct upregulation of miR-205^62^. Molecular docking studies further revealed that Tanshinone IIA exhibited favorable interactions with spike and main protease receptors, indicating its intrinsic anti-SARS-CoV-2 potency^63^. Moreover, our screening process identified 31 common targets shared by Danshen components and COVID-19, highlighting the different targets of Danshen relevant to COVID-19. Through topological analysis, we identified 12 core targets among these 31 common targets for further investigation. Analysis of protein–protein interaction networks indicated that TP53, VEGFA, and TNF were the top three core targets. These targets are primarily associated with inflammation, suggesting that Danshen may exert an anti-inflammatory effect. Molecular docking results showed that all the docking scores were below -5. Then we selected the two lowest docking scores results to perform molecular dynamics, the results showed high stability of TNF-Luteolin and TP53-Luteolin systems, and TNF-Luteolin showed more stable than TP53-Luteolin during the molecular dynamics simulations. To further evaluate the binding affinity between two proteins and luteolin, we use the MM-GBSA method to calculate the binding free energy for the TNFLuteolin and TP53-Luteolin over the last 20 ns molecular dynamics trajectories. luteolin exhibited a higher binding affinity to TNF compared to TP53.

Importantly, TP53, which is crucial in regulating tumor immune responses, plays a multifaceted role in cancer development and therapy^63^. Notably, TP53 has also been identified as a hub protein in the context of Yinqiao powder, another TCM formulation with potential effects against COVID-19^64^. Additionally, TP53 has been implicated as a pivotal target of puerarin, a TCM compound with potential COVID-19 treatment effects^65^. These findings further support the importance of TP53 in the context of Danshen's efficacy against COVID-19.

Furthermore, TNF and VEGFA, which exhibited higher degree values in target prediction and network construction, may also play significant roles in Danshen's therapeutic effects against COVID-19. TNF is a crucial mediator and regulator of immune responses in both healthy and diseased conditions^66^. It can induce programmed cell death (PCD) through apoptosis and/or necroptosis^67^. TNF has been identified as a core gene target in Lithospermum erythrorhizon against COVID-19, and Tormentic acid, a primary component of Lithospermum erythrorhizon, has shown strong binding affinity with TNF^68^. Similarly, VEGFA is a key regulator of angiogenesis and vascular permeability^69^. Interestingly, VEGFA has also been identified as a core target in Scutellaria baicalensis, another TCM herb with potential therapeutic effects against COVID-19^70^. Overall, the network pharmacology analysis and molecular docking results highlight the potential of Danshen and its components, particularly Luteolin and Tanshinone IIA, in the treatment of COVID-19. These components exhibit interactions with core targets involved in inflammation, immune response, and viral infection.

The docking results demonstrated that Luteolin exhibited binding affinity towards TP53, VEGFA, and TNF, while Tanshinone IIA showed specific binding to TP53.Regarding Luteolin, we observed that it formed five hydrogen bonds with TNF, as shown in Fig. 12A, resulting in a docking score of -6.55 kcal/mol. For TP53, Luteolin formed three hydrogen bonds, as depicted in Fig. 12B, with a docking score of -7.19 kcal/mol. Additionally, Luteolin formed four hydrogen bonds with VEGFA, as illustrated in Fig. 12C, with a docking score of -5.1 kcal/mol. These hydrogen bonding interactions contribute to the stability and affinity of Luteolin binding to the respective target proteins. In the case of Tanshinone IIA, we observed that it formed three hydrogen bonds with TP53, as shown in Fig. 12D, resulting in a docking score of -5.29 kcal/mol. These hydrogen bonding interactions play a crucial role in stabilizing the Tanshinone IIA-TP53 complex.The docking scores obtained for the top three targets and compound molecules were below -5, indicating strong binding affinity. Additiconally, The findings from this docking validation protocol demonstrate the satisfactory performance of the autodock docking protocol. The ROC curve plot further validates the specificity and sensitivity of the employed method in identifying active compounds.

In comparison to the published paper by Simon J. L. Petitjean^49^, which demonstrated the effects of Danshen extracts against COVID-19 by blocking the binding of SARS-CoV-2 to the ACE2 receptor and alleviating the inflammatory response from leukocytes by interfering with NF-κB signaling, our study fills the gap by investigating multiple targets and pathways in a comprehensive and systematic manner. Additionally, a study conducted by Shiling Hu^17^ only revealed that three Salvianolic acids can inhibit the entry of 2019-nCoV spike pseudovirus into ACE2 high expression cells by binding to the RBD of the 2019-nCoV spike protein and ACE2 protein, which prompted us to explore the potential of Danshen's ingredients treating COVID-19 in a comprehensive and systemic way. Based on this, network pharmacology holds great potential in elucidating the intricate connections among multiple ingredients from Danshen, multiple targets, and COVID-19, which also offers a novel research approach for studying diseases related to traditional Chinese medicine.

However, it is worth mentioning that the potential active components, possible targets, and important biological pathways of Danshen in the treatment of COVID-19 is based on computational technologies and still requires further validation through pharmacological and preclinical studies.

Due to the spike protein of the 2019-nCoV virus facilitates the entry of the virus into cells, particularly respiratory epithelial cells, by binding to the angiotensin‐converting enzyme 2 (ACE2) receptor, leading to infection and the development of the disease^71–73^, we want to explore whether ingredients from Danshen could play inhibition potential in binding to ACE2 and spike protein. We set out to evaluate the toxicity of Tanshinone IIA on ACE2 high‐expressing HEK293T cells. Moreover, we further need to evaluate the binding capacity of Tanshinone IIA to ACE2 and the spike protein of 2019‐nCoV using molecular docking and surface plasmon resonance. Additionally, many researchers have shown that 2019-nCoV can induce acute inflammatory response like acute lung inflammation^42,74,75^, we set out to establish a mouse model of acute lung inflammation infected with SARS-CoV-2 S according to the previous study^76^, and determine the pathological alterations in mice with oral and intravenous pretreatment with Tanshinone IIA dose-dependently. We hope this work will contribute to developing novel therapeutic strategies for the treatment of COVID-19 from ingredients of Danshen.

### Supplementary Information

Supplementary Tables.

Supplementary Information 1.

## Data Availability

The datasets generated and/or analysed during the current study are available in the Supplementary Materials.

## Abbreviations

2019-nCoV: SARS-CoV-2
ACE2: Angiotensin‐converting enzyme 2
BP: Biological processes
CC: Cellular components
COVID-19: Coronavirus disease-2019
DL: Drug similarity
GO: Gene ontology
KEGG: Kyoto encyclopedia of genes and genomes
MD: Molecular dynamics
MF: Molecular functions
OB: Oral availability
PPI: Protein–protein interaction
PME: Particle mesh Ewald
ROC: Receiver operating characteristic
RMSDs: Root-mean-square deviations
SARS: Severe acute respiratory syndrome
TCM: Traditional Chinese medicine
WHO: World Health Organization
