# Supplementary material for: Exploring the potential mechanisms of Danshen against COVID-19 via network pharmacology analysis and molecular docking
Source: Sci Rep. 2024 Jun 4;14:12780. doi: 10.1038/s41598-024-62363-x (PMC11150561; doi:10.1038/s41598-024-62363-x)
Supplement: Supplementary file 1 — Supplementary Tables. [file 41598_2024_62363_MOESM1_ESM.docx]

**Exploring the Potential Mechanisms of Danshen Against COVID-19 Via Network Pharmacology Analysis and Molecular Docking**

Qiang Zhang^1^, Zongsuo Liang^1,^ Xiaoqing Wang^2^, Siyu Zhang^3^ ,Zongqi Yang^1^*

^1^ College of Life Sciences, Key Laboratory of Plant Secondary Metabolism and Regulation of Zhejiang Province, Zhejiang Sci-Tech University, Hangzhou 310018, China;

^2^ School of Art and Design, Zhejiang Sci-Tech University, Hangzhou 310018, China;

^3^Shaoxing Biomedical Research Institute of Zhejiang Sci-Tech University Co., Ltd, Zhejiang Engineering Research Center for the Development Technology of Medicinal and Edible Homologous Health Food, Shaoxing 312075, China

*Correspondence:

yangzongqi@zstu.edu.cn

Supplementary table 1. Initial components of Danshen

| Mol ID | Molecule Name | OB (%) | DL |
| --- | --- | --- | --- |
| MOL000105 | protocatechuic acid | 25.36646796 | 0.035092 |
| MOL001055 | 5-isopropyl-2-methylbicyclo[3.1.0]hex-2-ene | 47.19217569 | 0.043509 |
| MOL001103 | [(3R)-3,7-dimethylocta-1,6-dien-3-yl] acetate | 27.72617755 | 0.041546 |
| MOL000117 | Cymol | 27.20382673 | 0.022463 |
| MOL001219 | Satol | 27.27344509 | 0.11469 |
| MOL000128 | NERYLACETATE | 25.94000169 | 0.041771 |
| MOL001303 | (1R,2R,4S)-2,4-diisopropenyl-1-methyl-1-vinylcyclohexane | 5.578789823 | 0.060442 |
| MOL000131 | EIC | 41.90443602 | 0.14347 |
| MOL001394 | Oktadekan | 9.806386361 | 0.090617 |
| MOL001452 | protocatechualdehyde | 38.34952596 | 0.027536 |
| MOL001601 | 1,2,5,6-tetrahydrotanshinone | 38.74538672 | 0.35791 |
| MOL000162 | beta-Chamigrene | 31.99116089 | 0.080672 |
| MOL001659 | Poriferasterol | 43.82985158 | 0.75596 |
| MOL001771 | poriferast-5-en-3beta-ol | 36.91390583 | 0.75034 |
| MOL000019 | D-Camphene | 34.97921026 | 0.039017 |
| MOL001942 | isoimperatorin | 45.46424674 | 0.22524 |
| MOL000198 | (R)-linalool | 39.80430084 | 0.022686 |
| MOL000002 | cyanidol | 1.364642837 | 0.24431 |
| MOL002001 | Oleanolic acid deriv. | 14.24459152 | 0.70295 |
| MOL002014 | SPBio_002209 | 28.20655195 | 0.061178 |
| MOL000202 | Moslene | 33.01642148 | 0.02224 |
| MOL002153 | 1H-Cycloprop(e)azulen-7-ol, decahydro-1,1,7-trimethyl-4-methylene-, (1aR-(1aalpha,4aalpha,7beta,7abeta,7balpha))- | 82.32846156 | 0.12209 |
| MOL002222 | sugiol | 36.11353486 | 0.27648 |
| MOL002229 | HEPTACOSANE | 8.180710047 | 0.36155 |
| MOL000223 | caffeic acid | 25.76439804 | 0.050089 |
| MOL002376 | PENTACOSANE | 8.249355127 | 0.27262 |
| MOL000257 | (-)-beta-Phellandrene | 40.44454929 | 0.022312 |
| MOL000263 | oleanolic acid | 29.02084142 | 0.75599 |
| MOL002651 | Dehydrotanshinone II A | 43.76228599 | 0.40019 |
| MOL002771 | VIV | 14.26461321 | 0.55443 |
| MOL002776 | Baicalin | 40.12360996 | 0.75264 |
| MOL002835 | δ-cadinol | 17.12537144 | 0.077187 |
| MOL003047 | [(1S)-endo]-(-)-Borneol | 83.54491086 | 0.05275 |
| MOL000346 | succinic acid | 29.62033732 | 0.012082 |
| MOL000036 | beta-caryophyllene | 29.70229451 | 0.089893 |
| MOL003969 | L-Serin | 98.46910714 | 0.0091307 |
| MOL003971 | Threonin | 73.51568361 | 0.012819 |
| MOL000041 | PHA | 41.61788444 | 0.036077 |
| MOL004175 | NSC733507 | 17.31848908 | 0.7121 |
| MOL000042 | LPG | 87.69200501 | 0.0059892 |
| MOL004481 | (2S)-2-amino-3-[(2R)-2-amino-3-hydroxy-3-oxopropyl]disulfanylpropanoic acid | 73.59359187 | 0.048927 |
| MOL004495 | tigogenin | 14.08639946 | 0.80746 |
| MOL004502 | Monomethyl lithospermate | 2.665213473 | 0.74639 |
| MOL000476 | Physcion | 22.28640405 | 0.26659 |
| MOL004784 | Stenol | 12.65726871 | 0.11373 |
| MOL000485 | TMH | 46.24981501 | 0.052565 |
| MOL000050 | GLY | 48.73713548 | 0.0033178 |
| MOL000511 | ursolic acid | 16.77490232 | 0.75457 |
| MOL000052 | Gulutamine | 6.655918569 | 0.021387 |
| MOL000054 | L- | 47.64200167 | 0.031878 |
| MOL005423 | (-)-Epicedrol | 87.663741 | 0.12173 |
| MOL005448 | Leucinum | 72.91888177 | 0.014057 |
| MOL005449 | h-Met-h | 70.86633942 | 0.013018 |
| MOL000055 | L-Lysin | 29.33256218 | 0.018179 |
| MOL005508 | Glucosol | 15.86390133 | 0.73993 |
| MOL000056 | DTY | 57.55459471 | 0.046654 |
| MOL000569 | digallate | 61.84861803 | 0.25635 |
| MOL005928 | isoferulic acid | 50.82647607 | 0.058232 |
| MOL000006 | luteolin | 36.16262934 | 0.24552 |
| MOL000061 | Prolinum | 77.57468129 | 0.014161 |
| MOL000613 | 8-isopropylidene-1,5-dimethylcyclodeca-1,5-diene | 18.55581355 | 0.056941 |
| MOL006452 | 1,5-Dihydroxy-3-methylanthraquinone | 18.92474342 | 0.2095 |
| MOL000065 | ASI | 79.73701985 | 0.017132 |
| MOL000067 | L-Valin | 53.32980553 | 0.011914 |
| MOL000675 | oleic acid | 33.12836481 | 0.14243 |
| MOL000068 | L-Ile | 59.05137302 | 0.015008 |
| MOL006824 | α-amyrin | 39.51208978 | 0.76221 |
| MOL000069 | palmitic acid | 19.2965647 | 0.098573 |
| MOL007035 | (E)-3-(3-hydroxy-4,5-dimethoxy-phenyl)acrylic acid | 64.7068115 | 0.080604 |
| MOL007036 | 5,6-dihydroxy-7-isopropyl-1,1-dimethyl-2,3-dihydrophenanthren-4-one | 33.76525236 | 0.28585 |
| MOL007037 | 1,2-DT-Quinone | 19.95216373 | 0.36014 |
| MOL007038 | Dehydromiltirone | 24.57140555 | 0.25596 |
| MOL007039 | Henicosyl formate | 15.79283144 | 0.26296 |
| MOL007040 | 1-ketoisocryptotanshinone | 17.98746658 | 0.44153 |
| MOL007041 | 2-isopropyl-8-methylphenanthrene-3,4-dione | 40.86015408 | 0.22897 |
| MOL007042 | 3-epicorosolic,acid | 15.16386816 | 0.74003 |
| MOL007043 | 1-(3,4-dihydroxyphenyl)-2-hydroxyethanone | 56.78975564 | 0.041276 |
| MOL007044 | 3,7-dimethylocta-2,6-dien-1-yl formate | 50.94716238 | 0.033838 |
| MOL007045 | 3α-hydroxytanshinoneⅡa | 44.92933597 | 0.44272 |
| MOL007046 | 3beta-Hydroxytanshinone IIA | 20.52815887 | 0.44667 |
| MOL007047 | (1R,4R,5S)-1-isopropyl-4-methyl-4-bicyclo[3.1.0]hexanol | 94.91831064 | 0.053937 |
| MOL007048 | (E)-3-[2-(3,4-dihydroxyphenyl)-7-hydroxy-benzofuran-4-yl]acrylic acid | 48.24363244 | 0.31229 |
| MOL007049 | 4-methylenemiltirone | 34.34867589 | 0.22726 |
| MOL007050 | 2-(4-hydroxy-3-methoxyphenyl)-5-(3-hydroxypropyl)-7-methoxy-3-benzofurancarboxaldehyde | 62.78414726 | 0.39628 |
| MOL007051 | 6-o-syringyl-8-o-acetyl shanzhiside methyl ester | 46.6906586 | 0.71145 |
| MOL007052 | 6-o-syringyl-8-o-acetyl shanzhiside methyl ester_qt | 12.69784437 | 0.66951 |
| MOL007053 | 7-oxoroyleanone2 | 15.40848713 | 0.34075 |
| MOL007054 | (4bS,8aS,10S)-10-hydroxy-2-isopropyl-4b,8,8-trimethyl-5,6,7,8a,9,10-hexahydrophenanthrene-3,4-dione | 26.24581118 | 0.30689 |
| MOL007055 | 9-methyl lithospermate b | 3.013672117 | 0.39251 |
| MOL007056 | TNP00297 | 29.8895929 | 0.12929 |
| MOL001955 | Heriguard | 11.93273511 | 0.32642 |
| MOL007058 | formyltanshinone | 73.444622 | 0.41736 |
| MOL007059 | 3-beta-Hydroxymethyllenetanshiquinone | 32.16103376 | 0.40894 |
| MOL007060 | Lithospermic acid B | 3.013679057 | 0.40754 |
| MOL007061 | Methylenetanshinquinone | 37.07319368 | 0.36017 |
| MOL007062 | neo-przewaquinone a | 6.46794026 | 0.18537 |
| MOL007063 | przewalskin a | 37.10650066 | 0.64901 |
| MOL007064 | przewalskin b | 110.3240001 | 0.43809 |
| MOL007065 | przewalskin c | 20.33855059 | 0.35434 |
| MOL007066 | przewalskin d | 18.17397808 | 0.29194 |
| MOL007067 | Przewaquinone A | 21.9068533 | 0.45112 |
| MOL007068 | Przewaquinone B | 62.24005962 | 0.41374 |
| MOL007069 | przewaquinone c | 55.7416731 | 0.40408 |
| MOL007070 | (6S,7R)-6,7-dihydroxy-1,6-dimethyl-8,9-dihydro-7H-naphtho[8,7-g]benzofuran-10,11-dione | 41.31045706 | 0.453 |
| MOL007071 | przewaquinone f | 40.30788399 | 0.45925 |
| MOL007072 | (2S)-3-(3,4-dihydroxyphenyl)-2-hydroxypropanoic acid | 59.56484281 | 0.059824 |
| MOL007073 | saloilenone | 8.634615437 | 0.43477 |
| MOL007074 | salvianolic acid b | 3.013679057 | 0.4076 |
| MOL007075 | salvianolic acid n | 1.456233441 | 0.82841 |
| MOL007076 | Saprothoquinone | 18.26585476 | 0.20985 |
| MOL007077 | sclareol | 43.67068458 | 0.2058 |
| MOL010175 | Tannin | 7.894234081 | 0.033371 |
| MOL007079 | tanshinaldehyde | 52.4747043 | 0.45196 |
| MOL007080 | Tanshinol A | 21.31081211 | 0.41409 |
| MOL007081 | Danshenol B | 57.9508753 | 0.55764 |
| MOL007082 | Danshenol A | 56.96524899 | 0.52172 |
| MOL007083 | Z-8-Hexadecen-1-ol acetate | 34.99773997 | 0.14159 |
| MOL007084 | Aethiopinone | 21.02277296 | 0.21035 |
| MOL007085 | Salvilenone | 30.38365387 | 0.37639 |
| MOL007087 | carnosol | 14.95925619 | 0.4251 |
| MOL007088 | cryptotanshinone | 52.34196226 | 0.39555 |
| MOL007089 | Cyclotetradecane | 27.72170898 | 0.055376 |
| MOL007090 | dan-shexinkum a | 24.88398896 | 0.29549 |
| MOL007091 | dan-shexinkum b | 21.6705533 | 0.25955 |
| MOL007092 | dan-shexinkum c | 22.70962694 | 0.20855 |
| MOL007093 | dan-shexinkum d | 38.88302101 | 0.55453 |
| MOL007094 | danshenspiroketallactone | 50.43128103 | 0.3067 |
| MOL007095 | danshenspiroketallactoneii | 19.88565956 | 0.2925 |
| MOL007096 | daucosterol | 20.63193686 | 0.62522 |
| MOL007097 | dehydrouvaol | 16.42177132 | 0.76501 |
| MOL007098 | deoxyneocryptotanshinone | 49.40034705 | 0.28555 |
| MOL007099 | dihydroisotanshinoneⅠ | 20.91269483 | 0.35909 |
| MOL000071 | Istidina | 53.17530628 | 0.029475 |
| MOL007100 | dihydrotanshinlactone | 38.6847683 | 0.32227 |
| MOL007101 | dihydrotanshinoneⅠ | 45.04327919 | 0.36015 |
| MOL007102 | diisopro-penyl methyl vinyl cyclohexane2 | 48.65864377 | 0.068534 |
| MOL007103 | dimetbyl lithosper-mate b | 3.013665178 | 0.37883 |
| MOL007104 | dimethyllithospermate | 2.661221356 | 0.73096 |
| MOL007105 | epidanshenspiroketallactone | 68.27315929 | 0.30549 |
| MOL007106 | ethyl lithospermate | 26.36817061 | 0.82369 |
| MOL007107 | C09092 | 36.06948986 | 0.2474 |
| MOL007108 | isocryptotanshi-none | 54.98193246 | 0.39449 |
| MOL007109 | isosalvianolic acid c | 2.483933702 | 0.82961 |
| MOL007110 | isotanshinone iib | 21.06783944 | 0.44762 |
| MOL007111 | Isotanshinone II | 49.91602574 | 0.39674 |
| MOL007112 | Isotanshinone I | 29.71939745 | 0.36138 |
| MOL007113 | lithospermic acid | 2.67408372 | 0.75661 |
| MOL007115 | manool | 45.04431636 | 0.20208 |
| MOL007116 | methylrosmarinate | 1.374700213 | 0.37358 |
| MOL007117 | methyltanshinonate | 19.18668741 | 0.54837 |
| MOL007118 | microstegiol | 39.61229457 | 0.27734 |
| MOL007119 | miltionone Ⅰ | 49.68439433 | 0.32125 |
| MOL007120 | miltionone Ⅱ | 71.02970321 | 0.43711 |
| MOL007121 | miltipolone | 36.55611206 | 0.36803 |
| MOL007122 | Miltirone | 38.75698635 | 0.25418 |
| MOL007123 | miltirone Ⅱ | 44.95106648 | 0.23537 |
| MOL007124 | neocryptotanshinone ii | 39.46299114 | 0.23157 |
| MOL007125 | neocryptotanshinone | 52.48799701 | 0.32306 |
| MOL007126 | przewalskin | 25.177349 | 0.28238 |
| MOL007127 | 1-methyl-8,9-dihydro-7H-naphtho[5,6-g]benzofuran-6,10,11-trione | 34.72082213 | 0.36634 |
| MOL007128 | paramiltioic acid | 25.92029858 | 0.36631 |
| MOL007129 | potassium salvianolate d | 1.573946623 | 0.5005 |
| MOL007130 | prolithospermic acid | 64.37096207 | 0.31017 |
| MOL007131 | (2S,3S)-2-(3,4-dihydroxyphenyl)-7-hydroxy-4-[(E)-3-hydroxy-3-oxoprop-1-enyl]-2,3-dihydrobenzofuran-3-carboxylic acid | 2.622333965 | 0.40965 |
| MOL007132 | (2R)-3-(3,4-dihydroxyphenyl)-2-[(Z)-3-(3,4-dihydroxyphenyl)acryloyl]oxy-propionic acid | 109.3805241 | 0.35119 |
| MOL007133 | salviacoccin | 13.34934632 | 0.63018 |
| MOL007134 | danshensu | 36.91475582 | 0.059833 |
| MOL007135 | salvianic acid c | 1.450293832 | 0.37328 |
| MOL007136 | salvianolic acid a | 2.956732261 | 0.70245 |
| MOL007137 | salvianolic acid c | 2.500662031 | 0.80524 |
| MOL007138 | salvianolic acid d | 1.573946623 | 0.50059 |
| MOL007139 | salvianolic acid e | 3.013720692 | 0.38928 |
| MOL007140 | (Z)-3-[2-[(E)-2-(3,4-dihydroxyphenyl)vinyl]-3,4-dihydroxy-phenyl]acrylic acid | 88.53602101 | 0.25869 |
| MOL007141 | salvianolic acid g | 45.56485578 | 0.60602 |
| MOL007142 | salvianolic acid j | 43.37604991 | 0.72497 |
| MOL007143 | salvilenone Ⅰ | 32.43470856 | 0.22895 |
| MOL007144 | salviol | 24.31364101 | 0.27766 |
| MOL007145 | salviolone | 31.72415039 | 0.23568 |
| MOL007146 | salvipisone | 25.03340929 | 0.2376 |
| MOL007147 | methyl (1S,4aS,5R,7S,7aS)-5,7-dihydroxy-7-methyl-1-[(2S,3R,4S,5S,6R)-3,4,5-trihydroxy-6-(hydroxymethyl)oxan-2-yl]oxy-4a,5,6,7a-tetrahydro-1H-cyclopenta[d]pyran-4-carboxylate | 6.460304671 | 0.47445 |
| MOL007148 | shanzhiside methyl ester_qt | 109.774129 | 0.11597 |
| MOL007149 | NSC 122421 | 34.49292309 | 0.27645 |
| MOL007150 | (6S)-6-hydroxy-1-methyl-6-methylol-8,9-dihydro-7H-naphtho[8,7-g]benzofuran-10,11-quinone | 75.38587847 | 0.4551 |
| MOL007151 | Tanshindiol B | 42.66581049 | 0.45303 |
| MOL007152 | Przewaquinone E | 42.85485204 | 0.45301 |
| MOL007153 | Tanshilactone | 25.10556502 | 0.32443 |
| MOL007154 | tanshinone iia | 49.88730004 | 0.39781 |
| MOL007155 | (6S)-6-(hydroxymethyl)-1,6-dimethyl-8,9-dihydro-7H-naphtho[8,7-g]benzofuran-10,11-dione | 65.25893771 | 0.44871 |
| MOL007156 | tanshinone Ⅵ | 45.63730602 | 0.29549 |
| MOL007157 | tanshinone i | 29.26509916 | 0.36245 |
| MOL007158 | Spirostan-3-ol, (3beta,5alpha,25S)- | 13.16163977 | 0.8073 |
| MOL007159 | uvaol | 17.70801984 | 0.76189 |
| MOL007160 | β-cadinol | 37.76330533 | 0.093271 |
| MOL000008 | apigenin | 23.06216102 | 0.21306 |
| MOL000860 | stearic acid | 17.82542938 | 0.14086 |
| MOL000865 | hexadecane | 12.31600907 | 0.060928 |
| MOL000869 | Henicosane | 8.412905294 | 0.15364 |
| MOL000009 | luteolin-7-o-glucoside | 7.292677359 | 0.77998 |
| MOL000922 | (R)-p-Menth-1-en-4-ol | 32.15585044 | 0.032284 |
| MOL003127 | Germacrene D | 19.22250577 | 0.057074 |
| MOL000932 | alpha-Farnesene | 21.70351121 | 0.047239 |
| MOL000942 | (1R,4S,4aR,8aR)-4-isopropyl-1,6-dimethyl-3,4,4a,7,8,8a-hexahydro-2H-naphthalen-1-ol | 31.67305829 | 0.093126 |

Supplementary Table 2. Candidate components of Danshen.

| Mol ID | Molecule Name | OB (%) | DL |
| --- | --- | --- | --- |
| MOL001601 | 1,2,5,6-tetrahydroTanshinone | 38.74538672 | 0.35791 |
| MOL001659 | Poriferasterol | 43.82985158 | 0.75596 |
| MOL001771 | poriferast-5-en-3beta-ol | 36.91390583 | 0.75034 |
| MOL001942 | isoimperatorin | 45.46424674 | 0.22524 |
| MOL002222 | sugiol | 36.11353486 | 0.27648 |
| MOL002651 | DehydroTanshinone II A | 43.76228599 | 0.40019 |
| MOL002776 | Baicalin | 40.12360996 | 0.75264 |
| MOL000569 | digallate | 61.84861803 | 0.25635 |
| MOL000006 | luteolin | 36.16262934 | 0.24552 |
| MOL007036 | 5,6-dihydroxy-7-isopropyl-1,1-dimethyl-2,3-dihydrophenanthren-4-one | 33.76525236 | 0.28585 |
| MOL007041 | 2-isopropyl-8-methylphenanthrene-3,4-dione | 40.86015408 | 0.22897 |
| MOL007045 | 3α-hydroxyTanshinoneⅡa | 44.92933597 | 0.44272 |
| MOL007048 | (E)-3-[2-(3,4-dihydroxyphenyl)-7-hydroxy-benzofuran-4-yl]acrylic acid | 48.24363244 | 0.31229 |
| MOL007049 | 4-methylenemiltirone | 34.34867589 | 0.22726 |
| MOL007050 | 2-(4-hydroxy-3-methoxyphenyl)-5-(3-hydroxypropyl)-7-methoxy-3-benzofurancarboxaldehyde | 62.78414726 | 0.39628 |
| MOL007058 | formylTanshinone | 73.444622 | 0.41736 |
| MOL007059 | 3-beta-Hydroxymethyllenetanshiquinone | 32.16103376 | 0.40894 |
| MOL007061 | Methylenetanshinquinone | 37.07319368 | 0.36017 |
| MOL007063 | przewalskin a | 37.10650066 | 0.64901 |
| MOL007064 | przewalskin b | 110.3240001 | 0.43809 |
| MOL007068 | Przewaquinone B | 62.24005962 | 0.41374 |
| MOL007069 | przewaquinone c | 55.7416731 | 0.40408 |
| MOL007070 | (6S,7R)-6,7-dihydroxy-1,6-dimethyl-8,9-dihydro-7H-naphtho[8,7-g]benzofuran-10,11-dione | 41.31045706 | 0.453 |
| MOL007071 | przewaquinone f | 40.30788399 | 0.45925 |
| MOL007077 | sclareol | 43.67068458 | 0.2058 |
| MOL007079 | tanshinaldehyde | 52.4747043 | 0.45196 |
| MOL007081 | Danshenol B | 57.9508753 | 0.55764 |
| MOL007082 | Danshenol A | 56.96524899 | 0.52172 |
| MOL007085 | Salvilenone | 30.38365387 | 0.37639 |
| MOL007088 | cryptoTanshinone | 52.34196226 | 0.39555 |
| MOL007093 | dan-shexinkum d | 38.88302101 | 0.55453 |
| MOL007094 | danshenspiroketallactone | 50.43128103 | 0.3067 |
| MOL007098 | deoxyneocryptoTanshinone | 49.40034705 | 0.28555 |
| MOL007100 | dihydrotanshinlactone | 38.6847683 | 0.32227 |
| MOL007101 | dihydroTanshinoneⅠ | 45.04327919 | 0.36015 |
| MOL007105 | epidanshenspiroketallactone | 68.27315929 | 0.30549 |
| MOL007107 | C09092 | 36.06948986 | 0.2474 |
| MOL007108 | isocryptotanshi-none | 54.98193246 | 0.39449 |
| MOL007111 | IsoTanshinone II | 49.91602574 | 0.39674 |
| MOL007115 | manool | 45.04431636 | 0.20208 |
| MOL007119 | miltionone Ⅰ | 49.68439433 | 0.32125 |
| MOL007120 | miltionone Ⅱ | 71.02970321 | 0.43711 |
| MOL007121 | miltipolone | 36.55611206 | 0.36803 |
| MOL007122 | Miltirone | 38.75698635 | 0.25418 |
| MOL007124 | neocryptoTanshinone ii | 39.46299114 | 0.23157 |
| MOL007125 | neocryptoTanshinone | 52.48799701 | 0.32306 |
| MOL007127 | 1-methyl-8,9-dihydro-7H-naphtho[5,6-g]benzofuran-6,10,11-trione | 34.72082213 | 0.36634 |
| MOL007130 | prolithospermic acid | 64.37096207 | 0.31017 |
| MOL007132 | (2R)-3-(3,4-dihydroxyphenyl)-2-[(Z)-3-(3,4-dihydroxyphenyl)acryloyl]oxy-propionic acid | 109.3805241 | 0.35119 |
| MOL007141 | salvianolic acid g | 45.56485578 | 0.60602 |
| MOL007142 | salvianolic acid j | 43.37604991 | 0.72497 |
| MOL007143 | salvilenone Ⅰ | 32.43470856 | 0.22895 |
| MOL007145 | salviolone | 31.72415039 | 0.23568 |
| MOL007150 | (6S)-6-hydroxy-1-methyl-6-methylol-8,9-dihydro-7H-naphtho[8,7-g]benzofuran-10,11-quinone | 75.38587847 | 0.4551 |
| MOL007151 | Tanshindiol B | 42.66581049 | 0.45303 |
| MOL007152 | Przewaquinone E | 42.85485204 | 0.45301 |
| MOL007154 | Tanshinone iia | 49.88730004 | 0.39781 |
| MOL007155 | (6S)-6-(hydroxymethyl)-1,6-dimethyl-8,9-dihydro-7H-naphtho[8,7-g]benzofuran-10,11-dione | 65.25893771 | 0.44871 |
| MOL007156 | Tanshinone Ⅵ | 45.63730602 | 0.29549 |

Supplementary table 3. Potential targets and corresponding symbols of Danshen candidate components

| MOL_ID | Mol Name | | Target name | Symbol |
| --- | --- | --- | --- | --- |
| MOL000006 | luteolin | Prostaglandin G/H synthase 1 | | PTGS1 |
| MOL000006 | luteolin | Androgen receptor | | PKN1 |
| MOL000006 | luteolin | Prostaglandin G/H synthase 2 | | PTGES3 |
| MOL000006 | luteolin | Heat shock protein HSP 90 | | HSPB1 |
| MOL000006 | luteolin | Trypsin-1 | | PRSS1 |
| MOL000006 | luteolin | Nuclear receptor coactivator 2 | | BUD23 |
| MOL000006 | luteolin | mRNA of PKA Catalytic Subunit C-alpha | | AKAP13 |
| MOL000006 | luteolin | Dipeptidyl peptidase IV | | DPP4 |
| MOL000006 | luteolin | Phosphatidylinositol-4,5-bisphosphate 3-kinase catalytic subunit, gamma isoform | | PIK3CG |
| MOL000006 | luteolin | Transcription factor p65 | | CDK9 |
| MOL000006 | luteolin | Epidermal growth factor receptor | | PDGFRB |
| MOL000006 | luteolin | RAC-alpha serine/threonine-protein kinase | | BCR |
| MOL000006 | luteolin | Vascular endothelial growth factor A | | VEGFA |
| MOL000006 | luteolin | G1/S-specific cyclin-D1 | | CCND3 |
| MOL000006 | luteolin | Bcl-2-like protein 1 | | BAK1 |
| MOL000006 | luteolin | Cyclin-dependent kinase inhibitor 1 | | CDK9 |
| MOL000006 | luteolin | Caspase-9 | | CASP9 |
| MOL000006 | luteolin | 72 kDa type IV collagenase | | MMP2 |
| MOL000006 | luteolin | Matrix metalloproteinase-9 | | MMP9 |
| MOL000006 | luteolin | Mitogen-activated protein kinase 1 | | MAPK14 |
| MOL000006 | luteolin | Interleukin-10 | | IL26 |
| MOL000006 | luteolin | Retinoblastoma-associated protein | | RBL1 |
| MOL000006 | luteolin | Cell division protein kinase 4 | | PKN2 |
| MOL000006 | luteolin | Tumor necrosis factor | | TNF |
| MOL000006 | luteolin | Transcription factor AP-1 | | ESR1 |
| MOL000006 | luteolin | Interleukin-6 | | IL6R |
| MOL000006 | luteolin | Caspase-3 | | HTT |
| MOL000006 | luteolin | Cellular tumor antigen p53 | | TP53 |
| MOL000006 | luteolin | NF-kappa-B inhibitor alpha | | ARRB2 |
| MOL000006 | luteolin | Xanthine dehydrogenase/oxidase | | XDH |
| MOL000006 | luteolin | DNA topoisomerase 1 | | TOP1 |
| MOL000006 | luteolin | E3 ubiquitin-protein ligase Mdm2 | | CUL4A |
| MOL000006 | luteolin | Amyloid beta A4 protein | | APP |
| MOL000006 | luteolin | Interstitial collagenase | | MMP1 |
| MOL000006 | luteolin | Proliferating cell nuclear antigen | | PCNA |
| MOL000006 | luteolin | Receptor tyrosine-protein kinase erbB-2 | | ERBB4 |
| MOL000006 | luteolin | Peroxisome proliferator-activated receptor gamma | | PPARA |
| MOL000006 | luteolin | Heme oxygenase 1 | | TDO2 |
| MOL000006 | luteolin | Caspase-7 | | RAD21 |
| MOL000006 | luteolin | Intercellular adhesion molecule 1 | | CTNNA1 |
| MOL000006 | luteolin | Induced myeloid leukemia cell differentiation protein Mcl-1 | | MCL1 |
| MOL000006 | luteolin | Baculoviral IAP repeat-containing protein 5 | | BIRC8 |
| MOL000006 | luteolin | Interleukin-2 | | IL2 |
| MOL000006 | luteolin | G2/mitotic-specific cyclin-B1 | | CCNB2 |
| MOL000006 | luteolin | Tyrosinase | | TYR |
| MOL000006 | luteolin | Interferon gamma | | SP100 |
| MOL000006 | luteolin | Interleukin-4 | | IL4 |
| MOL000006 | luteolin | DNA topoisomerase 2-alpha | | TOP2A |
| MOL000006 | luteolin | Glutathione S-transferase P | | GSTP1 |
| MOL000006 | luteolin | Baculoviral IAP repeat-containing protein 4 | | BIRC5 |
| MOL000006 | luteolin | Solute carrier family 2, facilitated glucose transporter member 4 | | SLC2A14 |
| MOL000006 | luteolin | Insulin receptor | | INSR |
| MOL000006 | luteolin | CD40 ligand | | CD40LG |
| MOL000006 | luteolin | Prostaglandin E synthase | | PTGES3 |
| MOL000006 | luteolin | Kinetochore protein Nuf2 | | CENPE |
| MOL000006 | luteolin | Adenylate cyclase type 2 | | ADCY2 |
| MOL000006 | luteolin | Hepatocyte growth factor receptor | | FGFR4 |
| MOL000569 | digallate | Prostaglandin G/H synthase 2 | | PTGES3 |
| MOL000569 | digallate | Heat shock protein HSP 90 | | HSPB1 |
| MOL000569 | digallate | Aldose reductase | | AKR1B1 |
| MOL001601 | 1,2,5,6-tetrahydrotanshinone | Prostaglandin G/H synthase 1 | | PTGS1 |
| MOL001601 | 1,2,5,6-tetrahydrotanshinone | Muscarinic acetylcholine receptor M3 | | CHRM3 |
| MOL001601 | 1,2,5,6-tetrahydrotanshinone | Muscarinic acetylcholine receptor M1 | | CHRM1 |
| MOL001601 | 1,2,5,6-tetrahydrotanshinone | Sodium channel protein type 5 subunit alpha | | SCNN1B |
| MOL001601 | 1,2,5,6-tetrahydrotanshinone | Muscarinic acetylcholine receptor M5 | | CHRM5 |
| MOL001601 | 1,2,5,6-tetrahydrotanshinone | Prostaglandin G/H synthase 2 | | PTGES3 |
| MOL001601 | 1,2,5,6-tetrahydrotanshinone | 5-hydroxytryptamine receptor 3A | | HTR3A |
| MOL001601 | 1,2,5,6-tetrahydrotanshinone | Carbonic anhydrase II | | CA5A |
| MOL001601 | 1,2,5,6-tetrahydrotanshinone | Muscarinic acetylcholine receptor M4 | | CHRM4 |
| MOL001601 | 1,2,5,6-tetrahydrotanshinone | Retinoic acid receptor RXR-alpha | | RXRA |
| MOL001601 | 1,2,5,6-tetrahydrotanshinone | Delta-type opioid receptor | | OPRD1 |
| MOL001601 | 1,2,5,6-tetrahydrotanshinone | CGMP-inhibited 3',5'-cyclic phosphodiesterase A | | PDE3A |
| MOL001601 | 1,2,5,6-tetrahydrotanshinone | Alpha-1A adrenergic receptor | | ADRA1A |
| MOL001601 | 1,2,5,6-tetrahydrotanshinone | Muscarinic acetylcholine receptor M2 | | CHRM2 |
| MOL001601 | 1,2,5,6-tetrahydrotanshinone | Alpha-1B adrenergic receptor | | ADRA1B |
| MOL001601 | 1,2,5,6-tetrahydrotanshinone | Sodium-dependent dopamine transporter | | SLC6A3 |
| MOL001601 | 1,2,5,6-tetrahydrotanshinone | Beta-2 adrenergic receptor | | GRK2 |
| MOL001601 | 1,2,5,6-tetrahydrotanshinone | Alpha-1D adrenergic receptor | | ADRA1D |
| MOL001601 | 1,2,5,6-tetrahydrotanshinone | DNA topoisomerase II | | DHX9 |
| MOL001601 | 1,2,5,6-tetrahydrotanshinone | Mu-type opioid receptor | | OPRM1 |
| MOL001601 | 1,2,5,6-tetrahydrotanshinone | Gamma-aminobutyric acid receptor subunit alpha-1 | | GABRA1 |
| MOL001601 | 1,2,5,6-tetrahydrotanshinone | Heat shock protein HSP 90 | | HSPB1 |
| MOL001601 | 1,2,5,6-tetrahydrotanshinone | Neuronal acetylcholine receptor protein, alpha-7 chain | | CHRNA6 |
| MOL001601 | 1,2,5,6-tetrahydrotanshinone | Nuclear receptor coactivator 2 | | BUD23 |
| MOL001601 | 1,2,5,6-tetrahydrotanshinone | Nuclear receptor coactivator 1 | | BUD23 |
| MOL001601 | 1,2,5,6-tetrahydrotanshinone | Dopamine D1 receptor | | DRD1 |
| MOL001601 | 1,2,5,6-tetrahydrotanshinone | 5-hydroxytryptamine 2A receptor | | HTR2A |
| MOL001601 | 1,2,5,6-tetrahydrotanshinone | Sodium-dependent serotonin transporter | | SLC6A4 |
| MOL001601 | 1,2,5,6-tetrahydrotanshinone | Ig gamma-1 chain C region | | IGHG1 |
| MOL001659 | Poriferasterol | Progesterone receptor | | NR3C2 |
| MOL001659 | Poriferasterol | Mineralocorticoid receptor | | BUD23 |
| MOL001771 | poriferast-5-en-3beta-ol | Progesterone receptor | | NR3C2 |
| MOL001771 | poriferast-5-en-3beta-ol | Nuclear receptor coactivator 2 | | BUD23 |
| MOL001942 | isoimperatorin | Prostaglandin G/H synthase 2 | | PTGES3 |
| MOL002222 | sugiol | Muscarinic acetylcholine receptor M3 | | CHRM3 |
| MOL002222 | sugiol | Muscarinic acetylcholine receptor M1 | | CHRM1 |
| MOL002222 | sugiol | Sodium channel protein type 5 subunit alpha | | SCNN1B |
| MOL002222 | sugiol | Muscarinic acetylcholine receptor M5 | | CHRM5 |
| MOL002222 | sugiol | Prostaglandin G/H synthase 2 | | PTGES3 |
| MOL002222 | sugiol | Carbonic anhydrase II | | CA5A |
| MOL002222 | sugiol | Muscarinic acetylcholine receptor M4 | | CHRM4 |
| MOL002222 | sugiol | Delta-type opioid receptor | | OPRD1 |
| MOL002222 | sugiol | Acetylcholinesterase | | ACHE |
| MOL002222 | sugiol | Alpha-1A adrenergic receptor | | ADRA1A |
| MOL002222 | sugiol | Muscarinic acetylcholine receptor M2 | | CHRM2 |
| MOL002222 | sugiol | Alpha-1B adrenergic receptor | | ADRA1B |
| MOL002222 | sugiol | Beta-2 adrenergic receptor | | GRK2 |
| MOL002222 | sugiol | Alpha-1D adrenergic receptor | | ADRA1D |
| MOL002222 | sugiol | D(2) dopamine receptor | | SNCA |
| MOL002222 | sugiol | Mu-type opioid receptor | | OPRM1 |
| MOL002222 | sugiol | Neuronal acetylcholine receptor protein, alpha-7 chain | | CHRNA6 |
| MOL002651 | Dehydrotanshinone II A | Dopamine D1 receptor | | DRD1 |
| MOL002651 | Dehydrotanshinone II A | Muscarinic acetylcholine receptor M3 | | CHRM3 |
| MOL002651 | Dehydrotanshinone II A | Thrombin | | CXCL8 |
| MOL002651 | Dehydrotanshinone II A | Muscarinic acetylcholine receptor M1 | | CHRM1 |
| MOL002651 | Dehydrotanshinone II A | Estrogen receptor | | CTH |
| MOL002651 | Dehydrotanshinone II A | Androgen receptor | | PKN1 |
| MOL002651 | Dehydrotanshinone II A | Sodium channel protein type 5 subunit alpha | | SCNN1B |
| MOL002651 | Dehydrotanshinone II A | Peroxisome proliferator activated receptor gamma | | PPARA |
| MOL002651 | Dehydrotanshinone II A | Muscarinic acetylcholine receptor M5 | | CHRM5 |
| MOL002651 | Dehydrotanshinone II A | Prostaglandin G/H synthase 2 | | PTGES3 |
| MOL002651 | Dehydrotanshinone II A | Muscarinic acetylcholine receptor M4 | | CHRM4 |
| MOL002651 | Dehydrotanshinone II A | Delta-type opioid receptor | | OPRD1 |
| MOL002651 | Dehydrotanshinone II A | Acetylcholinesterase | | ACHE |
| MOL002651 | Dehydrotanshinone II A | 5-hydroxytryptamine 2A receptor | | HTR2A |
| MOL002651 | Dehydrotanshinone II A | Alpha-1A adrenergic receptor | | ADRA1A |
| MOL002651 | Dehydrotanshinone II A | Beta-2 adrenergic receptor | | GRK2 |
| MOL002651 | Dehydrotanshinone II A | Mu-type opioid receptor | | OPRM1 |
| MOL002651 | Dehydrotanshinone II A | Gamma-aminobutyric acid receptor subunit alpha-1 | | GABRA1 |
| MOL002651 | Dehydrotanshinone II A | Dipeptidyl peptidase IV | | DPP4 |
| MOL002651 | Dehydrotanshinone II A | Neuronal acetylcholine receptor protein, alpha-7 chain | | CHRNA6 |
| MOL002651 | Dehydrotanshinone II A | Nuclear receptor coactivator 1 | | BUD23 |
| MOL002776 | Baicalin | Coagulation factor Xa | | F2RL1 |
| MOL002776 | Baicalin | mRNA of Protein-tyrosine phosphatase, non-receptor type 1 | | PTPN22 |
| MOL007036 | 5,6-dihydroxy-7-isopropyl-1,1-dimethyl-2,3-dihydrophenanthren-4-one | Prostaglandin G/H synthase 1 | | PTGS1 |
| MOL007036 | 5,6-dihydroxy-7-isopropyl-1,1-dimethyl-2,3-dihydrophenanthren-4-one | Muscarinic acetylcholine receptor M3 | | CHRM3 |
| MOL007036 | 5,6-dihydroxy-7-isopropyl-1,1-dimethyl-2,3-dihydrophenanthren-4-one | Muscarinic acetylcholine receptor M1 | | CHRM1 |
| MOL007036 | 5,6-dihydroxy-7-isopropyl-1,1-dimethyl-2,3-dihydrophenanthren-4-one | Sodium channel protein type 5 subunit alpha | | SCNN1B |
| MOL007036 | 5,6-dihydroxy-7-isopropyl-1,1-dimethyl-2,3-dihydrophenanthren-4-one | Prostaglandin G/H synthase 2 | | PTGES3 |
| MOL007036 | 5,6-dihydroxy-7-isopropyl-1,1-dimethyl-2,3-dihydrophenanthren-4-one | Carbonic anhydrase II | | CA5A |
| MOL007036 | 5,6-dihydroxy-7-isopropyl-1,1-dimethyl-2,3-dihydrophenanthren-4-one | Retinoic acid receptor RXR-alpha | | RXRA |
| MOL007036 | 5,6-dihydroxy-7-isopropyl-1,1-dimethyl-2,3-dihydrophenanthren-4-one | Acetylcholinesterase | | ACHE |
| MOL007036 | 5,6-dihydroxy-7-isopropyl-1,1-dimethyl-2,3-dihydrophenanthren-4-one | Alpha-1A adrenergic receptor | | ADRA1A |
| MOL007036 | 5,6-dihydroxy-7-isopropyl-1,1-dimethyl-2,3-dihydrophenanthren-4-one | Alpha-1B adrenergic receptor | | ADRA1B |
| MOL007036 | 5,6-dihydroxy-7-isopropyl-1,1-dimethyl-2,3-dihydrophenanthren-4-one | Beta-2 adrenergic receptor | | GRK2 |
| MOL007036 | 5,6-dihydroxy-7-isopropyl-1,1-dimethyl-2,3-dihydrophenanthren-4-one | DNA topoisomerase II | | DHX9 |
| MOL007036 | 5,6-dihydroxy-7-isopropyl-1,1-dimethyl-2,3-dihydrophenanthren-4-one | Mu-type opioid receptor | | OPRM1 |
| MOL007036 | 5,6-dihydroxy-7-isopropyl-1,1-dimethyl-2,3-dihydrophenanthren-4-one | Ig gamma-1 chain C region | | IGHG1 |
| MOL007036 | 5,6-dihydroxy-7-isopropyl-1,1-dimethyl-2,3-dihydrophenanthren-4-one | Nuclear receptor coactivator 2 | | BUD23 |
| MOL007036 | 5,6-dihydroxy-7-isopropyl-1,1-dimethyl-2,3-dihydrophenanthren-4-one | Nuclear receptor coactivator 1 | | BUD23 |
| MOL007036 | 5,6-dihydroxy-7-isopropyl-1,1-dimethyl-2,3-dihydrophenanthren-4-one | Calmodulin | | IQCB1 |
| MOL007041 | 2-isopropyl-8-methylphenanthrene-3,4-dione | Prostaglandin G/H synthase 1 | | PTGS1 |
| MOL007041 | 2-isopropyl-8-methylphenanthrene-3,4-dione | Dopamine D1 receptor | | DRD1 |
| MOL007041 | 2-isopropyl-8-methylphenanthrene-3,4-dione | Muscarinic acetylcholine receptor M3 | | CHRM3 |
| MOL007041 | 2-isopropyl-8-methylphenanthrene-3,4-dione | Muscarinic acetylcholine receptor M1 | | CHRM1 |
| MOL007041 | 2-isopropyl-8-methylphenanthrene-3,4-dione | Estrogen receptor | | CTH |
| MOL007041 | 2-isopropyl-8-methylphenanthrene-3,4-dione | Androgen receptor | | PKN1 |
| MOL007041 | 2-isopropyl-8-methylphenanthrene-3,4-dione | Sodium channel protein type 5 subunit alpha | | SCNN1B |
| MOL007041 | 2-isopropyl-8-methylphenanthrene-3,4-dione | Peroxisome proliferator activated receptor gamma | | PPARA |
| MOL007041 | 2-isopropyl-8-methylphenanthrene-3,4-dione | Muscarinic acetylcholine receptor M5 | | CHRM5 |
| MOL007041 | 2-isopropyl-8-methylphenanthrene-3,4-dione | Prostaglandin G/H synthase 2 | | PTGES3 |
| MOL007041 | 2-isopropyl-8-methylphenanthrene-3,4-dione | Nitric-oxide synthase, endothelial | | KDR |
| MOL007041 | 2-isopropyl-8-methylphenanthrene-3,4-dione | 5-hydroxytryptamine receptor 3A | | HTR3A |
| MOL007041 | 2-isopropyl-8-methylphenanthrene-3,4-dione | Muscarinic acetylcholine receptor M4 | | CHRM4 |
| MOL007041 | 2-isopropyl-8-methylphenanthrene-3,4-dione | Retinoic acid receptor RXR-alpha | | RXRA |
| MOL007041 | 2-isopropyl-8-methylphenanthrene-3,4-dione | 5-hydroxytryptamine 2A receptor | | HTR2A |
| MOL007041 | 2-isopropyl-8-methylphenanthrene-3,4-dione | Alpha-1A adrenergic receptor | | ADRA1A |
| MOL007041 | 2-isopropyl-8-methylphenanthrene-3,4-dione | Muscarinic acetylcholine receptor M2 | | CHRM2 |
| MOL007041 | 2-isopropyl-8-methylphenanthrene-3,4-dione | Alpha-1B adrenergic receptor | | ADRA1B |
| MOL007041 | 2-isopropyl-8-methylphenanthrene-3,4-dione | Sodium-dependent dopamine transporter | | SLC6A3 |
| MOL007041 | 2-isopropyl-8-methylphenanthrene-3,4-dione | Beta-2 adrenergic receptor | | GRK2 |
| MOL007041 | 2-isopropyl-8-methylphenanthrene-3,4-dione | Alpha-1D adrenergic receptor | | ADRA1D |
| MOL007041 | 2-isopropyl-8-methylphenanthrene-3,4-dione | Sodium-dependent serotonin transporter | | SLC6A4 |
| MOL007041 | 2-isopropyl-8-methylphenanthrene-3,4-dione | Mu-type opioid receptor | | OPRM1 |
| MOL007041 | 2-isopropyl-8-methylphenanthrene-3,4-dione | Gamma-aminobutyric acid receptor subunit alpha-1 | | GABRA1 |
| MOL007041 | 2-isopropyl-8-methylphenanthrene-3,4-dione | Cell division protein kinase 2 | | CDK2 |
| MOL007041 | 2-isopropyl-8-methylphenanthrene-3,4-dione | Phosphatidylinositol-4,5-bisphosphate 3-kinase catalytic subunit, gamma isoform | | PIK3CG |
| MOL007041 | 2-isopropyl-8-methylphenanthrene-3,4-dione | Neuronal acetylcholine receptor protein, alpha-7 chain | | CHRNA6 |
| MOL007041 | 2-isopropyl-8-methylphenanthrene-3,4-dione | mRNA of PKA Catalytic Subunit C-alpha | | AKAP13 |
| MOL007041 | 2-isopropyl-8-methylphenanthrene-3,4-dione | Ig gamma-1 chain C region | | IGHG1 |
| MOL007041 | 2-isopropyl-8-methylphenanthrene-3,4-dione | Proto-oncogene serine/threonine-protein kinase Pim-1 | | PIM1 |
| MOL007041 | 2-isopropyl-8-methylphenanthrene-3,4-dione | Cyclin-A2 | | CCNA2 |
| MOL007041 | 2-isopropyl-8-methylphenanthrene-3,4-dione | Nuclear receptor coactivator 2 | | BUD23 |
| MOL007041 | 2-isopropyl-8-methylphenanthrene-3,4-dione | Calmodulin | | IQCB1 |
| MOL007045 | 3α-hydroxytanshinoneⅡa | Thrombin | | CXCL8 |
| MOL007045 | 3α-hydroxytanshinoneⅡa | Muscarinic acetylcholine receptor M1 | | CHRM1 |
| MOL007045 | 3α-hydroxytanshinoneⅡa | Sodium channel protein type 5 subunit alpha | | SCNN1B |
| MOL007045 | 3α-hydroxytanshinoneⅡa | Muscarinic acetylcholine receptor M5 | | CHRM5 |
| MOL007045 | 3α-hydroxytanshinoneⅡa | Prostaglandin G/H synthase 2 | | PTGES3 |
| MOL007045 | 3α-hydroxytanshinoneⅡa | Delta-type opioid receptor | | OPRD1 |
| MOL007045 | 3α-hydroxytanshinoneⅡa | Acetylcholinesterase | | ACHE |
| MOL007045 | 3α-hydroxytanshinoneⅡa | Beta-2 adrenergic receptor | | GRK2 |
| MOL007045 | 3α-hydroxytanshinoneⅡa | Mu-type opioid receptor | | OPRM1 |
| MOL007045 | 3α-hydroxytanshinoneⅡa | Dipeptidyl peptidase IV | | DPP4 |
| MOL007045 | 3α-hydroxytanshinoneⅡa | Neuronal acetylcholine receptor protein, alpha-7 chain | | CHRNA6 |
| MOL007045 | 3α-hydroxytanshinoneⅡa | Trypsin-1 | | PRSS1 |
| MOL007045 | 3α-hydroxytanshinoneⅡa | Nuclear receptor coactivator 1 | | BUD23 |
| MOL007048 | (E)-3-[2-(3,4-dihydroxyphenyl)-7-hydroxy-benzofuran-4-yl]acrylic acid | Prostaglandin G/H synthase 2 | | PTGES3 |
| MOL007048 | (E)-3-[2-(3,4-dihydroxyphenyl)-7-hydroxy-benzofuran-4-yl]acrylic acid | mRNA of Protein-tyrosine phosphatase, non-receptor type 1 | | PTPN22 |
| MOL007048 | (E)-3-[2-(3,4-dihydroxyphenyl)-7-hydroxy-benzofuran-4-yl]acrylic acid | Heat shock protein HSP 90 | | HSPB1 |
| MOL007049 | 4-methylenemiltirone | Prostaglandin G/H synthase 1 | | PTGS1 |
| MOL007049 | 4-methylenemiltirone | Dopamine D1 receptor | | DRD1 |
| MOL007049 | 4-methylenemiltirone | Muscarinic acetylcholine receptor M3 | | CHRM3 |
| MOL007049 | 4-methylenemiltirone | Muscarinic acetylcholine receptor M1 | | CHRM1 |
| MOL007049 | 4-methylenemiltirone | Estrogen receptor | | CTH |
| MOL007049 | 4-methylenemiltirone | Androgen receptor | | PKN1 |
| MOL007049 | 4-methylenemiltirone | Sodium channel protein type 5 subunit alpha | | SCNN1B |
| MOL007049 | 4-methylenemiltirone | Peroxisome proliferator activated receptor gamma | | PPARA |
| MOL007049 | 4-methylenemiltirone | Muscarinic acetylcholine receptor M5 | | CHRM5 |
| MOL007049 | 4-methylenemiltirone | Prostaglandin G/H synthase 2 | | PTGES3 |
| MOL007049 | 4-methylenemiltirone | Nitric-oxide synthase, endothelial | | KDR |
| MOL007049 | 4-methylenemiltirone | Alpha-2A adrenergic receptor | | ADRA2A |
| MOL007049 | 4-methylenemiltirone | Carbonic anhydrase II | | CA5A |
| MOL007049 | 4-methylenemiltirone | Alpha-2C adrenergic receptor | | ADRA2C |
| MOL007049 | 4-methylenemiltirone | Muscarinic acetylcholine receptor M4 | | CHRM4 |
| MOL007049 | 4-methylenemiltirone | Retinoic acid receptor RXR-alpha | | RXRA |
| MOL007049 | 4-methylenemiltirone | Delta-type opioid receptor | | OPRD1 |
| MOL007049 | 4-methylenemiltirone | 5-hydroxytryptamine 2A receptor | | HTR2A |
| MOL007049 | 4-methylenemiltirone | Alpha-1A adrenergic receptor | | ADRA1A |
| MOL007049 | 4-methylenemiltirone | Muscarinic acetylcholine receptor M2 | | CHRM2 |
| MOL007049 | 4-methylenemiltirone | Alpha-1B adrenergic receptor | | ADRA1B |
| MOL007049 | 4-methylenemiltirone | Sodium-dependent dopamine transporter | | SLC6A3 |
| MOL007049 | 4-methylenemiltirone | Beta-2 adrenergic receptor | | GRK2 |
| MOL007049 | 4-methylenemiltirone | Alpha-1D adrenergic receptor | | ADRA1D |
| MOL007049 | 4-methylenemiltirone | DNA topoisomerase II | | DHX9 |
| MOL007049 | 4-methylenemiltirone | Sodium-dependent serotonin transporter | | SLC6A4 |
| MOL007049 | 4-methylenemiltirone | D(2) dopamine receptor | | SNCA |
| MOL007049 | 4-methylenemiltirone | Mu-type opioid receptor | | OPRM1 |
| MOL007049 | 4-methylenemiltirone | Gamma-aminobutyric acid receptor subunit alpha-1 | | GABRA1 |
| MOL007049 | 4-methylenemiltirone | Neuronal acetylcholine receptor protein, alpha-7 chain | | CHRNA6 |
| MOL007049 | 4-methylenemiltirone | mRNA of PKA Catalytic Subunit C-alpha | | AKAP13 |
| MOL007049 | 4-methylenemiltirone | Nuclear receptor coactivator 2 | | BUD23 |
| MOL007049 | 4-methylenemiltirone | Nuclear receptor coactivator 1 | | BUD23 |
| MOL007050 | 2-(4-hydroxy-3-methoxyphenyl)-5-(3-hydroxypropyl)-7-methoxy-3-benzofurancarboxaldehyde | Nitric oxide synthase, inducible | | NOS2 |
| MOL007050 | 2-(4-hydroxy-3-methoxyphenyl)-5-(3-hydroxypropyl)-7-methoxy-3-benzofurancarboxaldehyde | Thrombin | | CXCL8 |
| MOL007050 | 2-(4-hydroxy-3-methoxyphenyl)-5-(3-hydroxypropyl)-7-methoxy-3-benzofurancarboxaldehyde | Estrogen receptor | | CTH |
| MOL007050 | 2-(4-hydroxy-3-methoxyphenyl)-5-(3-hydroxypropyl)-7-methoxy-3-benzofurancarboxaldehyde | Androgen receptor | | PKN1 |
| MOL007050 | 2-(4-hydroxy-3-methoxyphenyl)-5-(3-hydroxypropyl)-7-methoxy-3-benzofurancarboxaldehyde | Peroxisome proliferator activated receptor gamma | | PPARA |
| MOL007050 | 2-(4-hydroxy-3-methoxyphenyl)-5-(3-hydroxypropyl)-7-methoxy-3-benzofurancarboxaldehyde | Estrogen receptor beta | | PDGFRB |
| MOL007050 | 2-(4-hydroxy-3-methoxyphenyl)-5-(3-hydroxypropyl)-7-methoxy-3-benzofurancarboxaldehyde | Mitogen-activated protein kinase 14 | | MAPK14 |
| MOL007050 | 2-(4-hydroxy-3-methoxyphenyl)-5-(3-hydroxypropyl)-7-methoxy-3-benzofurancarboxaldehyde | Glycogen synthase kinase-3 beta | | GSK3B |
| MOL007050 | 2-(4-hydroxy-3-methoxyphenyl)-5-(3-hydroxypropyl)-7-methoxy-3-benzofurancarboxaldehyde | Heat shock protein HSP 90 | | HSPB1 |
| MOL007050 | 2-(4-hydroxy-3-methoxyphenyl)-5-(3-hydroxypropyl)-7-methoxy-3-benzofurancarboxaldehyde | Cell division protein kinase 2 | | CDK2 |
| MOL007050 | 2-(4-hydroxy-3-methoxyphenyl)-5-(3-hydroxypropyl)-7-methoxy-3-benzofurancarboxaldehyde | Proto-oncogene serine/threonine-protein kinase Pim-1 | | PIM1 |
| MOL007050 | 2-(4-hydroxy-3-methoxyphenyl)-5-(3-hydroxypropyl)-7-methoxy-3-benzofurancarboxaldehyde | Cyclin-A2 | | CCNA2 |
| MOL007058 | formyltanshinone | Thrombin | | CXCL8 |
| MOL007058 | formyltanshinone | Androgen receptor | | PKN1 |
| MOL007058 | formyltanshinone | Prostaglandin G/H synthase 2 | | PTGES3 |
| MOL007058 | formyltanshinone | Retinoic acid receptor RXR-alpha | | RXRA |
| MOL007058 | formyltanshinone | Dipeptidyl peptidase IV | | DPP4 |
| MOL007058 | formyltanshinone | Phosphatidylinositol-4,5-bisphosphate 3-kinase catalytic subunit, gamma isoform | | PIK3CG |
| MOL007058 | formyltanshinone | mRNA of PKA Catalytic Subunit C-alpha | | AKAP13 |
| MOL007058 | formyltanshinone | Nuclear receptor coactivator 1 | | BUD23 |
| MOL007059 | 3-beta-Hydroxymethyllenetanshiquinone | Dopamine D1 receptor | | DRD1 |
| MOL007059 | 3-beta-Hydroxymethyllenetanshiquinone | Thrombin | | CXCL8 |
| MOL007059 | 3-beta-Hydroxymethyllenetanshiquinone | Muscarinic acetylcholine receptor M1 | | CHRM1 |
| MOL007059 | 3-beta-Hydroxymethyllenetanshiquinone | Prostaglandin G/H synthase 2 | | PTGES3 |
| MOL007059 | 3-beta-Hydroxymethyllenetanshiquinone | Carbonic anhydrase II | | CA5A |
| MOL007059 | 3-beta-Hydroxymethyllenetanshiquinone | Retinoic acid receptor RXR-alpha | | RXRA |
| MOL007059 | 3-beta-Hydroxymethyllenetanshiquinone | Delta-type opioid receptor | | OPRD1 |
| MOL007059 | 3-beta-Hydroxymethyllenetanshiquinone | Acetylcholinesterase | | ACHE |
| MOL007059 | 3-beta-Hydroxymethyllenetanshiquinone | Alpha-1A adrenergic receptor | | ADRA1A |
| MOL007059 | 3-beta-Hydroxymethyllenetanshiquinone | Beta-2 adrenergic receptor | | GRK2 |
| MOL007059 | 3-beta-Hydroxymethyllenetanshiquinone | Mu-type opioid receptor | | OPRM1 |
| MOL007059 | 3-beta-Hydroxymethyllenetanshiquinone | Dipeptidyl peptidase IV | | DPP4 |
| MOL007059 | 3-beta-Hydroxymethyllenetanshiquinone | Heat shock protein HSP 90 | | HSPB1 |
| MOL007059 | 3-beta-Hydroxymethyllenetanshiquinone | Neuronal acetylcholine receptor protein, alpha-7 chain | | CHRNA6 |
| MOL007059 | 3-beta-Hydroxymethyllenetanshiquinone | Ig gamma-1 chain C region | | IGHG1 |
| MOL007059 | 3-beta-Hydroxymethyllenetanshiquinone | Trypsin-1 | | PRSS1 |
| MOL007059 | 3-beta-Hydroxymethyllenetanshiquinone | Nuclear receptor coactivator 1 | | BUD23 |
| MOL007061 | Methylenetanshinquinone | Dopamine D1 receptor | | DRD1 |
| MOL007061 | Methylenetanshinquinone | Muscarinic acetylcholine receptor M3 | | CHRM3 |
| MOL007061 | Methylenetanshinquinone | Thrombin | | CXCL8 |
| MOL007061 | Methylenetanshinquinone | Muscarinic acetylcholine receptor M1 | | CHRM1 |
| MOL007061 | Methylenetanshinquinone | Sodium channel protein type 5 subunit alpha | | SCNN1B |
| MOL007061 | Methylenetanshinquinone | Muscarinic acetylcholine receptor M5 | | CHRM5 |
| MOL007061 | Methylenetanshinquinone | Prostaglandin G/H synthase 2 | | PTGES3 |
| MOL007061 | Methylenetanshinquinone | Carbonic anhydrase II | | CA5A |
| MOL007061 | Methylenetanshinquinone | Retinoic acid receptor RXR-alpha | | RXRA |
| MOL007061 | Methylenetanshinquinone | Delta-type opioid receptor | | OPRD1 |
| MOL007061 | Methylenetanshinquinone | Acetylcholinesterase | | ACHE |
| MOL007061 | Methylenetanshinquinone | 5-hydroxytryptamine 2A receptor | | HTR2A |
| MOL007061 | Methylenetanshinquinone | Alpha-1A adrenergic receptor | | ADRA1A |
| MOL007061 | Methylenetanshinquinone | Muscarinic acetylcholine receptor M2 | | CHRM2 |
| MOL007061 | Methylenetanshinquinone | Beta-2 adrenergic receptor | | GRK2 |
| MOL007061 | Methylenetanshinquinone | Sodium-dependent serotonin transporter | | SLC6A4 |
| MOL007061 | Methylenetanshinquinone | Mu-type opioid receptor | | OPRM1 |
| MOL007061 | Methylenetanshinquinone | Gamma-aminobutyric acid receptor subunit alpha-1 | | GABRA1 |
| MOL007061 | Methylenetanshinquinone | Dipeptidyl peptidase IV | | DPP4 |
| MOL007061 | Methylenetanshinquinone | Heat shock protein HSP 90 | | HSPB1 |
| MOL007061 | Methylenetanshinquinone | Neuronal acetylcholine receptor protein, alpha-7 chain | | CHRNA6 |
| MOL007061 | Methylenetanshinquinone | Ig gamma-1 chain C region | | IGHG1 |
| MOL007061 | Methylenetanshinquinone | Trypsin-1 | | PRSS1 |
| MOL007061 | Methylenetanshinquinone | Nuclear receptor coactivator 1 | | BUD23 |
| MOL007063 | przewalskin a | Mineralocorticoid receptor | | BUD23 |
| MOL007063 | przewalskin a | Glucocorticoid receptor | | FKBP4 |
| MOL007064 | przewalskin b | Prostaglandin G/H synthase 2 | | PTGES3 |
| MOL007064 | przewalskin b | Progesterone receptor | | NR3C2 |
| MOL007064 | przewalskin b | Mineralocorticoid receptor | | BUD23 |
| MOL007064 | przewalskin b | Glucocorticoid receptor | | FKBP4 |
| MOL007064 | przewalskin b | Nuclear receptor coactivator 2 | | BUD23 |
| MOL007064 | przewalskin b | Nuclear receptor coactivator 1 | | BUD23 |
| MOL007068 | Przewaquinone B | Thrombin | | CXCL8 |
| MOL007068 | Przewaquinone B | Prostaglandin G/H synthase 2 | | PTGES3 |
| MOL007068 | Przewaquinone B | Retinoic acid receptor RXR-alpha | | RXRA |
| MOL007068 | Przewaquinone B | Dipeptidyl peptidase IV | | DPP4 |
| MOL007068 | Przewaquinone B | Heat shock protein HSP 90 | | HSPB1 |
| MOL007068 | Przewaquinone B | Phosphatidylinositol-4,5-bisphosphate 3-kinase catalytic subunit, gamma isoform | | PIK3CG |
| MOL007068 | Przewaquinone B | Ig gamma-1 chain C region | | IGHG1 |
| MOL007068 | Przewaquinone B | Trypsin-1 | | PRSS1 |
| MOL007068 | Przewaquinone B | Nuclear receptor coactivator 1 | | BUD23 |
| MOL007069 | przewaquinone c | Prostaglandin G/H synthase 1 | | PTGS1 |
| MOL007069 | przewaquinone c | Dopamine D1 receptor | | DRD1 |
| MOL007069 | przewaquinone c | Muscarinic acetylcholine receptor M3 | | CHRM3 |
| MOL007069 | przewaquinone c | Thrombin | | CXCL8 |
| MOL007069 | przewaquinone c | Muscarinic acetylcholine receptor M1 | | CHRM1 |
| MOL007069 | przewaquinone c | Sodium channel protein type 5 subunit alpha | | SCNN1B |
| MOL007069 | przewaquinone c | Muscarinic acetylcholine receptor M5 | | CHRM5 |
| MOL007069 | przewaquinone c | Prostaglandin G/H synthase 2 | | PTGES3 |
| MOL007069 | przewaquinone c | Carbonic anhydrase II | | CA5A |
| MOL007069 | przewaquinone c | Muscarinic acetylcholine receptor M4 | | CHRM4 |
| MOL007069 | przewaquinone c | Delta-type opioid receptor | | OPRD1 |
| MOL007069 | przewaquinone c | Acetylcholinesterase | | ACHE |
| MOL007069 | przewaquinone c | Alpha-1A adrenergic receptor | | ADRA1A |
| MOL007069 | przewaquinone c | Muscarinic acetylcholine receptor M2 | | CHRM2 |
| MOL007069 | przewaquinone c | Beta-2 adrenergic receptor | | GRK2 |
| MOL007069 | przewaquinone c | Mu-type opioid receptor | | OPRM1 |
| MOL007069 | przewaquinone c | Gamma-aminobutyric acid receptor subunit alpha-1 | | GABRA1 |
| MOL007069 | przewaquinone c | Dipeptidyl peptidase IV | | DPP4 |
| MOL007069 | przewaquinone c | Heat shock protein HSP 90 | | HSPB1 |
| MOL007069 | przewaquinone c | Phosphatidylinositol-4,5-bisphosphate 3-kinase catalytic subunit, gamma isoform | | PIK3CG |
| MOL007069 | przewaquinone c | Neuronal acetylcholine receptor protein, alpha-7 chain | | CHRNA6 |
| MOL007069 | przewaquinone c | Nuclear receptor coactivator 1 | | BUD23 |
| MOL007070 | (6S,7R)-6,7-dihydroxy-1,6-dimethyl-8,9-dihydro-7H-naphtho[8,7-g]benzofuran-10,11-dione | Thrombin | | CXCL8 |
| MOL007070 | (6S,7R)-6,7-dihydroxy-1,6-dimethyl-8,9-dihydro-7H-naphtho[8,7-g]benzofuran-10,11-dione | Prostaglandin G/H synthase 2 | | PTGES3 |
| MOL007070 | (6S,7R)-6,7-dihydroxy-1,6-dimethyl-8,9-dihydro-7H-naphtho[8,7-g]benzofuran-10,11-dione | Carbonic anhydrase II | | CA5A |
| MOL007070 | (6S,7R)-6,7-dihydroxy-1,6-dimethyl-8,9-dihydro-7H-naphtho[8,7-g]benzofuran-10,11-dione | Acetylcholinesterase | | ACHE |
| MOL007070 | (6S,7R)-6,7-dihydroxy-1,6-dimethyl-8,9-dihydro-7H-naphtho[8,7-g]benzofuran-10,11-dione | Dipeptidyl peptidase IV | | DPP4 |
| MOL007070 | (6S,7R)-6,7-dihydroxy-1,6-dimethyl-8,9-dihydro-7H-naphtho[8,7-g]benzofuran-10,11-dione | Heat shock protein HSP 90 | | HSPB1 |
| MOL007070 | (6S,7R)-6,7-dihydroxy-1,6-dimethyl-8,9-dihydro-7H-naphtho[8,7-g]benzofuran-10,11-dione | Trypsin-1 | | PRSS1 |
| MOL007070 | (6S,7R)-6,7-dihydroxy-1,6-dimethyl-8,9-dihydro-7H-naphtho[8,7-g]benzofuran-10,11-dione | Nuclear receptor coactivator 1 | | BUD23 |
| MOL007071 | przewaquinone f | Thrombin | | CXCL8 |
| MOL007071 | przewaquinone f | Prostaglandin G/H synthase 2 | | PTGES3 |
| MOL007071 | przewaquinone f | Dipeptidyl peptidase IV | | DPP4 |
| MOL007071 | przewaquinone f | Trypsin-1 | | PRSS1 |
| MOL007071 | przewaquinone f | Nuclear receptor coactivator 1 | | BUD23 |
| MOL007077 | sclareol | Prostaglandin G/H synthase 2 | | PTGES3 |
| MOL007079 | tanshinaldehyde | Dopamine D1 receptor | | DRD1 |
| MOL007079 | tanshinaldehyde | Thrombin | | CXCL8 |
| MOL007079 | tanshinaldehyde | Muscarinic acetylcholine receptor M1 | | CHRM1 |
| MOL007079 | tanshinaldehyde | Prostaglandin G/H synthase 2 | | PTGES3 |
| MOL007079 | tanshinaldehyde | Delta-type opioid receptor | | OPRD1 |
| MOL007079 | tanshinaldehyde | Acetylcholinesterase | | ACHE |
| MOL007079 | tanshinaldehyde | 5-hydroxytryptamine 2A receptor | | HTR2A |
| MOL007079 | tanshinaldehyde | Beta-2 adrenergic receptor | | GRK2 |
| MOL007079 | tanshinaldehyde | Mu-type opioid receptor | | OPRM1 |
| MOL007079 | tanshinaldehyde | Dipeptidyl peptidase IV | | DPP4 |
| MOL007079 | tanshinaldehyde | Neuronal acetylcholine receptor protein, alpha-7 chain | | CHRNA6 |
| MOL007079 | tanshinaldehyde | Trypsin-1 | | PRSS1 |
| MOL007079 | tanshinaldehyde | Nuclear receptor coactivator 1 | | BUD23 |
| MOL007081 | Danshenol B | Prostaglandin G/H synthase 2 | | PTGES3 |
| MOL007081 | Danshenol B | Carbonic anhydrase II | | CA5A |
| MOL007081 | Danshenol B | Progesterone receptor | | NR3C2 |
| MOL007081 | Danshenol B | DNA topoisomerase II | | DHX9 |
| MOL007081 | Danshenol B | Mu-type opioid receptor | | OPRM1 |
| MOL007081 | Danshenol B | Glucocorticoid receptor | | FKBP4 |
| MOL007081 | Danshenol B | Heat shock protein HSP 90 | | HSPB1 |
| MOL007081 | Danshenol B | Nuclear receptor coactivator 1 | | BUD23 |
| MOL007082 | Danshenol A | Prostaglandin G/H synthase 1 | | PTGS1 |
| MOL007082 | Danshenol A | Potassium voltage-gated channel subfamily H member 2 | | KCNQ5 |
| MOL007082 | Danshenol A | Sodium channel protein type 5 subunit alpha | | SCNN1B |
| MOL007082 | Danshenol A | Coagulation factor Xa | | F2RL1 |
| MOL007082 | Danshenol A | Prostaglandin G/H synthase 2 | | PTGES3 |
| MOL007082 | Danshenol A | Retinoic acid receptor RXR-alpha | | RXRA |
| MOL007082 | Danshenol A | Phosphatidylinositol-4,5-bisphosphate 3-kinase catalytic subunit, gamma isoform | | PIK3CG |
| MOL007082 | Danshenol A | Nuclear receptor coactivator 1 | | BUD23 |
| MOL007082 | Danshenol A | Calcium-activated potassium channel subunit alpha 1 | | KCNMA1 |
| MOL007085 | Salvilenone | Prostaglandin G/H synthase 1 | | PTGS1 |
| MOL007085 | Salvilenone | Estrogen receptor | | CTH |
| MOL007085 | Salvilenone | Androgen receptor | | PKN1 |
| MOL007085 | Salvilenone | Muscarinic acetylcholine receptor M5 | | CHRM5 |
| MOL007085 | Salvilenone | Prostaglandin G/H synthase 2 | | PTGES3 |
| MOL007085 | Salvilenone | 5-hydroxytryptamine receptor 3A | | HTR3A |
| MOL007085 | Salvilenone | Estrogen receptor beta | | PDGFRB |
| MOL007085 | Salvilenone | Proto-oncogene serine/threonine-protein kinase Pim-1 | | PIM1 |
| MOL007088 | cryptotanshinone | Prostaglandin G/H synthase 1 | | PTGS1 |
| MOL007088 | cryptotanshinone | Dopamine D1 receptor | | DRD1 |
| MOL007088 | cryptotanshinone | Muscarinic acetylcholine receptor M3 | | CHRM3 |
| MOL007088 | cryptotanshinone | Muscarinic acetylcholine receptor M1 | | CHRM1 |
| MOL007088 | cryptotanshinone | Sodium channel protein type 5 subunit alpha | | SCNN1B |
| MOL007088 | cryptotanshinone | Muscarinic acetylcholine receptor M5 | | CHRM5 |
| MOL007088 | cryptotanshinone | Prostaglandin G/H synthase 2 | | PTGES3 |
| MOL007088 | cryptotanshinone | Carbonic anhydrase II | | CA5A |
| MOL007088 | cryptotanshinone | Muscarinic acetylcholine receptor M4 | | CHRM4 |
| MOL007088 | cryptotanshinone | Delta-type opioid receptor | | OPRD1 |
| MOL007088 | cryptotanshinone | Alpha-1A adrenergic receptor | | ADRA1A |
| MOL007088 | cryptotanshinone | Muscarinic acetylcholine receptor M2 | | CHRM2 |
| MOL007088 | cryptotanshinone | Alpha-1B adrenergic receptor | | ADRA1B |
| MOL007088 | cryptotanshinone | Beta-2 adrenergic receptor | | GRK2 |
| MOL007088 | cryptotanshinone | Alpha-1D adrenergic receptor | | ADRA1D |
| MOL007088 | cryptotanshinone | DNA topoisomerase II | | DHX9 |
| MOL007088 | cryptotanshinone | Mu-type opioid receptor | | OPRM1 |
| MOL007088 | cryptotanshinone | Neuronal acetylcholine receptor protein, alpha-7 chain | | CHRNA6 |
| MOL007088 | cryptotanshinone | Nuclear receptor coactivator 2 | | BUD23 |
| MOL007088 | cryptotanshinone | Nuclear receptor coactivator 1 | | BUD23 |
| MOL007088 | cryptotanshinone | Progesterone receptor | | NR3C2 |
| MOL007088 | cryptotanshinone | Gamma-aminobutyric acid receptor subunit alpha-1 | | GABRA1 |
| MOL007088 | cryptotanshinone | Transcription factor p65 | | CDK9 |
| MOL007088 | cryptotanshinone | Signal transducer and activator of transcription 3 | | FOXH1 |
| MOL007088 | cryptotanshinone | G1/S-specific cyclin-D1 | | CCND3 |
| MOL007088 | cryptotanshinone | Bcl-2-like protein 1 | | BAK1 |
| MOL007088 | cryptotanshinone | Tumor necrosis factor | | TNF |
| MOL007088 | cryptotanshinone | Amyloid beta A4 protein | | APP |
| MOL007088 | cryptotanshinone | Endothelin-1 | | EDN1 |
| MOL007088 | cryptotanshinone | Baculoviral IAP repeat-containing protein 5 | | BIRC8 |
| MOL007093 | dan-shexinkum d | Prostaglandin G/H synthase 1 | | PTGS1 |
| MOL007093 | dan-shexinkum d | Dopamine D1 receptor | | DRD1 |
| MOL007093 | dan-shexinkum d | Muscarinic acetylcholine receptor M3 | | CHRM3 |
| MOL007093 | dan-shexinkum d | Muscarinic acetylcholine receptor M1 | | CHRM1 |
| MOL007093 | dan-shexinkum d | Estrogen receptor | | CTH |
| MOL007093 | dan-shexinkum d | Androgen receptor | | PKN1 |
| MOL007093 | dan-shexinkum d | Sodium channel protein type 5 subunit alpha | | SCNN1B |
| MOL007093 | dan-shexinkum d | Muscarinic acetylcholine receptor M5 | | CHRM5 |
| MOL007093 | dan-shexinkum d | Prostaglandin G/H synthase 2 | | PTGES3 |
| MOL007093 | dan-shexinkum d | Nitric-oxide synthase, endothelial | | KDR |
| MOL007093 | dan-shexinkum d | Carbonic anhydrase II | | CA5A |
| MOL007093 | dan-shexinkum d | Muscarinic acetylcholine receptor M4 | | CHRM4 |
| MOL007093 | dan-shexinkum d | Retinoic acid receptor RXR-alpha | | RXRA |
| MOL007093 | dan-shexinkum d | Delta-type opioid receptor | | OPRD1 |
| MOL007093 | dan-shexinkum d | Alpha-1A adrenergic receptor | | ADRA1A |
| MOL007093 | dan-shexinkum d | Muscarinic acetylcholine receptor M2 | | CHRM2 |
| MOL007093 | dan-shexinkum d | Alpha-1B adrenergic receptor | | ADRA1B |
| MOL007093 | dan-shexinkum d | Beta-2 adrenergic receptor | | GRK2 |
| MOL007093 | dan-shexinkum d | Alpha-1D adrenergic receptor | | ADRA1D |
| MOL007093 | dan-shexinkum d | DNA topoisomerase II | | DHX9 |
| MOL007093 | dan-shexinkum d | Mu-type opioid receptor | | OPRM1 |
| MOL007093 | dan-shexinkum d | Glycogen synthase kinase-3 beta | | GSK3B |
| MOL007093 | dan-shexinkum d | Cell division protein kinase 2 | | CDK2 |
| MOL007093 | dan-shexinkum d | Neuronal acetylcholine receptor protein, alpha-7 chain | | CHRNA6 |
| MOL007093 | dan-shexinkum d | Ig gamma-1 chain C region | | IGHG1 |
| MOL007093 | dan-shexinkum d | Proto-oncogene serine/threonine-protein kinase Pim-1 | | PIM1 |
| MOL007093 | dan-shexinkum d | Nuclear receptor coactivator 2 | | BUD23 |
| MOL007093 | dan-shexinkum d | Nuclear receptor coactivator 1 | | BUD23 |
| MOL007094 | danshenspiroketallactone | Prostaglandin G/H synthase 1 | | PTGS1 |
| MOL007094 | danshenspiroketallactone | Dopamine D1 receptor | | DRD1 |
| MOL007094 | danshenspiroketallactone | Muscarinic acetylcholine receptor M3 | | CHRM3 |
| MOL007094 | danshenspiroketallactone | Thrombin | | CXCL8 |
| MOL007094 | danshenspiroketallactone | Muscarinic acetylcholine receptor M1 | | CHRM1 |
| MOL007094 | danshenspiroketallactone | Estrogen receptor | | CTH |
| MOL007094 | danshenspiroketallactone | Sodium channel protein type 5 subunit alpha | | SCNN1B |
| MOL007094 | danshenspiroketallactone | Muscarinic acetylcholine receptor M5 | | CHRM5 |
| MOL007094 | danshenspiroketallactone | Prostaglandin G/H synthase 2 | | PTGES3 |
| MOL007094 | danshenspiroketallactone | Carbonic anhydrase II | | CA5A |
| MOL007094 | danshenspiroketallactone | Muscarinic acetylcholine receptor M4 | | CHRM4 |
| MOL007094 | danshenspiroketallactone | Retinoic acid receptor RXR-alpha | | RXRA |
| MOL007094 | danshenspiroketallactone | Acetylcholinesterase | | ACHE |
| MOL007094 | danshenspiroketallactone | Alpha-1A adrenergic receptor | | ADRA1A |
| MOL007094 | danshenspiroketallactone | Muscarinic acetylcholine receptor M2 | | CHRM2 |
| MOL007094 | danshenspiroketallactone | Alpha-1B adrenergic receptor | | ADRA1B |
| MOL007094 | danshenspiroketallactone | Beta-2 adrenergic receptor | | GRK2 |
| MOL007094 | danshenspiroketallactone | Alpha-1D adrenergic receptor | | ADRA1D |
| MOL007094 | danshenspiroketallactone | Neuronal acetylcholine receptor subunit alpha-2 | | CHRNA3 |
| MOL007094 | danshenspiroketallactone | Sodium-dependent serotonin transporter | | SLC6A4 |
| MOL007094 | danshenspiroketallactone | Mu-type opioid receptor | | OPRM1 |
| MOL007094 | danshenspiroketallactone | Gamma-aminobutyric acid receptor subunit alpha-1 | | GABRA1 |
| MOL007094 | danshenspiroketallactone | Dipeptidyl peptidase IV | | DPP4 |
| MOL007094 | danshenspiroketallactone | Heat shock protein HSP 90 | | HSPB1 |
| MOL007094 | danshenspiroketallactone | Neuronal acetylcholine receptor protein, alpha-7 chain | | CHRNA6 |
| MOL007098 | deoxyneocryptotanshinone | Prostaglandin G/H synthase 1 | | PTGS1 |
| MOL007098 | deoxyneocryptotanshinone | Dopamine D1 receptor | | DRD1 |
| MOL007098 | deoxyneocryptotanshinone | Muscarinic acetylcholine receptor M3 | | CHRM3 |
| MOL007098 | deoxyneocryptotanshinone | Muscarinic acetylcholine receptor M1 | | CHRM1 |
| MOL007098 | deoxyneocryptotanshinone | Estrogen receptor | | CTH |
| MOL007098 | deoxyneocryptotanshinone | Androgen receptor | | PKN1 |
| MOL007098 | deoxyneocryptotanshinone | Sodium channel protein type 5 subunit alpha | | SCNN1B |
| MOL007098 | deoxyneocryptotanshinone | Muscarinic acetylcholine receptor M5 | | CHRM5 |
| MOL007098 | deoxyneocryptotanshinone | Prostaglandin G/H synthase 2 | | PTGES3 |
| MOL007098 | deoxyneocryptotanshinone | Nitric-oxide synthase, endothelial | | KDR |
| MOL007098 | deoxyneocryptotanshinone | Carbonic anhydrase II | | CA5A |
| MOL007098 | deoxyneocryptotanshinone | Muscarinic acetylcholine receptor M4 | | CHRM4 |
| MOL007098 | deoxyneocryptotanshinone | Retinoic acid receptor RXR-alpha | | RXRA |
| MOL007098 | deoxyneocryptotanshinone | Delta-type opioid receptor | | OPRD1 |
| MOL007098 | deoxyneocryptotanshinone | Alpha-1A adrenergic receptor | | ADRA1A |
| MOL007098 | deoxyneocryptotanshinone | Muscarinic acetylcholine receptor M2 | | CHRM2 |
| MOL007098 | deoxyneocryptotanshinone | Alpha-1B adrenergic receptor | | ADRA1B |
| MOL007098 | deoxyneocryptotanshinone | Beta-2 adrenergic receptor | | GRK2 |
| MOL007098 | deoxyneocryptotanshinone | Alpha-1D adrenergic receptor | | ADRA1D |
| MOL007098 | deoxyneocryptotanshinone | DNA topoisomerase II | | DHX9 |
| MOL007098 | deoxyneocryptotanshinone | Mu-type opioid receptor | | OPRM1 |
| MOL007098 | deoxyneocryptotanshinone | Glycogen synthase kinase-3 beta | | GSK3B |
| MOL007098 | deoxyneocryptotanshinone | Cell division protein kinase 2 | | CDK2 |
| MOL007098 | deoxyneocryptotanshinone | Neuronal acetylcholine receptor protein, alpha-7 chain | | CHRNA6 |
| MOL007098 | deoxyneocryptotanshinone | Ig gamma-1 chain C region | | IGHG1 |
| MOL007098 | deoxyneocryptotanshinone | Proto-oncogene serine/threonine-protein kinase Pim-1 | | PIM1 |
| MOL007098 | deoxyneocryptotanshinone | Nuclear receptor coactivator 2 | | BUD23 |
| MOL007098 | deoxyneocryptotanshinone | Nuclear receptor coactivator 1 | | BUD23 |
| MOL007100 | dihydrotanshinlactone | Nitric oxide synthase, inducible | | NOS2 |
| MOL007100 | dihydrotanshinlactone | Prostaglandin G/H synthase 1 | | PTGS1 |
| MOL007100 | dihydrotanshinlactone | Dopamine D1 receptor | | DRD1 |
| MOL007100 | dihydrotanshinlactone | Muscarinic acetylcholine receptor M3 | | CHRM3 |
| MOL007100 | dihydrotanshinlactone | Thrombin | | CXCL8 |
| MOL007100 | dihydrotanshinlactone | Muscarinic acetylcholine receptor M1 | | CHRM1 |
| MOL007100 | dihydrotanshinlactone | Estrogen receptor | | CTH |
| MOL007100 | dihydrotanshinlactone | Androgen receptor | | PKN1 |
| MOL007100 | dihydrotanshinlactone | Sodium channel protein type 5 subunit alpha | | SCNN1B |
| MOL007100 | dihydrotanshinlactone | Peroxisome proliferator activated receptor gamma | | PPARA |
| MOL007100 | dihydrotanshinlactone | Muscarinic acetylcholine receptor M5 | | CHRM5 |
| MOL007100 | dihydrotanshinlactone | Prostaglandin G/H synthase 2 | | PTGES3 |
| MOL007100 | dihydrotanshinlactone | 5-hydroxytryptamine receptor 3A | | HTR3A |
| MOL007100 | dihydrotanshinlactone | Carbonic anhydrase II | | CA5A |
| MOL007100 | dihydrotanshinlactone | Retinoic acid receptor RXR-alpha | | RXRA |
| MOL007100 | dihydrotanshinlactone | Acetylcholinesterase | | ACHE |
| MOL007100 | dihydrotanshinlactone | CGMP-inhibited 3',5'-cyclic phosphodiesterase A | | PDE3A |
| MOL007100 | dihydrotanshinlactone | 5-hydroxytryptamine 2A receptor | | HTR2A |
| MOL007100 | dihydrotanshinlactone | Alpha-1A adrenergic receptor | | ADRA1A |
| MOL007100 | dihydrotanshinlactone | Alpha-1B adrenergic receptor | | ADRA1B |
| MOL007100 | dihydrotanshinlactone | mRNA of Protein-tyrosine phosphatase, non-receptor type 1 | | PTPN22 |
| MOL007100 | dihydrotanshinlactone | Sodium-dependent dopamine transporter | | SLC6A3 |
| MOL007100 | dihydrotanshinlactone | Beta-2 adrenergic receptor | | GRK2 |
| MOL007100 | dihydrotanshinlactone | Alpha-1D adrenergic receptor | | ADRA1D |
| MOL007100 | dihydrotanshinlactone | Sodium-dependent serotonin transporter | | SLC6A4 |
| MOL007100 | dihydrotanshinlactone | Mu-type opioid receptor | | OPRM1 |
| MOL007100 | dihydrotanshinlactone | Gamma-aminobutyric acid receptor subunit alpha-1 | | GABRA1 |
| MOL007100 | dihydrotanshinlactone | Dipeptidyl peptidase IV | | DPP4 |
| MOL007100 | dihydrotanshinlactone | Glycogen synthase kinase-3 beta | | GSK3B |
| MOL007100 | dihydrotanshinlactone | Phosphatidylinositol-4,5-bisphosphate 3-kinase catalytic subunit, gamma isoform | | PIK3CG |
| MOL007100 | dihydrotanshinlactone | Neuronal acetylcholine receptor protein, alpha-7 chain | | CHRNA6 |
| MOL007100 | dihydrotanshinlactone | mRNA of PKA Catalytic Subunit C-alpha | | AKAP13 |
| MOL007100 | dihydrotanshinlactone | Ig gamma-1 chain C region | | IGHG1 |
| MOL007100 | dihydrotanshinlactone | Trypsin-1 | | PRSS1 |
| MOL007100 | dihydrotanshinlactone | Proto-oncogene serine/threonine-protein kinase Pim-1 | | PIM1 |
| MOL007100 | dihydrotanshinlactone | Cyclin-A2 | | CCNA2 |
| MOL007101 | dihydrotanshinoneⅠ | Prostaglandin G/H synthase 1 | | PTGS1 |
| MOL007101 | dihydrotanshinoneⅠ | Sodium channel protein type 5 subunit alpha | | SCNN1B |
| MOL007101 | dihydrotanshinoneⅠ | Prostaglandin G/H synthase 2 | | PTGES3 |
| MOL007101 | dihydrotanshinoneⅠ | 5-hydroxytryptamine receptor 3A | | HTR3A |
| MOL007101 | dihydrotanshinoneⅠ | Retinoic acid receptor RXR-alpha | | RXRA |
| MOL007101 | dihydrotanshinoneⅠ | Alpha-1A adrenergic receptor | | ADRA1A |
| MOL007101 | dihydrotanshinoneⅠ | Alpha-1B adrenergic receptor | | ADRA1B |
| MOL007101 | dihydrotanshinoneⅠ | Beta-2 adrenergic receptor | | GRK2 |
| MOL007101 | dihydrotanshinoneⅠ | Gamma-aminobutyric acid receptor subunit alpha-1 | | GABRA1 |
| MOL007101 | dihydrotanshinoneⅠ | Heat shock protein HSP 90 | | HSPB1 |
| MOL007101 | dihydrotanshinoneⅠ | Phosphatidylinositol-4,5-bisphosphate 3-kinase catalytic subunit, gamma isoform | | PIK3CG |
| MOL007101 | dihydrotanshinoneⅠ | Neuronal acetylcholine receptor protein, alpha-7 chain | | CHRNA6 |
| MOL007101 | dihydrotanshinoneⅠ | mRNA of PKA Catalytic Subunit C-alpha | | AKAP13 |
| MOL007101 | dihydrotanshinoneⅠ | Ig gamma-1 chain C region | | IGHG1 |
| MOL007101 | dihydrotanshinoneⅠ | Nuclear receptor coactivator 2 | | BUD23 |
| MOL007101 | dihydrotanshinoneⅠ | Nuclear receptor coactivator 1 | | BUD23 |
| MOL007101 | dihydrotanshinoneⅠ | Calmodulin | | IQCB1 |
| MOL007105 | epidanshenspiroketallactone | Prostaglandin G/H synthase 1 | | PTGS1 |
| MOL007105 | epidanshenspiroketallactone | Dopamine D1 receptor | | DRD1 |
| MOL007105 | epidanshenspiroketallactone | Muscarinic acetylcholine receptor M3 | | CHRM3 |
| MOL007105 | epidanshenspiroketallactone | Muscarinic acetylcholine receptor M1 | | CHRM1 |
| MOL007105 | epidanshenspiroketallactone | Estrogen receptor | | CTH |
| MOL007105 | epidanshenspiroketallactone | Sodium channel protein type 5 subunit alpha | | SCNN1B |
| MOL007105 | epidanshenspiroketallactone | Muscarinic acetylcholine receptor M5 | | CHRM5 |
| MOL007105 | epidanshenspiroketallactone | Prostaglandin G/H synthase 2 | | PTGES3 |
| MOL007105 | epidanshenspiroketallactone | Nitric-oxide synthase, endothelial | | KDR |
| MOL007105 | epidanshenspiroketallactone | Muscarinic acetylcholine receptor M4 | | CHRM4 |
| MOL007105 | epidanshenspiroketallactone | Retinoic acid receptor RXR-alpha | | RXRA |
| MOL007105 | epidanshenspiroketallactone | Delta-type opioid receptor | | OPRD1 |
| MOL007105 | epidanshenspiroketallactone | CGMP-inhibited 3',5'-cyclic phosphodiesterase A | | PDE3A |
| MOL007105 | epidanshenspiroketallactone | 5-hydroxytryptamine 2A receptor | | HTR2A |
| MOL007105 | epidanshenspiroketallactone | Alpha-1A adrenergic receptor | | ADRA1A |
| MOL007105 | epidanshenspiroketallactone | Muscarinic acetylcholine receptor M2 | | CHRM2 |
| MOL007105 | epidanshenspiroketallactone | Alpha-1B adrenergic receptor | | ADRA1B |
| MOL007105 | epidanshenspiroketallactone | Beta-2 adrenergic receptor | | GRK2 |
| MOL007105 | epidanshenspiroketallactone | Alpha-1D adrenergic receptor | | ADRA1D |
| MOL007105 | epidanshenspiroketallactone | Sodium-dependent serotonin transporter | | SLC6A4 |
| MOL007105 | epidanshenspiroketallactone | Mu-type opioid receptor | | OPRM1 |
| MOL007105 | epidanshenspiroketallactone | Gamma-aminobutyric acid receptor subunit alpha-1 | | GABRA1 |
| MOL007105 | epidanshenspiroketallactone | Heat shock protein HSP 90 | | HSPB1 |
| MOL007105 | epidanshenspiroketallactone | Cell division protein kinase 2 | | CDK2 |
| MOL007105 | epidanshenspiroketallactone | Neuronal acetylcholine receptor protein, alpha-7 chain | | CHRNA6 |
| MOL007105 | epidanshenspiroketallactone | Proto-oncogene serine/threonine-protein kinase Pim-1 | | PIM1 |
| MOL007107 | C09092 | Muscarinic acetylcholine receptor M3 | | CHRM3 |
| MOL007107 | C09092 | Thrombin | | CXCL8 |
| MOL007107 | C09092 | Muscarinic acetylcholine receptor M1 | | CHRM1 |
| MOL007107 | C09092 | Sodium channel protein type 5 subunit alpha | | SCNN1B |
| MOL007107 | C09092 | Carbonic anhydrase II | | CA5A |
| MOL007107 | C09092 | Acetylcholinesterase | | ACHE |
| MOL007107 | C09092 | Alpha-1A adrenergic receptor | | ADRA1A |
| MOL007107 | C09092 | Muscarinic acetylcholine receptor M2 | | CHRM2 |
| MOL007107 | C09092 | Alpha-1B adrenergic receptor | | ADRA1B |
| MOL007107 | C09092 | Beta-2 adrenergic receptor | | GRK2 |
| MOL007107 | C09092 | Alpha-1D adrenergic receptor | | ADRA1D |
| MOL007107 | C09092 | Mu-type opioid receptor | | OPRM1 |
| MOL007108 | isocryptotanshi-none | Nitric oxide synthase, inducible | | NOS2 |
| MOL007108 | isocryptotanshi-none | Prostaglandin G/H synthase 1 | | PTGS1 |
| MOL007108 | isocryptotanshi-none | Dopamine D1 receptor | | DRD1 |
| MOL007108 | isocryptotanshi-none | Muscarinic acetylcholine receptor M3 | | CHRM3 |
| MOL007108 | isocryptotanshi-none | Muscarinic acetylcholine receptor M1 | | CHRM1 |
| MOL007108 | isocryptotanshi-none | Estrogen receptor | | CTH |
| MOL007108 | isocryptotanshi-none | Androgen receptor | | PKN1 |
| MOL007108 | isocryptotanshi-none | Sodium channel protein type 5 subunit alpha | | SCNN1B |
| MOL007108 | isocryptotanshi-none | Coagulation factor Xa | | F2RL1 |
| MOL007108 | isocryptotanshi-none | Muscarinic acetylcholine receptor M5 | | CHRM5 |
| MOL007108 | isocryptotanshi-none | Prostaglandin G/H synthase 2 | | PTGES3 |
| MOL007108 | isocryptotanshi-none | Carbonic anhydrase II | | CA5A |
| MOL007108 | isocryptotanshi-none | Muscarinic acetylcholine receptor M4 | | CHRM4 |
| MOL007108 | isocryptotanshi-none | Retinoic acid receptor RXR-alpha | | RXRA |
| MOL007108 | isocryptotanshi-none | Delta-type opioid receptor | | OPRD1 |
| MOL007108 | isocryptotanshi-none | Acetylcholinesterase | | ACHE |
| MOL007108 | isocryptotanshi-none | Alpha-1A adrenergic receptor | | ADRA1A |
| MOL007108 | isocryptotanshi-none | Muscarinic acetylcholine receptor M2 | | CHRM2 |
| MOL007108 | isocryptotanshi-none | Alpha-1B adrenergic receptor | | ADRA1B |
| MOL007108 | isocryptotanshi-none | Beta-2 adrenergic receptor | | GRK2 |
| MOL007108 | isocryptotanshi-none | Alpha-1D adrenergic receptor | | ADRA1D |
| MOL007108 | isocryptotanshi-none | DNA topoisomerase II | | DHX9 |
| MOL007108 | isocryptotanshi-none | D(2) dopamine receptor | | SNCA |
| MOL007108 | isocryptotanshi-none | Mu-type opioid receptor | | OPRM1 |
| MOL007108 | isocryptotanshi-none | Gamma-aminobutyric acid receptor subunit alpha-1 | | GABRA1 |
| MOL007108 | isocryptotanshi-none | Cell division protein kinase 2 | | CDK2 |
| MOL007108 | isocryptotanshi-none | Neuronal acetylcholine receptor protein, alpha-7 chain | | CHRNA6 |
| MOL007108 | isocryptotanshi-none | Trypsin-1 | | PRSS1 |
| MOL007108 | isocryptotanshi-none | Proto-oncogene serine/threonine-protein kinase Pim-1 | | PIM1 |
| MOL007108 | isocryptotanshi-none | Nuclear receptor coactivator 2 | | BUD23 |
| MOL007108 | isocryptotanshi-none | Nuclear receptor coactivator 1 | | BUD23 |
| MOL007111 | Isotanshinone II | Nitric oxide synthase, inducible | | NOS2 |
| MOL007111 | Isotanshinone II | Dopamine D1 receptor | | DRD1 |
| MOL007111 | Isotanshinone II | Muscarinic acetylcholine receptor M3 | | CHRM3 |
| MOL007111 | Isotanshinone II | Thrombin | | CXCL8 |
| MOL007111 | Isotanshinone II | Muscarinic acetylcholine receptor M1 | | CHRM1 |
| MOL007111 | Isotanshinone II | Estrogen receptor | | CTH |
| MOL007111 | Isotanshinone II | Androgen receptor | | PKN1 |
| MOL007111 | Isotanshinone II | Sodium channel protein type 5 subunit alpha | | SCNN1B |
| MOL007111 | Isotanshinone II | Muscarinic acetylcholine receptor M5 | | CHRM5 |
| MOL007111 | Isotanshinone II | Prostaglandin G/H synthase 2 | | PTGES3 |
| MOL007111 | Isotanshinone II | Retinoic acid receptor RXR-alpha | | RXRA |
| MOL007111 | Isotanshinone II | Delta-type opioid receptor | | OPRD1 |
| MOL007111 | Isotanshinone II | Acetylcholinesterase | | ACHE |
| MOL007111 | Isotanshinone II | Alpha-1A adrenergic receptor | | ADRA1A |
| MOL007111 | Isotanshinone II | Muscarinic acetylcholine receptor M2 | | CHRM2 |
| MOL007111 | Isotanshinone II | Beta-2 adrenergic receptor | | GRK2 |
| MOL007111 | Isotanshinone II | Mu-type opioid receptor | | OPRM1 |
| MOL007111 | Isotanshinone II | Estrogen receptor beta | | PDGFRB |
| MOL007111 | Isotanshinone II | Gamma-aminobutyric acid receptor subunit alpha-1 | | GABRA1 |
| MOL007111 | Isotanshinone II | Dipeptidyl peptidase IV | | DPP4 |
| MOL007111 | Isotanshinone II | Glycogen synthase kinase-3 beta | | GSK3B |
| MOL007111 | Isotanshinone II | Cell division protein kinase 2 | | CDK2 |
| MOL007111 | Isotanshinone II | Neuronal acetylcholine receptor protein, alpha-7 chain | | CHRNA6 |
| MOL007111 | Isotanshinone II | Serine/threonine-protein kinase Chk1 | | CDK1 |
| MOL007111 | Isotanshinone II | Proto-oncogene serine/threonine-protein kinase Pim-1 | | PIM1 |
| MOL007111 | Isotanshinone II | Cyclin-A2 | | CCNA2 |
| MOL007115 | manool | Nuclear receptor coactivator 2 | | BUD23 |
| MOL007119 | miltionone Ⅰ | Prostaglandin G/H synthase 1 | | PTGS1 |
| MOL007119 | miltionone Ⅰ | Muscarinic acetylcholine receptor M3 | | CHRM3 |
| MOL007119 | miltionone Ⅰ | Muscarinic acetylcholine receptor M1 | | CHRM1 |
| MOL007119 | miltionone Ⅰ | Estrogen receptor | | CTH |
| MOL007119 | miltionone Ⅰ | Androgen receptor | | PKN1 |
| MOL007119 | miltionone Ⅰ | Sodium channel protein type 5 subunit alpha | | SCNN1B |
| MOL007119 | miltionone Ⅰ | Coagulation factor Xa | | F2RL1 |
| MOL007119 | miltionone Ⅰ | Prostaglandin G/H synthase 2 | | PTGES3 |
| MOL007119 | miltionone Ⅰ | Carbonic anhydrase II | | CA5A |
| MOL007119 | miltionone Ⅰ | Retinoic acid receptor RXR-alpha | | RXRA |
| MOL007119 | miltionone Ⅰ | Delta-type opioid receptor | | OPRD1 |
| MOL007119 | miltionone Ⅰ | 5-hydroxytryptamine 2A receptor | | HTR2A |
| MOL007119 | miltionone Ⅰ | Alpha-1A adrenergic receptor | | ADRA1A |
| MOL007119 | miltionone Ⅰ | Muscarinic acetylcholine receptor M2 | | CHRM2 |
| MOL007119 | miltionone Ⅰ | Alpha-1B adrenergic receptor | | ADRA1B |
| MOL007119 | miltionone Ⅰ | Beta-2 adrenergic receptor | | GRK2 |
| MOL007119 | miltionone Ⅰ | DNA topoisomerase II | | DHX9 |
| MOL007119 | miltionone Ⅰ | Mu-type opioid receptor | | OPRM1 |
| MOL007119 | miltionone Ⅰ | Glucocorticoid receptor | | FKBP4 |
| MOL007119 | miltionone Ⅰ | Glycogen synthase kinase-3 beta | | GSK3B |
| MOL007119 | miltionone Ⅰ | Cell division protein kinase 2 | | CDK2 |
| MOL007119 | miltionone Ⅰ | Beta-lactamase | | COA7 |
| MOL007119 | miltionone Ⅰ | Neuronal acetylcholine receptor protein, alpha-7 chain | | CHRNA6 |
| MOL007119 | miltionone Ⅰ | Ig gamma-1 chain C region | | IGHG1 |
| MOL007119 | miltionone Ⅰ | Proto-oncogene serine/threonine-protein kinase Pim-1 | | PIM1 |
| MOL007119 | miltionone Ⅰ | Cyclin-A2 | | CCNA2 |
| MOL007119 | miltionone Ⅰ | Nuclear receptor coactivator 2 | | BUD23 |
| MOL007119 | miltionone Ⅰ | Nuclear receptor coactivator 1 | | BUD23 |
| MOL007120 | miltionone Ⅱ | Thrombin | | CXCL8 |
| MOL007120 | miltionone Ⅱ | Prostaglandin G/H synthase 2 | | PTGES3 |
| MOL007120 | miltionone Ⅱ | Carbonic anhydrase II | | CA5A |
| MOL007120 | miltionone Ⅱ | Acetylcholinesterase | | ACHE |
| MOL007120 | miltionone Ⅱ | Progesterone receptor | | NR3C2 |
| MOL007120 | miltionone Ⅱ | Glucocorticoid receptor | | FKBP4 |
| MOL007120 | miltionone Ⅱ | Nuclear receptor coactivator 2 | | BUD23 |
| MOL007120 | miltionone Ⅱ | Nuclear receptor coactivator 1 | | BUD23 |
| MOL007121 | miltipolone | Estrogen receptor | | CTH |
| MOL007121 | miltipolone | Acetylcholinesterase | | ACHE |
| MOL007122 | Miltirone | Prostaglandin G/H synthase 1 | | PTGS1 |
| MOL007122 | Miltirone | Dopamine D1 receptor | | DRD1 |
| MOL007122 | Miltirone | Muscarinic acetylcholine receptor M3 | | CHRM3 |
| MOL007122 | Miltirone | Muscarinic acetylcholine receptor M1 | | CHRM1 |
| MOL007122 | Miltirone | Estrogen receptor | | CTH |
| MOL007122 | Miltirone | Androgen receptor | | PKN1 |
| MOL007122 | Miltirone | D(1B) dopamine receptor | | GABBR1 |
| MOL007122 | Miltirone | Sodium channel protein type 5 subunit alpha | | SCNN1B |
| MOL007122 | Miltirone | Muscarinic acetylcholine receptor M5 | | CHRM5 |
| MOL007122 | Miltirone | Prostaglandin G/H synthase 2 | | PTGES3 |
| MOL007122 | Miltirone | Nitric-oxide synthase, endothelial | | KDR |
| MOL007122 | Miltirone | Carbonic anhydrase II | | CA5A |
| MOL007122 | Miltirone | Alpha-2C adrenergic receptor | | ADRA2C |
| MOL007122 | Miltirone | Muscarinic acetylcholine receptor M4 | | CHRM4 |
| MOL007122 | Miltirone | Retinoic acid receptor RXR-alpha | | RXRA |
| MOL007122 | Miltirone | Delta-type opioid receptor | | OPRD1 |
| MOL007122 | Miltirone | Alpha-1A adrenergic receptor | | ADRA1A |
| MOL007122 | Miltirone | Muscarinic acetylcholine receptor M2 | | CHRM2 |
| MOL007122 | Miltirone | Alpha-1B adrenergic receptor | | ADRA1B |
| MOL007122 | Miltirone | Sodium-dependent dopamine transporter | | SLC6A3 |
| MOL007122 | Miltirone | Beta-2 adrenergic receptor | | GRK2 |
| MOL007122 | Miltirone | Alpha-1D adrenergic receptor | | ADRA1D |
| MOL007122 | Miltirone | DNA topoisomerase II | | DHX9 |
| MOL007122 | Miltirone | Mu-type opioid receptor | | OPRM1 |
| MOL007122 | Miltirone | Neuronal acetylcholine receptor protein, alpha-7 chain | | CHRNA6 |
| MOL007122 | Miltirone | Nuclear receptor coactivator 2 | | BUD23 |
| MOL007124 | neocryptotanshinone ii | Prostaglandin G/H synthase 1 | | PTGS1 |
| MOL007124 | neocryptotanshinone ii | Dopamine D1 receptor | | DRD1 |
| MOL007124 | neocryptotanshinone ii | Muscarinic acetylcholine receptor M3 | | CHRM3 |
| MOL007124 | neocryptotanshinone ii | Muscarinic acetylcholine receptor M1 | | CHRM1 |
| MOL007124 | neocryptotanshinone ii | Estrogen receptor | | CTH |
| MOL007124 | neocryptotanshinone ii | Androgen receptor | | PKN1 |
| MOL007124 | neocryptotanshinone ii | Sodium channel protein type 5 subunit alpha | | SCNN1B |
| MOL007124 | neocryptotanshinone ii | Prostaglandin G/H synthase 2 | | PTGES3 |
| MOL007124 | neocryptotanshinone ii | Nitric-oxide synthase, endothelial | | KDR |
| MOL007124 | neocryptotanshinone ii | Carbonic anhydrase II | | CA5A |
| MOL007124 | neocryptotanshinone ii | Muscarinic acetylcholine receptor M4 | | CHRM4 |
| MOL007124 | neocryptotanshinone ii | Retinoic acid receptor RXR-alpha | | RXRA |
| MOL007124 | neocryptotanshinone ii | Delta-type opioid receptor | | OPRD1 |
| MOL007124 | neocryptotanshinone ii | CGMP-inhibited 3',5'-cyclic phosphodiesterase A | | PDE3A |
| MOL007124 | neocryptotanshinone ii | Alpha-1A adrenergic receptor | | ADRA1A |
| MOL007124 | neocryptotanshinone ii | Muscarinic acetylcholine receptor M2 | | CHRM2 |
| MOL007124 | neocryptotanshinone ii | Alpha-1B adrenergic receptor | | ADRA1B |
| MOL007124 | neocryptotanshinone ii | Sodium-dependent dopamine transporter | | SLC6A3 |
| MOL007124 | neocryptotanshinone ii | Beta-2 adrenergic receptor | | GRK2 |
| MOL007124 | neocryptotanshinone ii | Alpha-1D adrenergic receptor | | ADRA1D |
| MOL007124 | neocryptotanshinone ii | Sodium-dependent serotonin transporter | | SLC6A4 |
| MOL007124 | neocryptotanshinone ii | Mu-type opioid receptor | | OPRM1 |
| MOL007124 | neocryptotanshinone ii | Gamma-aminobutyric acid receptor subunit alpha-1 | | GABRA1 |
| MOL007124 | neocryptotanshinone ii | Glycogen synthase kinase-3 beta | | GSK3B |
| MOL007124 | neocryptotanshinone ii | Heat shock protein HSP 90 | | HSPB1 |
| MOL007124 | neocryptotanshinone ii | Cell division protein kinase 2 | | CDK2 |
| MOL007124 | neocryptotanshinone ii | Neuronal acetylcholine receptor protein, alpha-7 chain | | CHRNA6 |
| MOL007124 | neocryptotanshinone ii | Proto-oncogene serine/threonine-protein kinase Pim-1 | | PIM1 |
| MOL007124 | neocryptotanshinone ii | Cyclin-A2 | | CCNA2 |
| MOL007125 | neocryptotanshinone | Prostaglandin G/H synthase 1 | | PTGS1 |
| MOL007125 | neocryptotanshinone | Muscarinic acetylcholine receptor M3 | | CHRM3 |
| MOL007125 | neocryptotanshinone | Muscarinic acetylcholine receptor M1 | | CHRM1 |
| MOL007125 | neocryptotanshinone | Sodium channel protein type 5 subunit alpha | | SCNN1B |
| MOL007125 | neocryptotanshinone | Peroxisome proliferator activated receptor gamma | | PPARA |
| MOL007125 | neocryptotanshinone | Prostaglandin G/H synthase 2 | | PTGES3 |
| MOL007125 | neocryptotanshinone | Carbonic anhydrase II | | CA5A |
| MOL007125 | neocryptotanshinone | Alpha-1B adrenergic receptor | | ADRA1B |
| MOL007125 | neocryptotanshinone | Beta-2 adrenergic receptor | | GRK2 |
| MOL007125 | neocryptotanshinone | Alpha-1D adrenergic receptor | | ADRA1D |
| MOL007125 | neocryptotanshinone | DNA topoisomerase II | | DHX9 |
| MOL007125 | neocryptotanshinone | Mu-type opioid receptor | | OPRM1 |
| MOL007125 | neocryptotanshinone | Neuronal acetylcholine receptor protein, alpha-7 chain | | CHRNA6 |
| MOL007125 | neocryptotanshinone | Ig gamma-1 chain C region | | IGHG1 |
| MOL007125 | neocryptotanshinone | Nuclear receptor coactivator 2 | | BUD23 |
| MOL007125 | neocryptotanshinone | Nuclear receptor coactivator 1 | | BUD23 |
| MOL007127 | 1-methyl-8,9-dihydro-7H-naphtho[5,6-g]benzofuran-6,10,11-trione | Prostaglandin G/H synthase 1 | | PTGS1 |
| MOL007127 | 1-methyl-8,9-dihydro-7H-naphtho[5,6-g]benzofuran-6,10,11-trione | Dopamine D1 receptor | | DRD1 |
| MOL007127 | 1-methyl-8,9-dihydro-7H-naphtho[5,6-g]benzofuran-6,10,11-trione | Muscarinic acetylcholine receptor M3 | | CHRM3 |
| MOL007127 | 1-methyl-8,9-dihydro-7H-naphtho[5,6-g]benzofuran-6,10,11-trione | Thrombin | | CXCL8 |
| MOL007127 | 1-methyl-8,9-dihydro-7H-naphtho[5,6-g]benzofuran-6,10,11-trione | Sodium channel protein type 5 subunit alpha | | SCNN1B |
| MOL007127 | 1-methyl-8,9-dihydro-7H-naphtho[5,6-g]benzofuran-6,10,11-trione | Muscarinic acetylcholine receptor M5 | | CHRM5 |
| MOL007127 | 1-methyl-8,9-dihydro-7H-naphtho[5,6-g]benzofuran-6,10,11-trione | Prostaglandin G/H synthase 2 | | PTGES3 |
| MOL007127 | 1-methyl-8,9-dihydro-7H-naphtho[5,6-g]benzofuran-6,10,11-trione | Carbonic anhydrase II | | CA5A |
| MOL007127 | 1-methyl-8,9-dihydro-7H-naphtho[5,6-g]benzofuran-6,10,11-trione | Retinoic acid receptor RXR-alpha | | RXRA |
| MOL007127 | 1-methyl-8,9-dihydro-7H-naphtho[5,6-g]benzofuran-6,10,11-trione | Acetylcholinesterase | | ACHE |
| MOL007127 | 1-methyl-8,9-dihydro-7H-naphtho[5,6-g]benzofuran-6,10,11-trione | Alpha-1A adrenergic receptor | | ADRA1A |
| MOL007127 | 1-methyl-8,9-dihydro-7H-naphtho[5,6-g]benzofuran-6,10,11-trione | Beta-2 adrenergic receptor | | GRK2 |
| MOL007127 | 1-methyl-8,9-dihydro-7H-naphtho[5,6-g]benzofuran-6,10,11-trione | Mu-type opioid receptor | | OPRM1 |
| MOL007127 | 1-methyl-8,9-dihydro-7H-naphtho[5,6-g]benzofuran-6,10,11-trione | Gamma-aminobutyric acid receptor subunit alpha-1 | | GABRA1 |
| MOL007127 | 1-methyl-8,9-dihydro-7H-naphtho[5,6-g]benzofuran-6,10,11-trione | Dipeptidyl peptidase IV | | DPP4 |
| MOL007127 | 1-methyl-8,9-dihydro-7H-naphtho[5,6-g]benzofuran-6,10,11-trione | Heat shock protein HSP 90 | | HSPB1 |
| MOL007127 | 1-methyl-8,9-dihydro-7H-naphtho[5,6-g]benzofuran-6,10,11-trione | Phosphatidylinositol-4,5-bisphosphate 3-kinase catalytic subunit, gamma isoform | | PIK3CG |
| MOL007127 | 1-methyl-8,9-dihydro-7H-naphtho[5,6-g]benzofuran-6,10,11-trione | Neuronal acetylcholine receptor protein, alpha-7 chain | | CHRNA6 |
| MOL007127 | 1-methyl-8,9-dihydro-7H-naphtho[5,6-g]benzofuran-6,10,11-trione | Ig gamma-1 chain C region | | IGHG1 |
| MOL007127 | 1-methyl-8,9-dihydro-7H-naphtho[5,6-g]benzofuran-6,10,11-trione | Nuclear receptor coactivator 1 | | BUD23 |
| MOL007130 | prolithospermic acid | Nitric oxide synthase, inducible | | NOS2 |
| MOL007130 | prolithospermic acid | Prostaglandin G/H synthase 1 | | PTGS1 |
| MOL007130 | prolithospermic acid | Thrombin | | CXCL8 |
| MOL007130 | prolithospermic acid | Estrogen receptor | | CTH |
| MOL007130 | prolithospermic acid | Androgen receptor | | PKN1 |
| MOL007130 | prolithospermic acid | Prostaglandin G/H synthase 2 | | PTGES3 |
| MOL007130 | prolithospermic acid | mRNA of Protein-tyrosine phosphatase, non-receptor type 1 | | PTPN22 |
| MOL007130 | prolithospermic acid | Heat shock protein HSP 90 | | HSPB1 |
| MOL007130 | prolithospermic acid | Trypsin-1 | | PRSS1 |
| MOL007130 | prolithospermic acid | Calmodulin | | IQCB1 |
| MOL007132 | (2R)-3-(3,4-dihydroxyphenyl)-2-[(Z)-3-(3,4-dihydroxyphenyl)acryloyl]oxy-propionic acid | Thrombin | | CXCL8 |
| MOL007132 | (2R)-3-(3,4-dihydroxyphenyl)-2-[(Z)-3-(3,4-dihydroxyphenyl)acryloyl]oxy-propionic acid | Estrogen receptor | | CTH |
| MOL007132 | (2R)-3-(3,4-dihydroxyphenyl)-2-[(Z)-3-(3,4-dihydroxyphenyl)acryloyl]oxy-propionic acid | Androgen receptor | | PKN1 |
| MOL007132 | (2R)-3-(3,4-dihydroxyphenyl)-2-[(Z)-3-(3,4-dihydroxyphenyl)acryloyl]oxy-propionic acid | Peroxisome proliferator activated receptor gamma | | PPARA |
| MOL007132 | (2R)-3-(3,4-dihydroxyphenyl)-2-[(Z)-3-(3,4-dihydroxyphenyl)acryloyl]oxy-propionic acid | Prostaglandin G/H synthase 2 | | PTGES3 |
| MOL007132 | (2R)-3-(3,4-dihydroxyphenyl)-2-[(Z)-3-(3,4-dihydroxyphenyl)acryloyl]oxy-propionic acid | Dipeptidyl peptidase IV | | DPP4 |
| MOL007132 | (2R)-3-(3,4-dihydroxyphenyl)-2-[(Z)-3-(3,4-dihydroxyphenyl)acryloyl]oxy-propionic acid | Trypsin-1 | | PRSS1 |
| MOL007132 | (2R)-3-(3,4-dihydroxyphenyl)-2-[(Z)-3-(3,4-dihydroxyphenyl)acryloyl]oxy-propionic acid | Cyclin-A2 | | CCNA2 |
| MOL007141 | salvianolic acid g | Prostaglandin G/H synthase 2 | | PTGES3 |
| MOL007142 | salvianolic acid j | Coagulation factor VII | | F2RL1 |
| MOL007142 | salvianolic acid j | mRNA of Protein-tyrosine phosphatase, non-receptor type 1 | | PTPN22 |
| MOL007142 | salvianolic acid j | Trypsin-1 | | PRSS1 |
| MOL007143 | salvilenone Ⅰ | Prostaglandin G/H synthase 2 | | PTGES3 |
| MOL007143 | salvilenone Ⅰ | Retinoic acid receptor RXR-alpha | | RXRA |
| MOL007143 | salvilenone Ⅰ | Acetylcholinesterase | | ACHE |
| MOL007143 | salvilenone Ⅰ | Progesterone receptor | | NR3C2 |
| MOL007143 | salvilenone Ⅰ | mRNA of Protein-tyrosine phosphatase, non-receptor type 1 | | PTPN22 |
| MOL007143 | salvilenone Ⅰ | Glucocorticoid receptor | | FKBP4 |
| MOL007143 | salvilenone Ⅰ | Nuclear receptor coactivator 2 | | BUD23 |
| MOL007143 | salvilenone Ⅰ | Nuclear receptor coactivator 1 | | BUD23 |
| MOL007145 | salviolone | Prostaglandin G/H synthase 1 | | PTGS1 |
| MOL007145 | salviolone | Dopamine D1 receptor | | DRD1 |
| MOL007145 | salviolone | Muscarinic acetylcholine receptor M3 | | CHRM3 |
| MOL007145 | salviolone | Thrombin | | CXCL8 |
| MOL007145 | salviolone | Muscarinic acetylcholine receptor M1 | | CHRM1 |
| MOL007145 | salviolone | D(1B) dopamine receptor | | GABBR1 |
| MOL007145 | salviolone | Sodium channel protein type 5 subunit alpha | | SCNN1B |
| MOL007145 | salviolone | Muscarinic acetylcholine receptor M5 | | CHRM5 |
| MOL007145 | salviolone | Prostaglandin G/H synthase 2 | | PTGES3 |
| MOL007145 | salviolone | Alpha-2A adrenergic receptor | | ADRA2A |
| MOL007145 | salviolone | 5-hydroxytryptamine 1A receptor | | HTR1A |
| MOL007145 | salviolone | 5-hydroxytryptamine receptor 3A | | HTR3A |
| MOL007145 | salviolone | Gamma-aminobutyric-acid receptor alpha-2 subunit | | GABRG2 |
| MOL007145 | salviolone | Muscarinic acetylcholine receptor M4 | | CHRM4 |
| MOL007145 | salviolone | Delta-type opioid receptor | | OPRD1 |
| MOL007145 | salviolone | Acetylcholinesterase | | ACHE |
| MOL007145 | salviolone | CGMP-inhibited 3',5'-cyclic phosphodiesterase A | | PDE3A |
| MOL007145 | salviolone | 5-hydroxytryptamine 2A receptor | | HTR2A |
| MOL007145 | salviolone | Gamma-aminobutyric-acid receptor alpha-5 subunit | | GABRG2 |
| MOL007145 | salviolone | Sodium-dependent noradrenaline transporter | | SLC6A2 |
| MOL007145 | salviolone | Alpha-1A adrenergic receptor | | ADRA1A |
| MOL007145 | salviolone | Gamma-aminobutyric-acid receptor alpha-3 subunit | | GABRG2 |
| MOL007145 | salviolone | 5-hydroxytryptamine 2C receptor | | HTR2C |
| MOL007145 | salviolone | Muscarinic acetylcholine receptor M2 | | CHRM2 |
| MOL007145 | salviolone | Alpha-2B adrenergic receptor | | ADRA2B |
| MOL007145 | salviolone | Alpha-1B adrenergic receptor | | ADRA1B |
| MOL007145 | salviolone | Sodium-dependent dopamine transporter | | SLC6A3 |
| MOL007145 | salviolone | Beta-2 adrenergic receptor | | GRK2 |
| MOL007145 | salviolone | Neuronal acetylcholine receptor subunit alpha-2 | | CHRNA3 |
| MOL007145 | salviolone | Sodium-dependent serotonin transporter | | SLC6A4 |
| MOL007145 | salviolone | D(2) dopamine receptor | | SNCA |
| MOL007145 | salviolone | Mu-type opioid receptor | | OPRM1 |
| MOL007145 | salviolone | Gamma-aminobutyric acid receptor subunit alpha-1 | | GABRA1 |
| MOL007145 | salviolone | 5-hydroxytryptamine 1B receptor | | HTR1B |
| MOL007145 | salviolone | Neuronal acetylcholine receptor protein, alpha-7 chain | | CHRNA6 |
| MOL007145 | salviolone | Gamma-aminobutyric-acid receptor subunit alpha-6 | | GABRG2 |
| MOL007145 | salviolone | Gamma-aminobutyric acid receptor subunit gamma-3 | | PLCL1 |
| MOL007145 | salviolone | Gamma-aminobutyric acid receptor subunit epsilon | | GABRE |
| MOL007150 | (6S)-6-hydroxy-1-methyl-6-methylol-8,9-dihydro-7H-naphtho[8,7-g]benzofuran-10,11-quinone | Thrombin | | CXCL8 |
| MOL007150 | (6S)-6-hydroxy-1-methyl-6-methylol-8,9-dihydro-7H-naphtho[8,7-g]benzofuran-10,11-quinone | Prostaglandin G/H synthase 2 | | PTGES3 |
| MOL007150 | (6S)-6-hydroxy-1-methyl-6-methylol-8,9-dihydro-7H-naphtho[8,7-g]benzofuran-10,11-quinone | Carbonic anhydrase II | | CA5A |
| MOL007150 | (6S)-6-hydroxy-1-methyl-6-methylol-8,9-dihydro-7H-naphtho[8,7-g]benzofuran-10,11-quinone | Acetylcholinesterase | | ACHE |
| MOL007150 | (6S)-6-hydroxy-1-methyl-6-methylol-8,9-dihydro-7H-naphtho[8,7-g]benzofuran-10,11-quinone | Dipeptidyl peptidase IV | | DPP4 |
| MOL007150 | (6S)-6-hydroxy-1-methyl-6-methylol-8,9-dihydro-7H-naphtho[8,7-g]benzofuran-10,11-quinone | Heat shock protein HSP 90 | | HSPB1 |
| MOL007150 | (6S)-6-hydroxy-1-methyl-6-methylol-8,9-dihydro-7H-naphtho[8,7-g]benzofuran-10,11-quinone | Trypsin-1 | | PRSS1 |
| MOL007150 | (6S)-6-hydroxy-1-methyl-6-methylol-8,9-dihydro-7H-naphtho[8,7-g]benzofuran-10,11-quinone | Nuclear receptor coactivator 1 | | BUD23 |
| MOL007151 | Tanshindiol B | Thrombin | | CXCL8 |
| MOL007151 | Tanshindiol B | Prostaglandin G/H synthase 2 | | PTGES3 |
| MOL007151 | Tanshindiol B | Carbonic anhydrase II | | CA5A |
| MOL007151 | Tanshindiol B | Acetylcholinesterase | | ACHE |
| MOL007151 | Tanshindiol B | Dipeptidyl peptidase IV | | DPP4 |
| MOL007151 | Tanshindiol B | Heat shock protein HSP 90 | | HSPB1 |
| MOL007151 | Tanshindiol B | Nuclear receptor coactivator 1 | | BUD23 |
| MOL007152 | Przewaquinone E | Thrombin | | CXCL8 |
| MOL007152 | Przewaquinone E | Prostaglandin G/H synthase 2 | | PTGES3 |
| MOL007152 | Przewaquinone E | Carbonic anhydrase II | | CA5A |
| MOL007152 | Przewaquinone E | Acetylcholinesterase | | ACHE |
| MOL007152 | Przewaquinone E | Dipeptidyl peptidase IV | | DPP4 |
| MOL007152 | Przewaquinone E | Heat shock protein HSP 90 | | HSPB1 |
| MOL007152 | Przewaquinone E | Nuclear receptor coactivator 1 | | BUD23 |
| MOL007154 | tanshinone iia | Dopamine D1 receptor | | DRD1 |
| MOL007154 | tanshinone iia | Muscarinic acetylcholine receptor M3 | | CHRM3 |
| MOL007154 | tanshinone iia | Thrombin | | CXCL8 |
| MOL007154 | tanshinone iia | Muscarinic acetylcholine receptor M1 | | CHRM1 |
| MOL007154 | tanshinone iia | Sodium channel protein type 5 subunit alpha | | SCNN1B |
| MOL007154 | tanshinone iia | Muscarinic acetylcholine receptor M5 | | CHRM5 |
| MOL007154 | tanshinone iia | Prostaglandin G/H synthase 2 | | PTGES3 |
| MOL007154 | tanshinone iia | Muscarinic acetylcholine receptor M4 | | CHRM4 |
| MOL007154 | tanshinone iia | Delta-type opioid receptor | | OPRD1 |
| MOL007154 | tanshinone iia | Acetylcholinesterase | | ACHE |
| MOL007154 | tanshinone iia | Alpha-1A adrenergic receptor | | ADRA1A |
| MOL007154 | tanshinone iia | Muscarinic acetylcholine receptor M2 | | CHRM2 |
| MOL007154 | tanshinone iia | Beta-2 adrenergic receptor | | GRK2 |
| MOL007154 | tanshinone iia | Mu-type opioid receptor | | OPRM1 |
| MOL007154 | tanshinone iia | Dipeptidyl peptidase IV | | DPP4 |
| MOL007154 | tanshinone iia | Neuronal acetylcholine receptor protein, alpha-7 chain | | CHRNA6 |
| MOL007154 | tanshinone iia | Nuclear receptor coactivator 1 | | BUD23 |
| MOL007154 | tanshinone iia | Retinoic acid receptor RXR-alpha | | RXRA |
| MOL007154 | tanshinone iia | Transcription factor p65 | | CDK9 |
| MOL007154 | tanshinone iia | Apoptosis regulator Bcl-2 | | BCL2L1 |
| MOL007154 | tanshinone iia | Proto-oncogene c-Fos | | FOS |
| MOL007154 | tanshinone iia | Cyclin-dependent kinase inhibitor 1 | | CDK9 |
| MOL007154 | tanshinone iia | Matrix metalloproteinase-9 | | MMP9 |
| MOL007154 | tanshinone iia | Transcription factor AP-1 | | ESR1 |
| MOL007154 | tanshinone iia | Activator of 90 kDa heat shock protein ATPase homolog 1 | | AHSA1 |
| MOL007154 | tanshinone iia | Caspase-3 | | HTT |
| MOL007154 | tanshinone iia | Cellular tumor antigen p53 | | TP53 |
| MOL007154 | tanshinone iia | NF-kappa-B inhibitor alpha | | ARRB2 |
| MOL007154 | tanshinone iia | Fatty acid synthase | | PTGES3 |
| MOL007154 | tanshinone iia | Endothelin-1 receptor | | EDN1 |
| MOL007154 | tanshinone iia | Endothelin-1 | | EDN1 |
| MOL007154 | tanshinone iia | Cytochrome P450 3A4 | | CYP3A4 |
| MOL007154 | tanshinone iia | Cytochrome P450 1A2 | | CYP1A2 |
| MOL007154 | tanshinone iia | Myc proto-oncogene protein | | NPM1 |
| MOL007154 | tanshinone iia | Cytochrome P450 1A1 | | CYP1A1 |
| MOL007154 | tanshinone iia | Nuclear receptor subfamily 1 group I member 2 | | NR3C2 |
| MOL007154 | tanshinone iia | Nucleophosmin | | NPM1 |
| MOL007154 | tanshinone iia | Endothelin-converting enzyme 1 | | ECE1 |
| MOL007154 | tanshinone iia | Calcitonin receptor | | CALCA |
| MOL007154 | tanshinone iia | Integrin beta-3 | | CCN1 |
| MOL007155 | (6S)-6-(hydroxymethyl)-1,6-dimethyl-8,9-dihydro-7H-naphtho[8,7-g]benzofuran-10,11-dione | Thrombin | | CXCL8 |
| MOL007155 | (6S)-6-(hydroxymethyl)-1,6-dimethyl-8,9-dihydro-7H-naphtho[8,7-g]benzofuran-10,11-dione | Muscarinic acetylcholine receptor M1 | | CHRM1 |
| MOL007155 | (6S)-6-(hydroxymethyl)-1,6-dimethyl-8,9-dihydro-7H-naphtho[8,7-g]benzofuran-10,11-dione | Sodium channel protein type 5 subunit alpha | | SCNN1B |
| MOL007155 | (6S)-6-(hydroxymethyl)-1,6-dimethyl-8,9-dihydro-7H-naphtho[8,7-g]benzofuran-10,11-dione | Prostaglandin G/H synthase 2 | | PTGES3 |
| MOL007155 | (6S)-6-(hydroxymethyl)-1,6-dimethyl-8,9-dihydro-7H-naphtho[8,7-g]benzofuran-10,11-dione | Delta-type opioid receptor | | OPRD1 |
| MOL007155 | (6S)-6-(hydroxymethyl)-1,6-dimethyl-8,9-dihydro-7H-naphtho[8,7-g]benzofuran-10,11-dione | Acetylcholinesterase | | ACHE |
| MOL007155 | (6S)-6-(hydroxymethyl)-1,6-dimethyl-8,9-dihydro-7H-naphtho[8,7-g]benzofuran-10,11-dione | Alpha-1A adrenergic receptor | | ADRA1A |
| MOL007155 | (6S)-6-(hydroxymethyl)-1,6-dimethyl-8,9-dihydro-7H-naphtho[8,7-g]benzofuran-10,11-dione | Beta-2 adrenergic receptor | | GRK2 |
| MOL007155 | (6S)-6-(hydroxymethyl)-1,6-dimethyl-8,9-dihydro-7H-naphtho[8,7-g]benzofuran-10,11-dione | Mu-type opioid receptor | | OPRM1 |
| MOL007155 | (6S)-6-(hydroxymethyl)-1,6-dimethyl-8,9-dihydro-7H-naphtho[8,7-g]benzofuran-10,11-dione | Dipeptidyl peptidase IV | | DPP4 |
| MOL007155 | (6S)-6-(hydroxymethyl)-1,6-dimethyl-8,9-dihydro-7H-naphtho[8,7-g]benzofuran-10,11-dione | Neuronal acetylcholine receptor protein, alpha-7 chain | | CHRNA6 |
| MOL007155 | (6S)-6-(hydroxymethyl)-1,6-dimethyl-8,9-dihydro-7H-naphtho[8,7-g]benzofuran-10,11-dione | Trypsin-1 | | PRSS1 |
| MOL007155 | (6S)-6-(hydroxymethyl)-1,6-dimethyl-8,9-dihydro-7H-naphtho[8,7-g]benzofuran-10,11-dione | Nuclear receptor coactivator 1 | | BUD23 |
| MOL007156 | tanshinone Ⅵ | Prostaglandin G/H synthase 1 | | PTGS1 |
| MOL007156 | tanshinone Ⅵ | Estrogen receptor | | CTH |
| MOL007156 | tanshinone Ⅵ | Androgen receptor | | PKN1 |
| MOL007156 | tanshinone Ⅵ | Sodium channel protein type 5 subunit alpha | | SCNN1B |
| MOL007156 | tanshinone Ⅵ | Peroxisome proliferator activated receptor gamma | | PPARA |
| MOL007156 | tanshinone Ⅵ | Coagulation factor Xa | | F2RL1 |
| MOL007156 | tanshinone Ⅵ | Prostaglandin G/H synthase 2 | | PTGES3 |
| MOL007156 | tanshinone Ⅵ | Heat shock protein HSP 90 | | HSPB1 |
| MOL007156 | tanshinone Ⅵ | Beta-lactamase | | COA7 |
| MOL007156 | tanshinone Ⅵ | Ig gamma-1 chain C region | | IGHG1 |
| MOL007156 | tanshinone Ⅵ | Nuclear receptor coactivator 2 | | BUD23 |
| MOL007156 | tanshinone Ⅵ | Nuclear receptor coactivator 1 | | BUD23 |
| MOL007156 | tanshinone Ⅵ | Calmodulin | | IQCB1 |

Supplementary table 4. Potential targets of COVID-19

| Disease | Targets |
| --- | --- |
| COVID-19 | ACE2,TNF,CRP,IL6,CXCL8,IL10,IFNG,IL1B,CCL2,TLR4,IL4,ACE,CXCL10,MBL2,IL17A,ELANE,ALB,CCL3,SFTPD,EGFR,HLAA,CSF3,CD40LG,CCL5,TLR7,TLR2,TMPRSS2,STAT3,IL18,CD8A,IFNB1,CD4,CSF2,IFIH1,GPT,ADA,DPP4,TLR5,F3,MASP2,IL2,HMGB1,TLR3,IL13,SERPINA1,HMOX1,IRF3,TGFB1,IL5,NLRP3,IFNA1,NFKB1,MUC1,HLAB,CCR6,CTSB,TP53,NEU1,IL7,F2,FURIN,MYD88,CTSL,BSG,IL22,CXCL1,HLAC,DEFB4A,TYK2,CCL11,DHFR,AVP,TREM1,IL2RA,FCGR2A,CXCL2,PIK3C2A,PLG,CLEC4M,HIF1A,VEGFA,NPPB,PRF1,AGTR2,INS,AGTR1,PPARG,CXCL5,RIGI,TNFRSF1A,CREBBP,CD79A,C5AR1,REN,MMP12,PIK3CD,NOS3,HLADRB1,SERPINA3,TNNI3,SERPINE1,MIR223,HSPA5,ICAM1,NCF2,CTNNB1,CASP3,TBK1,NRP1,BCL2,CD209,HLADQB1,MAPK1,STING1,MTOR,PPP1CA,ANPEP,CD274,CD14,ICOSLG,MAPK3,MIR146A,SMPD1,JUN,ADAM17,AGT,MIR21,RELA,NPC2,AKT1,IKBKG,MMP7,IGF1,ISG15,KNG1,PURA,FBN1,CXCL9,SH2D3A,CASP8,SLC6A19,PKD1,IL1A,PIK3R1,TOLLIP,VWF,FGF2,TRIM21,EP300,NKX2-5,EEF1A1,IL17RA,CALCA,VDR,IRF5,MAPK8,MAVS,IFNA2,MIR155,AR,NFE2L2,STAT1,CXCR3,RNASE3,TRAF3,FCGR3B,NBN,PSEN1,ITGB2,SIRT1,MAPK14,TNFRSF1B,PARP1,POLA1,NPC1,IL33,SRP54,SREBF1,SNCA,GAPDH,APP,FCGR3A,PTGS2,ITGB1,RHOA,H2AC18,MTHFD1,IFNAR1,SBDS,SERPINC1,SPP1,TLR6,TMPRSS11D,LTF,CCL4,CCR5,HSP90AA1,IL2RB,MMP1,TMPRSS4,BMAL1,PLOD1,HFE,SOD1,SERPING1,PPIA,CAV1,ALOX5,F5,TOP1,CHI3L1,TLR9,AGER,HAX1,FLNA,CD3D,UBC,CANX,PLAUR,CCND1,LEP,GSTM1,CD81,IRF9,JAK1,AP3B1,MDM2,PLAT,KIT,UBB,C1QBP,HDAC2,HP,ZIC2,MMP3,RAB7A,TRAF6,S100A8,LMNB1,CGAS,TTR,CREB1,GIPC1,APOE,EPRS1,FBLN5,G6PD,ANXA5,TK2,LDLR,SMAD3,GSN,YWHAE,EDN1,BMP6,BAX,VTN,IGKC,NAGLU,AASS,LOC112679198,CASP1,PTX3,LGALS3,SMC1A,LOX,CLEC4G,MCL1,OFD1,SRP19,TF,GATA6,PRL,EIF2AK2,ACTB,PDHA1,OAS1,VIM,SIGLEC5,CD34,COPA,CD3E,SELL,IKBKB,SMAD2,VCP,GNAS,TBC1D24,WDR1,LOC117134593,MX1,SCN5A,GLA,BDKRB1,KPNA2,EXOSC10,CARD9,IRF7,MSH2,TCF3,TIMM8A,ITGAL,CAMP,SMAD4,SERPINH1,LOC117134604,TRIM25,HSPA4,OGA,TFG,LONP1,CASP6,VDAC1,SMARCB1,SMAD7,BLM,RB1,ENO1,FASN,CAT,MIRLET7A1,NLRP12,NFKBIA,THBD,PIK3CA,SSB,BCL2L1,NUP98,F8,CDK2,DCTN1,LOC117134608,IFITM3,CCR3,PABPN1,ATP7B,ATP6V1B2,ANO6,LOC117135106,S100A9,STAT2,IGHMBP2,HSPB1,CP,ANXA2,RAB1A,EGR1,RAB5A,HSPA8,NRP2,PABPC4,LCN2,RAB8A,DDR1,DNM2,LOC117134605,LOC117134606,IDO1,ARF1,SCARB1,CCR1,EPHX1,STAT6,MAPKAPK2,HSP90B1,TFRC,GSK3B,OCRL,CHEK2,BECN1,SRPRA,JAK2,NOS2,VPS39,RIPK1,PRKRA,CST3,CDK4,CEACAM3,CDK5RAP2,MAP3K7,BRD4,LMAN1,PPIG,TRAF2,HDAC6,CYB5R3,VHL,SRP72,PIKFYVE,IKBKE,ADAR,PHB2,CALR,DEFA1,EIF2AK3,PTCD3,DDIT3,TERF1,S100A4,HTRA1,MIR29A,RPS27A,PALS1,NPM1,TXN,SMN1,ITGA5,SLC17A5,APOA1,IL6R,PRKCA,DUSP1,TOMM70,PLA2G4A,IFNAR2,SNAP29,COMT,CBL,TICAM1,RANBP2,SOD3,SQSTM1,MALAT1,CEACAM5,MAGT1,FOS,CD163,MIR323A,HTT,PRKCQ,CCNA2,RIPK3,DICER1,PIK3CG,GRB2,ITGB3,STUB1,NAMPT,CEACAM1,GZMA,RPL22,PPT1,TGFBI,CDK5,HYOU1,IGF2R,G3BP1,PIK3C3,RAP1A,SOAT1,GDF15,RAB1B,BAK1,PML,NEAT1,CXCL11,PRKDC,SDC1,FANCD2,CH25H,AHR,LAS1L,H2AX,LCK,NSD2,CDK1,POU5F1,PYCARD,RBX1,TPCN2,CLU,ERN1,SOCS3,HELLS,PTPN1,AICDA,HLAG,F10,P2RX7,TOR1A,RAE1,AAK1,DNAJB1,ACADM,EZR,HSPA1A,BST2,XRCC5,ILK,COL6A1,TLL1,IRAK3,TLR10,IL12B,NIN,VKORC1,POLR2A,ANGPT2,ADNP,P4HB,DNMT1,BAG6,PRKCE,THPO,PROCR,IL37,ACAD9,CLEC12A,CIITA,PARK7,DYNC1H1,DCTN2,MTHFR,LY6E,GSTT1,RUNX1,LGALS9,ABCB7,PRKAR1A,EIF2S1,BAG3,STIM1,PPP1R12C,CSNK2B,TPM1,UPF1,RAD50,POLR1C,WDR26,LGALS1,HAVCR2,ATF6,KPNB1,CCDC22,PCNT,NEK9,FHL2,ATF2,HBA1,FMR1,F11,POR,SDCBP,KPNA1,XBP1,PSMC4,ARF6,YWHAQ,NT5E,YARS1,BAP1,ABCC1,ITGAV,MSH6,BAD,CCL7,MYO18A,GIGYF2,PIK3CB,CEP250,SLC19A2,IDE,DDX3X,AXL,PABPC1,SOCS1,ELOC,CEP290,NEFL,ATF4,ZMPSTE24,ACADL,CYP51A1,TKT,UBE3A,TRIP4,CCHCR1,AHCY,CENPF,ABO,VAPA,HADHA,DYM,TNFSF14,FCER2,SIL1,DDB1,RTN4,MKRN3,ELOB,GNE,CREG1,CSNK2A2,PRKACA,IMPDH2,CSNK2A1,PLOD2,YY1,RPLP2,CEP131,MRE11,RPS20,RDX,SUMO1,NME4,RAB27A,MMUT,VAMP8,AKAP9,PSMD1,PPIB,CTSK,MIR145,USP13,CLCN7,IFNL1,FYCO1,EZH2,RAPGEF3,C9orf72,KLRK1,G3BP2,RPS3,EMD,KPNA6,PKP2,FBN2,KPNA3,ERLIN2,CASP4,TBKBP1,CLTC,MFGE8,ADAMTS13,KRT8,RPS27,GGT1,HAVCR1,XPO1,MIR200C,APOD,AGL,PLA2G7,CA2,COPG1,NPTX1,IL16,KEAP1,ETFA,DNAJC11,NEDD4,EIF4E2,C1R,CUL1,SNAPIN,DNAJB4,RNPC3,IRF1,SF3B1,MIR148A,RNF5,NUP88,UMPS,PRKAR2A,GSK3A,RPA1,ENPEP,IFITM1,NUP62,MOV10,NLRP1,TM9SF2,PRG2,EIF3A,GC,VPS11,SPTAN1,PRDX5,EXOSC3,HLAE,TNFAIP3,GPHN,KPNA4,NUP93,AKAP8L,ZBTB16,PEX1,ZC3HAV1,ADAM10,TYSND1,GNB1,CTRL,PCSK6,NUP214,KRT19,BRD2,ADAM9,STK4,ADSL,FH,EIF4E,ATP6AP1,EXOSC5,ERP44,CUL2,GOLGB1,F2RL1,TOR1AIP1,PSMC6,ANXA1,LOC118966792,CAPN1,FKBP1A,TIRAP,GOLGA2,PSMD11,PSMD8,BCKDHA,ALDH7A1,DDX21,FARSA,HECTD1,CHGA,GARS1,SMOC1,ERC1,USP14,RPS17,USP10,ORAI1,STOM,PSME3,HOOK1,EIF3F,HPSE,NR2F2,LMAN2,DNAJC19,EEF2,MATR3,LAG3,ACACA,SUN2,IFITM2,PRSS2,AATF,TNPO3,NGLY1,AMFR,RAN,RPS26,RALA,SPART,SAMHD1,ATP6AP2,EIF3M,FTL,RPL5,TPM4,MOGS,CHD4,TBX21,FUT3,DCAF7,PITRM1,AP2M1,RFC2,DDX6,FNDC5,EXOSC2,MAT2B,UBAP2L,MYCBP2,PIGT,FABP2,CNR2,INTS4,UBQLN1,DDAH2,NDE1,EIF2B1,PRMT1,SELENOS,EIF3E,NPHP3,CKB,AAR2,NEDD8,ICAM2,RAB5C,STMN1,UCK2,STC2,FLVCR1,YIF1A,ATP6V1A,CEP43,NXF1,SIGMAR1,VDAC2,TIMM29,TBC1D4,H2AC20,DNAJA1,RPS10,ERVW1,MSN,EIF3C,MRPS2,GGCX,NNT,BCKDK,NDUFAF2,MYOM2,JPH4,ICAM3,ATP5MG,TMPO,TJP1,AKAP8,RAB18,LPA,CRKL,IDH2,KAT2B,CHPF,TCF12,RAB10,NCSTN,INPPL1,PDE4DIP,RPAP3,POLR2B,LARP1,SLC30A9,UCHL1,ASCC1,VPS35,LIG1,TRIM32,ERO1A,ESM1,GANAB,DROSHA,BZW2,EIF3L,EIF3B,ZDHHC8,FKBP10,RAB14,AGPS,EIF4H,MTCH1,CCND3,SSX2IP,RELB,SCCPDH |

Supplementary table 5. Common Targets of COVID-19 and Danshen

| Common Targets |
| --- |
| HSPB1, SNCA, CXCL8, DPP4, F2RL1, PIK3CG, VEGFA, CCND3, BAK1, MAPK14, TNF, IL6R, HTT, TP53, TOP1, APP, MMP1, MCL1, IL2, IL4, CD40LG, CDK2, CCNA2, NOS2, GSK3B, EDN1, CDK1, BCL2L1, FOS, NPM1, CALCA |

Supplementary table 6. KEGG Pathways and related genes

| KEGG Pathways | Gene |
| --- | --- |
| Pathways in cancer | BAK1 BCL2L1 CCNA2 CCND3 CDK2 EDN1 FOS GSK3B IL2 IL4 IL6R CXCL8 MMP1 NOS2 TP53 VEGFA |
| PI3K-Akt signaling pathway | BCL2L1 CCND3 CDK2 GSK3B IL2 IL4 IL6R MCL1 PIK3CG TP53 VEGFA |
| Lipid and atherosclerosis | BCL2L1 CCND3 IL2 IL4 IL6R MCL1 |
| Measles | BCL2L1 CD40LG MAPK14 FOS GSK3B CXCL8 MMP1 TNF TP53 |
| IL-17 signaling pathway | MAPK14 FOS GSK3B IL4 CXCL8 MMP1 TNF |
| T cell receptor signaling pathway | CD40LG MAPK14 FOS GSK3B IL2 IL4 TNF |
| Kaposi sarcoma-associated herpesvirus infection | BAK1 MAPK14 FOS GSK3B CXCL8 PIK3CG TP53 VEGFA |
| Human T-cell leukemia virus 1 infection | BAK1 MAPK14 GSK3B IL6R CXCL8 TNF TP53 VEGFA |
| Human cytomegalovirus infection | CCNA2 CDK2 MAPK14 FOS CXCL8 TNF TP53 |
| Cellular senescence | MAPK14 FOS IL2 CXCL8 NOS2 TNF |
| Hepatitis B | MAPK14 FOS IL2 IL6R CXCL8 MMP1 TNF |
| Chagas disease | MAPK14 FOS GSK3B IL2 CXCL8 TNF |
| Pathways of neurodegeneration - multiple diseases | MAPK14 EDN1 FOS TNF TP53 VEGFA |
| Epstein-Barr virus infection | MAPK14 FOS GSK3B IL6R CXCL8 TNF |
| Human papillomavirus infection | MAPK14 FOS CXCL8 NOS2 TNF |
| Toxoplasmosis | MAPK14 FOS IL4 NOS2 TNF |
| Cell cycle | MAPK14 EDN1 CXCL8 TNF VEGFA |
| Coronavirus disease - COVID-19 | MAPK14 FOS IL2 IL4 IL6R |
| Relaxin signaling pathway | BCL2L1 MAPK14 GSK3B CXCL8 TNF TP53 |
| Apoptosis | BAK1 MAPK14 FOS CXCL8 PIK3CG TNF |
| Yersinia infection | MAPK14 FOS HSPB1 TNF TP53 VEGFA |
| Fluid shear stress and atherosclerosis | BAK1 MAPK14 FOS CXCL8 TNF |
| p53 signaling pathway | MAPK14 FOS IL2 IL4 |
| Non-alcoholic fatty liver disease | MAPK14 FOS CXCL8 TNF |
| Pertussis | MAPK14 EDN1 FOS TNF |
| Leishmaniasis | MAPK14 GSK3B CXCL8 TNF |
| JAK-STAT signaling pathway | MAPK14 HSPB1 VEGFA |
| Small cell lung cancer | BCL2L1 MAPK14 CXCL8 TNF |
| Rheumatoid arthritis | MAPK14 IL4 TNF |
| AGE-RAGE signaling pathway in diabetic complications | MAPK14 CXCL8 TNF |
| Allograft rejection | MAPK14 FOS GSK3B |
| Viral carcinogenesis | MAPK14 TNF TP53 VEGFA |
| Human immunodeficiency virus 1 infection | MAPK14 FOS TP53 |
| Bladder cancer | MAPK14 IL2 TNF |
| Th17 cell differentiation | MAPK14 TNF TP53 |
| Shigellosis | MAPK14 GSK3B TP53 |
| Salmonella infection | MAPK14 FOS GSK3B |
| MAPK signaling pathway | MAPK14 FOS TNF |
| Hepatitis C | MAPK14 FOS GSK3B |
| Cytokine-cytokine receptor interaction | MAPK14 FOS VEGFA |
| Pancreatic cancer | MAPK14 GSK3B TNF |
| EGFR tyrosine kinase inhibitor resistance | BAK1 BCL2L1 CCND3 CDK2 FOS GSK3B IL2 TP53 |
| Transcriptional misregulation in cancer | BCL2L1 CCNA2 CCND3 CDK2 FOS IL2 TNF TP53 |
| Colorectal cancer | CCNA2 CCND3 CDK1 CDK2 MAPK14 CXCL8 TP53 |
| Pathogenic Escherichia coli infection | BAK1 CCNA2 CCND3 CDK2 MAPK14 TNF TP53 |
| Th1 and Th2 cell differentiation | BAK1 CCNA2 CCND3 CDK2 GSK3B TNF TP53 VEGFA |
| Viral protein interaction with cytokine and cytokine receptor | CCNA2 CCND3 CDK1 CDK2 GSK3B TP53 |
| Progesterone-mediated oocyte maturation | BCL2L1 CCND3 CDK1 CDK2 TP53 |
| Amoebiasis | BAK1 CCNA2 CCND3 CDK1 CDK2 TP53 |
| Toll-like receptor signaling pathway | CCNA2 CDK1 CDK2 MAPK14 |
| NF-kappa B signaling pathway | CDK1 CDK2 MAPK14 |
| Asthma | APP BAK1 BCL2L1 MAPK14 GSK3B HTT NOS2 SNCA TNF |
| HIF-1 signaling pathway | BCL2L1 CD40LG MAPK14 NOS2 PIK3CG TNF |
| TNF signaling pathway | BCL2L1 MAPK14 NOS2 TNF TP53 |
| Alcoholic liver disease | APP GSK3B NOS2 SNCA TNF |
| Breast cancer | MAPK14 NOS2 TNF |
| Intestinal immune network for IgA production | MAPK14 EDN1 FOS MMP1 NOS2 VEGFA |
| Gastric cancer | EDN1 IL6R NOS2 VEGFA |
| Malaria | BAK1 BCL2L1 FOS MCL1 TNF TP53 |
| Autoimmune thyroid disease | BAK1 BCL2L1 CDK2 NOS2 TP53 |
| Hepatocellular carcinoma | BAK1 BCL2L1 CDK1 MAPK14 FOS TNF |
| Endometrial cancer | BAK1 CDK2 GSK3B TNF TP53 |
| Influenza A | BAK1 BCL2L1 TP53 VEGFA |
| VEGF signaling pathway | BAK1 BCL2L1 CCNA2 CXCL8 TP53 |
| Basal cell carcinoma | BAK1 FOS GSK3B TP53 |
| Amyotrophic lateral sclerosis | BAK1 FOS GSK3B TP53 |
| NOD-like receptor signaling pathway | BAK1 CDK2 GSK3B TP53 |
| Inflammatory bowel disease | BAK1 BCL2L1 GSK3B TP53 |
| Fc epsilon RI signaling pathway | BAK1 GSK3B TP53 |
| Alzheimer disease | BAK1 GSK3B TP53 |
| RIG-I-like receptor signaling pathway | BAK1 BCL2L1 TP53 |
| Prolactin signaling pathway | BAK1 BCL2L1 TP53 |
| Platinum drug resistance | CDK2 GSK3B TP53 |
| Proteoglycans in cancer | BAK1 MCL1 TP53 VEGFA |
| Chronic myeloid leukemia | BAK1 BCL2L1 TNF TP53 |
| Chemical carcinogenesis - receptor activation | BCL2L1 SNCA TP53 |
| Prostate cancer | FOS CXCL8 MMP1 TNF VEGFA |
| Endocrine resistance | CXCL8 MMP1 TP53 VEGFA |
| Hematopoietic cell lineage | CD40LG IL2 IL4 TNF |
| C-type lectin receptor signaling pathway | CD40LG IL2 IL4 IL6R CXCL8 TNF |
| Sphingolipid signaling pathway | IL2 IL6R CXCL8 TNF |
| Neurotrophin signaling pathway | CD40LG IL4 TNF |
| Growth hormone synthesis, secretion and action | CD40LG IL2 IL4 |
| MicroRNAs in cancer | CD40LG IL2 IL4 |
| Osteoclast differentiation | IL2 IL4 TNF |
| Oocyte meiosis | IL4 IL6R TNF |
| Dopaminergic synapse | BCL2L1 GSK3B IL6R VEGFA |
| Wnt signaling pathway | CCND3 GSK3B TP53 |
| Tuberculosis | CCND3 GSK3B VEGFA |
| Chemokine signaling pathway | HSPB1 CXCL8 NOS2 TNF |
| Focal adhesion | BCL2L1 CD40LG CXCL8 TNF |
| Chemical carcinogenesis - reactive oxygen species | CD40LG CXCL8 TNF |
| Herpes simplex virus 1 infection | BAK1 CCND3 CXCL8 TNF |
| Parkinson disease | CCND3 FOS VEGFA PTGES3 |
| Prion disease | GSK3B CXCL8 PIK3CG |
| Neuroactive ligand-receptor interaction | CALCA EDN1 F2RL1 |

Supplementary table 7. Go terms and related genes

| GO terms | Description | | Gene |
| --- | --- | --- | --- |
| BP | cell activation | APP BAK1 CCND3 CD40LG MAPK14 DPP4 EDN1 F2RL1 HSPB1 IL2 IL4 IL6R CXCL8 PIK3CG SNCA TNF TP53 | |
|  | leukocyte activation | APP BAK1 CCND3 CD40LG DPP4 EDN1 F2RL1 IL2 IL4 IL6R CXCL8 PIK3CG SNCA TNF TP53 | |
|  | cell activation involved in immune response | APP CD40LG F2RL1 IL2 IL4 IL6R PIK3CG TP53 | |
|  | lymphocyte activation | BAK1 CCND3 CD40LG DPP4 F2RL1 IL2 IL4 IL6R PIK3CG TP53 | |
|  | Cell population proliferation | BAK1 BCL2L1 CCND3 CD40LG CDK1 F2RL1 FOS IL2 PIK3CG TNF TP53 | |
|  | leukocyte proliferation | BCL2L1 CCND3 CD40LG F2RL1 IL2 PIK3CG TP53 | |
|  | T cell activation | CCND3 DPP4 F2RL1 IL2 IL4 IL6R PIK3CG TP53 | |
|  | leukocyte activation involved in immune response | CD40LG F2RL1 IL2 IL4 IL6R PIK3CG TP53 | |
|  | mononuclear cell proliferation | BCL2L1 CCND3 CD40LG IL2 PIK3CG TP53 | |
|  | immune effector process | APP CD40LG F2RL1 IL2 IL4 IL6R PIK3CG TP53 | |
|  | lymphocyte proliferation | CCND3 CD40LG IL2 PIK3CG TP53 | |
|  | lymphocyte activation involved in immune response | CD40LG F2RL1 IL4 IL6R TP53 | |
|  | lymphocyte differentiation | BAK1 CD40LG IL2 IL4 IL6R TP53 | |
|  | T cell proliferation | CCND3 IL2 PIK3CG TP53 | |
|  | T cell activation involved in immune response | F2RL1 IL4 IL6R TP53 | |
|  | T cell differentiation | IL2 IL4 IL6R TP53 | |
|  | adaptive immune response | CD40LG IL2 IL6R PIK3CG | |
|  | regulation of kinase activity | APP CALCA CCNA2 CCND3 CD40LG EDN1 HTT HSPB1 IL2 IL4 IL6R NPM1  PIK3CG SNCA TNF VEGFA | |
|  | regulation of protein kinase activity | APP CALCA CCNA2 CCND3 CD40LG EDN1 HTT HSPB1 IL4 IL6R NPM1 PIK3CG  SNCA TNF VEGFA | |
|  | positive regulation of protein phosphorylation | APP CALCA CCND3 CD40LG EDN1 IL2 IL4 IL6R NPM1 PIK3CG SNCA TNF TP53  VEGFA | |
|  | positive regulation of kinase activity | CALCA CCND3 CD40LG EDN1 IL2 IL4 IL6R NPM1 PIK3CG SNCA TNF VEGFA | |
|  | positive regulation of protein kinase activity | CALCA CCND3 CD40LG EDN1 IL4 IL6R NPM1 PIK3CG SNCA TNF VEGFA | |
|  | positive regulation of transferase activity | CALCA CCND3 CD40LG EDN1 IL2 IL4 IL6R NPM1 PIK3CG SNCA TNF VEGFA | |
|  | regulation of protein serine/threonine kinase activity | CCNA2 CCND3 CD40LG EDN1 HTT PIK3CG SNCA TNF VEGFA | |
|  | positive regulation of protein serine/threonine kinase activity | CCND3 CD40LG EDN1 PIK3CG SNCA TNF VEGFA | |
|  | leukocyte differentiation | APP BAK1 CD40LG MAPK14 F2RL1 FOS IL2 IL4 IL6R TNF TP53 VEGFA | |
|  | inflammatory response | APP CALCA CD40LG F2RL1 FOS IL4 IL6R CXCL8 NOS2 PIK3CG SNCA TNF | |
|  | hemopoiesis | APP BAK1 CD40LG MAPK14 F2RL1 FOS IL2 IL4 IL6R TNF TP53 VEGFA | |
|  | positive regulation of cytokine production | APP CD40LG MAPK14 F2RL1 HSPB1 IL2 IL4 IL6R NOS2 PIK3CG TNF | |
|  | hematopoietic or lymphoid organ development | APP BAK1 CD40LG MAPK14 F2RL1 FOS IL2 IL4 IL6R TNF TP53 VEGFA | |
|  | immune system development | APP BAK1 CD40LG MAPK14 F2RL1 FOS IL2 IL4 IL6R TNF TP53 VEGFA | |
|  | mononuclear cell differentiation | BAK1 CD40LG F2RL1 FOS IL2 IL4 IL6R TP53 VEGFA | |
|  | myeloid leukocyte differentiation | APP MAPK14 F2RL1 FOS IL4 TNF VEGFA | |
|  | myeloid cell differentiation | APP MAPK14 F2RL1 FOS IL4 TNF VEGFA | |
|  | positive regulation of response to external stimulus | APP EDN1 F2RL1 HSPB1 IL2 IL4 IL6R CXCL8 PIK3CG SNCA TNF VEGFA | |
|  | regulation of leukocyte migration | APP DPP4 EDN1 F2RL1 IL4 IL6R CXCL8 TNF VEGFA | |
|  | positive regulation of leukocyte migration | APP EDN1 F2RL1 IL4 IL6R CXCL8 TNF VEGFA | |
|  | regulation of leukocyte chemotaxis | DPP4 EDN1 F2RL1 IL4 IL6R CXCL8 VEGFA | |
|  | regulation of chemotaxis | DPP4 EDN1 F2RL1 HSPB1 IL4 IL6R CXCL8 VEGFA | |
|  | positive regulation of chemotaxis | EDN1 F2RL1 HSPB1 IL4 IL6R CXCL8 VEGFA | |
|  | positive regulation of cell migration | APP EDN1 F2RL1 HSPB1 IL4 IL6R CXCL8 PIK3CG TNF VEGFA | |
|  | positive regulation of cell motility | APP EDN1 F2RL1 HSPB1 IL4 IL6R CXCL8 PIK3CG TNF VEGFA | |
|  | positive regulation of locomotion | APP EDN1 F2RL1 HSPB1 IL4 IL6R CXCL8 PIK3CG TNF VEGFA | |
|  | positive regulation of leukocyte chemotaxis | EDN1 F2RL1 IL4 IL6R CXCL8 VEGFA | |
|  | chemotaxis | APP CALCA MAPK14 DPP4 EDN1 IL6R CXCL8 PIK3CG VEGFA | |
|  | taxis | APP CALCA MAPK14 DPP4 EDN1 IL6R CXCL8 PIK3CG VEGFA | |
|  | locomotion | APP CALCA MAPK14 DPP4 EDN1 IL6R CXCL8 PIK3CG VEGFA | |
|  | leukocyte migration | CALCA F2RL1 IL6R CXCL8 PIK3CG TNF | |
|  | cell chemotaxis | CALCA IL6R CXCL8 PIK3CG VEGFA | |
|  | blood vessel morphogenesis | BAK1 MAPK14 EDN1 CXCL8 PIK3CG VEGFA | |
|  | blood vessel development | BAK1 MAPK14 EDN1 CXCL8 PIK3CG VEGFA | |
|  | myeloid leukocyte migration | CALCA IL6R CXCL8 PIK3CG | |
|  | vasculature development | BAK1 MAPK14 EDN1 CXCL8 PIK3CG VEGFA | |
|  | leukocyte chemotaxis | CALCA IL6R CXCL8 PIK3CG | |
|  | angiogenesis | MAPK14 EDN1 CXCL8 PIK3CG VEGFA | |
|  | tube morphogenesis | BAK1 MAPK14 EDN1 CXCL8 PIK3CG VEGFA | |
|  | mononuclear cell migration | CALCA IL6R PIK3CG | |
|  | extrinsic apoptotic signaling pathway | BAK1 BCL2L1 GSK3B IL2 IL4 IL6R MCL1 TNF | |
|  | signal transduction in absence of ligand | BAK1 BCL2L1 GSK3B IL2 IL4 MCL1 | |
|  | extrinsic apoptotic signaling pathway in absence of ligand | BAK1 BCL2L1 GSK3B IL2 IL4 MCL1 | |
|  | apoptotic signaling pathway | BAK1 BCL2L1 GSK3B IL2 IL4 IL6R MCL1 TNF TP53 | |
|  | negative regulation of apoptotic signaling pathway | BAK1 BCL2L1 GSK3B HTT HSPB1 IL4 MCL1 TNF | |
|  | regulation of cellular response to stress | APP BAK1 BCL2L1 F2RL1 GSK3B HSPB1 MCL1 NPM1 TNF TP53 VEGFA | |
|  | regulation of apoptotic signaling pathway | BAK1 BCL2L1 GSK3B HTT HSPB1 IL4 MCL1 TNF TP53 | |
|  | negative regulation of extrinsic apoptotic signaling pathway | BCL2L1 GSK3B HTT IL4 MCL1 TNF | |
|  | intrinsic apoptotic signaling pathway in response to DNA damage | BAK1 BCL2L1 MCL1 TNF TP53 | |
|  | regulation of extrinsic apoptotic signaling pathway | BCL2L1 GSK3B HTT IL4 MCL1 TNF | |
|  | intrinsic apoptotic signaling pathway | BAK1 BCL2L1 MCL1 TNF TP53 | |
|  | regulation of neuron apoptotic process | BCL2L1 MCL1 SNCA TNF TP53 | |
|  | negative regulation of signal transduction in absence of ligand | BCL2L1 MCL1 TNF | |
|  | negative regulation of extrinsic apoptotic signaling pathway in absence of ligand | BCL2L1 MCL1 TNF | |
|  | regulation of extrinsic apoptotic signaling pathway in absence of ligand | BCL2L1 MCL1 TNF | |
|  | negative regulation of intracellular signal transduction | BCL2L1 MAPK14 F2RL1 GSK3B HSPB1 MCL1 | |
|  | positive regulation of neuron apoptotic process | MCL1 TNF TP53 | |
|  | regulation of intrinsic apoptotic signaling pathway | BCL2L1 HSPB1 MCL1 TP53 | |
|  | response to virus | BCL2L1 MAPK14 F2RL1 HSPB1 TNF | |
|  | regulation of oxidative stress-induced cell death | HSPB1 MCL1 TNF | |
|  | regulation of cellular response to oxidative stress | HSPB1 MCL1 TNF | |
|  | negative regulation of intrinsic apoptotic signaling pathway | BCL2L1 HSPB1 MCL1 | |
|  | regulation of response to oxidative stress | HSPB1 MCL1 TNF | |
|  | regulation of response to DNA damage stimulus | BCL2L1 MCL1 NPM1 TP53 | |
|  | positive regulation of apoptotic signaling pathway | MCL1 TNF TP53 | |
|  | regulation of cellular catabolic process | APP CDK2 MAPK14 GSK3B HTT HSPB1 IL4 MCL1 NPM1 PIK3CG SNCA TNF TP53 | |
|  | regulation of autophagy | GSK3B HTT HSPB1 IL4 MCL1 SNCA TP53 | |
|  | negative regulation of transferase activity | GSK3B HSPB1 NPM1 SNCA TP53 | |
|  | regulation of carbohydrate metabolic process | APP GSK3B SNCA TP53 | |
|  | cellular response to monoamine stimulus | APP GSK3B SNCA | |
|  | cellular response to catecholamine stimulus | APP GSK3B SNCA | |
|  | response to monoamine | APP GSK3B SNCA | |
|  | response to catecholamine | APP GSK3B SNCA | |
|  | regulation of cytoskeleton organization | EDN1 F2RL1 GSK3B NPM1 SNCA | |
|  | regulation of cellular carbohydrate metabolic process | GSK3B SNCA TP53 | |
|  | regulation of synaptic plasticity | APP GSK3B SNCA | |
|  | negative regulation of neuron death | BCL2L1 GSK3B SNCA | |
|  | regulation of microtubule-based process | GSK3B NPM1 SNCA | |
|  | energy derivation by oxidation of organic compounds | GSK3B SNCA TP53 | |
|  | cell surface receptor signaling pathway involved in cell-cell signaling | EDN1 GSK3B SNCA | |
|  | generation of precursor metabolites and energy | GSK3B SNCA TP53 | |
|  | cellular response to nitrogen compound | APP BCL2L1 CCNA2 CDK2 EDN1 FOS GSK3B NPM1 PIK3CG SNCA TNF TP53 | |
|  | cellular response to growth factor stimulus | APP CALCA CCNA2 MAPK14 EDN1 FOS HSPB1 CXCL8 TP53 VEGFA | |
|  | response to growth factor | APP CALCA CCNA2 MAPK14 EDN1 FOS HSPB1 CXCL8 TP53 VEGFA | |
|  | cellular response to organonitrogen compound | APP BCL2L1 CCNA2 EDN1 FOS GSK3B PIK3CG SNCA TNF TP53 | |
|  | response to hypoxia | CCNA2 DPP4 EDN1 FOS NOS2 TNF TP53 VEGFA | |
|  | response to decreased oxygen levels | CCNA2 DPP4 EDN1 FOS NOS2 TNF TP53 VEGFA | |
|  | response to oxygen levels | CCNA2 DPP4 EDN1 FOS NOS2 TNF TP53 VEGFA | |
|  | cellular response to organic cyclic compound | APP CCNA2 CDK1 EDN1 GSK3B NPM1 PIK3CG SNCA TNF | |
|  | response to peptide | APP CCNA2 MAPK14 EDN1 FOS GSK3B TNF TP53 | |
|  | cellular response to peptide | APP CCNA2 EDN1 FOS GSK3B TNF TP53 | |
|  | cellular response to lipid | CCNA2 MAPK14 EDN1 FOS GSK3B CXCL8 NOS2 TNF | |
|  | cellular response to hypoxia | CCNA2 EDN1 FOS TP53 VEGFA | |
|  | cellular response to decreased oxygen levels | CCNA2 EDN1 FOS TP53 VEGFA | |
|  | cellular response to oxygen levels | CCNA2 EDN1 FOS TP53 VEGFA | |
|  | DNA-templated transcription | CCNA2 CDK2 MAPK14 EDN1 FOS IL2 TP53 | |
|  | nucleic acid-templated transcription | CCNA2 CDK2 MAPK14 EDN1 FOS IL2 TP53 | |
|  | RNA biosynthetic process | CCNA2 CDK2 MAPK14 EDN1 FOS IL2 TP53 | |
|  | response to muscle stretch | MAPK14 EDN1 FOS | |
|  | response to hormone | CCNA2 MAPK14 EDN1 FOS GSK3B NOS2 TNF | |
|  | response to peptide hormone | CCNA2 MAPK14 EDN1 FOS GSK3B | |
|  | cellular response to peptide hormone stimulus | CCNA2 EDN1 FOS GSK3B | |
|  | transcription by RNA polymerase II | MAPK14 EDN1 FOS IL2 | |
|  | cellular response to transforming growth factor beta stimulus | EDN1 FOS TP53 | |
|  | response to transforming growth factor beta | EDN1 FOS TP53 | |
|  | cellular response to hormone stimulus | CCNA2 EDN1 FOS GSK3B | |
|  | regulation of generation of precursor metabolites and energy | APP CDK1 GSK3B IL4 NOS2 SNCA TNF TP53 | |
|  | regulation of nucleotide metabolic process | APP IL4 NOS2 SNCA TP53 | |
|  | regulation of cellular respiration | IL4 NOS2 SNCA TNF | |
|  | positive regulation of small molecule metabolic process | APP IL4 NOS2 SNCA TNF | |
|  | regulation of small molecule metabolic process | APP IL4 NOS2 SNCA TNF TP53 | |
|  | regulation of purine nucleotide metabolic process | APP IL4 NOS2 SNCA | |
|  | positive regulation of endocytosis | F2RL1 IL4 SNCA VEGFA | |
|  | regulation of cell killing | BCL2L1 F2RL1 IL4 NOS2 | |
|  | organ or tissue specific immune response | IL4 IL6R NOS2 | |
|  | regulation of purine nucleotide biosynthetic process | IL4 NOS2 SNCA | |
|  | regulation of nucleotide biosynthetic process | IL4 NOS2 SNCA | |
|  | positive regulation of nucleotide metabolic process | APP IL4 NOS2 | |
|  | positive regulation of purine nucleotide metabolic process | APP IL4 NOS2 | |
|  | regulation of endocytosis | F2RL1 IL4 SNCA VEGFA | |
|  | regulation of exocytosis | F2RL1 GSK3B IL4 SNCA | |
|  | positive regulation of exocytosis | F2RL1 IL4 SNCA | |
|  | positive regulation of secretion by cell | EDN1 F2RL1 IL4 SNCA | |
|  | positive regulation of secretion | EDN1 F2RL1 IL4 SNCA | |
|  | regulation of regulated secretory pathway | F2RL1 GSK3B IL4 | |
|  | organic hydroxy compound metabolic process | APP IL4 SNCA TP53 | |
|  | positive regulation of cellular component biogenesis | BAK1 CDK2 F2RL1 GSK3B HTT MMP1 SNCA TNF TP53 VEGFA | |
|  | positive regulation of cell death | BAK1 CD40LG FOS GSK3B HTT MCL1 NOS2 SNCA TNF TP53 | |
|  | positive regulation of neuron death | FOS GSK3B MCL1 SNCA TNF TP53 | |
|  | positive regulation of protein-containing complex assembly | BAK1 GSK3B MMP1 SNCA TNF TP53 VEGFA | |
|  | positive regulation of organelle organization | BAK1 CDK2 EDN1 F2RL1 GSK3B HTT SNCA TNF TP53 | |
|  | negative regulation of cellular component organization | BAK1 BCL2L1 DPP4 GSK3B NPM1 SNCA TNF TP53 VEGFA | |
|  | regulation of neuron death | BCL2L1 FOS GSK3B MCL1 SNCA TNF TP53 | |
|  | positive regulation of programmed cell death | BAK1 CD40LG HTT MCL1 NOS2 SNCA TNF TP53 | |
|  | regulation of protein-containing complex assembly | BAK1 GSK3B MMP1 SNCA TNF TP53 VEGFA | |
|  | regulation of proteolysis | APP BAK1 CDK2 GSK3B SNCA TNF TP53 VEGFA | |
|  | positive regulation of apoptotic process | BAK1 CD40LG HTT MCL1 SNCA TNF TP53 | |
|  | protein phosphorylation | APP BAK1 CDK1 CDK2 MAPK14 EDN1 GSK3B IL2 IL4 PIK3CG TOP1 | |
|  | peptidyl-serine phosphorylation | BAK1 CDK1 CDK2 MAPK14 GSK3B TOP1 | |
|  | peptidyl-serine modification | BAK1 CDK1 CDK2 MAPK14 GSK3B TOP1 | |
|  | regulation of developmental growth | APP CDK1 MAPK14 EDN1 GSK3B VEGFA | |
|  | positive regulation of protein localization | CDK1 MAPK14 F2RL1 GSK3B NPM1 TNF | |
|  | positive regulation of growth | CDK1 MAPK14 EDN1 IL2 VEGFA | |
|  | positive regulation of cardiac muscle tissue growth | CDK1 MAPK14 EDN1 | |
|  | positive regulation of heart growth | CDK1 MAPK14 EDN1 | |
|  | regulation of protein transport | CDK1 MAPK14 F2RL1 GSK3B NOS2 TNF | |
|  | regulation of protein localization to nucleus | CDK1 MAPK14 GSK3B NPM1 | |
|  | positive regulation of protein transport | CDK1 MAPK14 F2RL1 GSK3B TNF | |
|  | regulation of establishment of protein localization | CDK1 MAPK14 F2RL1 GSK3B NOS2 TNF | |
|  | positive regulation of establishment of protein localization | CDK1 MAPK14 F2RL1 GSK3B TNF | |
|  | positive regulation of organ growth | CDK1 MAPK14 EDN1 | |
|  | positive regulation of developmental growth | CDK1 MAPK14 EDN1 VEGFA | |
|  | positive regulation of nucleocytoplasmic transport | CDK1 MAPK14 GSK3B | |
|  | regulation of cardiac muscle tissue growth | CDK1 MAPK14 EDN1 | |
|  | regulation of heart growth | CDK1 MAPK14 EDN1 | |
|  | MAPK cascade | CDK1 MAPK14 EDN1 TNF | |
|  | positive regulation of protein localization to nucleus | CDK1 MAPK14 NPM1 | |
|  | regulation of organ growth | CDK1 MAPK14 EDN1 | |
|  | regulation of nucleocytoplasmic transport | CDK1 MAPK14 GSK3B | |
|  | cardiocyte differentiation | CDK1 EDN1 VEGFA | |
|  | muscle tissue development | CDK1 MAPK14 FOS VEGFA | |
|  | positive regulation of intracellular protein transport | CDK1 MAPK14 GSK3B | |
|  | positive regulation of intracellular transport | CDK1 MAPK14 GSK3B | |
|  | striated muscle cell differentiation | CDK1 MAPK14 VEGFA | |
|  | response to insulin | MAPK14 FOS GSK3B | |
|  | regulation of intracellular protein transport | CDK1 MAPK14 GSK3B | |
|  | muscle structure development | CDK1 MAPK14 FOS VEGFA | |
|  | muscle cell differentiation | CDK1 MAPK14 VEGFA | |
|  | regulation of Wnt signaling pathway | APP MAPK14 GSK3B | |
|  | regulation of intracellular transport | CDK1 MAPK14 GSK3B | |
|  | myeloid leukocyte activation | APP F2RL1 IL4 CXCL8 PIK3CG SNCA TNF | |
|  | microglial cell activation | APP IL4 SNCA TNF | |
|  | leukocyte activation involved in inflammatory response | APP IL4 SNCA TNF | |
|  | glial cell activation | APP IL4 SNCA TNF | |
|  | positive regulation of interleukin-6 production | APP F2RL1 IL6R NOS2 TNF | |
|  | neuroinflammatory response | APP IL4 SNCA TNF | |
|  | regulation of inflammatory response | APP MAPK14 IL2 IL4 PIK3CG SNCA TNF | |
|  | regulation of membrane potential | APP BAK1 BCL2L1 EDN1 GSK3B SNCA TNF | |
|  | positive regulation of DNA-binding transcription factor activity | APP CD40LG EDN1 NPM1 TNF VEGFA | |
|  | regulation of defense response | APP MAPK14 F2RL1 IL2 IL4 PIK3CG SNCA TNF | |
|  | regulation of peptidyl-serine phosphorylation | APP BAK1 SNCA TNF VEGFA | |
|  | macrophage activation | APP IL4 SNCA TNF | |
|  | positive regulation of inflammatory response | APP IL2 PIK3CG SNCA TNF | |
|  | positive regulation of interleukin-1 beta production | APP F2RL1 HSPB1 TNF | |
|  | positive regulation of defense response | APP F2RL1 IL2 PIK3CG SNCA TNF | |
|  | positive regulation of chemokine production | APP F2RL1 IL6R TNF | |
|  | regulation of interleukin-6 production | APP F2RL1 IL6R NOS2 TNF | |
|  | positive regulation of interleukin-1 production | APP F2RL1 HSPB1 TNF | |
|  | positive regulation of hydrolase activity | APP BAK1 F2RL1 GSK3B SNCA TNF VEGFA | |
|  | regulation of chemokine production | APP F2RL1 IL6R TNF | |
|  | positive regulation of cellular catabolic process | APP GSK3B HTT IL4 SNCA TNF | |
|  | positive regulation of peptidyl-serine phosphorylation | APP SNCA TNF VEGFA | |
|  | regulation of interleukin-1 beta production | APP F2RL1 HSPB1 TNF | |
|  | regulation of DNA-binding transcription factor activity | APP CD40LG EDN1 NPM1 TNF VEGFA | |
|  | regulation of interleukin-1 production | APP F2RL1 HSPB1 TNF | |
|  | positive regulation of stress-activated MAPK cascade | APP F2RL1 TNF VEGFA | |
|  | positive regulation of stress-activated protein kinase signaling cascade | APP F2RL1 TNF VEGFA | |
|  | positive regulation of catabolic process | APP GSK3B HTT IL4 SNCA TNF | |
|  | cellular response to amyloid-beta | APP GSK3B TNF | |
|  | regulation of vesicle-mediated transport | F2RL1 GSK3B IL4 SNCA TNF VEGFA | |
|  | positive regulation of NF-kappaB transcription factor activity | APP CD40LG NPM1 TNF | |
|  | response to amyloid-beta | APP GSK3B TNF | |
|  | negative regulation of proteolysis | APP SNCA TNF TP53 VEGFA | |
|  | positive regulation of proteolysis | APP BAK1 GSK3B SNCA TNF | |
|  | negative regulation of hydrolase activity | APP GSK3B SNCA TNF VEGFA | |
|  | positive regulation of NIK/NF-kappaB signaling | APP EDN1 TNF | |
|  | positive regulation of peptidase activity | APP BAK1 SNCA TNF | |
|  | regulation of stress-activated MAPK cascade | APP F2RL1 TNF VEGFA | |
|  | regulation of stress-activated protein kinase signaling cascade | APP F2RL1 TNF VEGFA | |
|  | regulation of cell junction assembly | APP SNCA TNF VEGFA | |
|  | regulation of cysteine-type endopeptidase activity involved in apoptotic process | BAK1 SNCA TNF VEGFA | |
|  | positive regulation of mononuclear cell migration | APP IL4 TNF | |
|  | regulation of synapse organization | APP MAPK14 SNCA TNF | |
|  | regulation of synapse structure or activity | APP MAPK14 SNCA TNF | |
|  | activation of cysteine-type endopeptidase activity involved in apoptotic process | BAK1 SNCA TNF | |
|  | regulation of endopeptidase activity | APP BAK1 SNCA TNF VEGFA | |
|  | negative regulation of cysteine-type endopeptidase activity involved in apoptotic process | SNCA TNF VEGFA | |
|  | modulation of chemical synaptic transmission | APP EDN1 GSK3B SNCA TNF | |
|  | regulation of trans-synaptic signaling | APP EDN1 GSK3B SNCA TNF | |
|  | regulation of cysteine-type endopeptidase activity | BAK1 SNCA TNF VEGFA | |
|  | regulation of peptidase activity | APP BAK1 SNCA TNF VEGFA | |
|  | negative regulation of cysteine-type endopeptidase activity | SNCA TNF VEGFA | |
|  | negative regulation of endopeptidase activity | APP SNCA TNF VEGFA | |
|  | positive regulation of JNK cascade | APP F2RL1 TNF | |
|  | negative regulation of peptidase activity | APP SNCA TNF VEGFA | |
|  | regulation of NIK/NF-kappaB signaling | APP EDN1 TNF | |
|  | regulation of mononuclear cell migration | APP IL4 TNF | |
|  | positive regulation of cysteine-type endopeptidase activity involved in apoptotic process | BAK1 SNCA TNF | |
|  | regulation of JNK cascade | APP F2RL1 TNF | |
|  | positive regulation of cysteine-type endopeptidase activity | BAK1 SNCA TNF | |
|  | regulation of nervous system process | APP EDN1 TNF | |
|  | positive regulation of synaptic transmission | APP SNCA TNF | |
|  | positive regulation of endopeptidase activity | BAK1 SNCA TNF | |
|  | regulation of signaling receptor activity | APP EDN1 TNF | |
|  | positive regulation of ERK1 and ERK2 cascade | APP F2RL1 TNF | |
|  | gliogenesis | APP TNF TP53 | |
|  | regulation of I-kappaB kinase/NF-kappaB signaling | F2RL1 HSPB1 TNF | |
|  | extracellular matrix organization | APP MMP1 TNF | |
|  | extracellular structure organization | APP MMP1 TNF | |
|  | external encapsulating structure organization | APP MMP1 TNF | |
|  | regulation of ERK1 and ERK2 cascade | APP F2RL1 TNF | |
|  | response to xenobiotic stimulus | BAK1 CDK1 EDN1 FOS NOS2 SNCA TNF TOP1 TP53 | |
|  | circadian rhythm | GSK3B NOS2 TNF TOP1 TP53 | |
|  | rhythmic process | CDK1 GSK3B NOS2 TNF TOP1 TP53 | |
|  | regulation of circadian rhythm | CDK1 GSK3B TP53 | |
|  | cellular component disassembly | CDK1 GSK3B MMP1 TP53 | |
|  | regulation of growth | APP BCL2L1 CDK1 MAPK14 EDN1 GSK3B IL2 NPM1 TP53 VEGFA | |
|  | regulation of epithelial cell migration | EDN1 HSPB1 IL4 PIK3CG TNF VEGFA | |
|  | regulation of anatomical structure size | CALCA EDN1 F2RL1 GSK3B NPM1 TNF VEGFA | |
|  | regulation of endothelial cell migration | EDN1 HSPB1 PIK3CG TNF VEGFA | |
|  | regulation of cell growth | EDN1 GSK3B IL2 NPM1 TP53 VEGFA | |
|  | positive regulation of MAP kinase activity | EDN1 PIK3CG TNF VEGFA | |
|  | positive regulation of endothelial cell migration | EDN1 HSPB1 PIK3CG VEGFA | |
|  | positive regulation of MAPK cascade | APP EDN1 F2RL1 PIK3CG TNF VEGFA | |
|  | response to acid chemical | BCL2L1 EDN1 TNF VEGFA | |
|  | positive regulation of epithelial cell migration | EDN1 HSPB1 PIK3CG VEGFA | |
|  | regulation of cellular component size | EDN1 F2RL1 GSK3B NPM1 VEGFA | |
|  | regulation of cell size | EDN1 GSK3B NPM1 VEGFA | |
|  | regulation of MAP kinase activity | EDN1 PIK3CG TNF VEGFA | |
|  | regulation of MAPK cascade | APP EDN1 F2RL1 PIK3CG TNF VEGFA | |
|  | cellular response to acid chemical | BCL2L1 TNF VEGFA | |
|  | response to amino acid | BCL2L1 EDN1 TNF | |
|  | regulation of animal organ morphogenesis | EDN1 TNF VEGFA | |
|  | in utero embryonic development | BCL2L1 EDN1 TP53 VEGFA | |
|  | positive regulation of cell growth | EDN1 IL2 VEGFA | |
|  | cell maturation | APP EDN1 VEGFA | |
|  | ameboidal-type cell migration | DPP4 EDN1 VEGFA | |
|  | anatomical structure maturation | APP EDN1 VEGFA | |
|  | cell part morphogenesis | APP BCL2L1 EDN1 VEGFA | |
|  | axon guidance | APP EDN1 VEGFA | |
|  | neuron projection guidance | APP EDN1 VEGFA | |
|  | heart morphogenesis | EDN1 TP53 VEGFA | |
|  | developmental maturation | APP EDN1 VEGFA | |
|  | heart development | CDK1 EDN1 TP53 VEGFA | |
|  | sensory organ development | BAK1 CCNA2 EDN1 VEGFA | |
|  | sensory organ morphogenesis | BAK1 EDN1 VEGFA | |
|  | cellular component morphogenesis | APP BCL2L1 EDN1 VEGFA | |
|  | chordate embryonic development | BCL2L1 EDN1 TP53 VEGFA | |
|  | neuron projection development | APP EDN1 GSK3B VEGFA | |
|  | embryo development ending in birth or egg hatching | BCL2L1 EDN1 TP53 VEGFA | |
|  | cell morphogenesis | APP MAPK14 EDN1 VEGFA | |
|  | axonogenesis | APP EDN1 VEGFA | |
|  | axon development | APP EDN1 VEGFA | |
|  | gland development | EDN1 TNF VEGFA | |
|  | cell morphogenesis involved in neuron differentiation | APP EDN1 VEGFA | |
|  | response to radiation | APP BAK1 BCL2L1 MAPK14 FOS MMP1 NPM1 TNF TP53 | |
|  | cellular response to radiation | BAK1 BCL2L1 MAPK14 MMP1 NPM1 TP53 | |
|  | mitochondrial membrane organization | BAK1 BCL2L1 GSK3B SNCA TP53 | |
|  | regulation of mitochondrion organization | BAK1 BCL2L1 GSK3B HTT TP53 | |
|  | regulation of mitochondrial membrane permeability | BAK1 BCL2L1 GSK3B TP53 | |
|  | response to light stimulus | APP BAK1 FOS MMP1 NPM1 TP53 | |
|  | cellular response to abiotic stimulus | BAK1 BCL2L1 MAPK14 MMP1 NPM1 TP53 | |
|  | cellular response to environmental stimulus | BAK1 BCL2L1 MAPK14 MMP1 NPM1 TP53 | |
|  | release of cytochrome c from mitochondria | BAK1 BCL2L1 TP53 | |
|  | positive regulation of mitochondrion organization | BAK1 GSK3B HTT TP53 | |
|  | regulation of membrane permeability | BAK1 BCL2L1 GSK3B TP53 | |
|  | cellular response to UV | BAK1 MMP1 NPM1 TP53 | |
|  | neuron apoptotic process | APP BCL2L1 SNCA TP53 | |
|  | neuron death | APP BCL2L1 SNCA TP53 | |
|  | mitochondrion organization | BAK1 BCL2L1 EDN1 GSK3B SNCA TP53 | |
|  | positive regulation of mitochondrial membrane permeability | BAK1 GSK3B TP53 | |
|  | cellular response to light stimulus | BAK1 MMP1 NPM1 TP53 | |
|  | positive regulation of membrane permeability | BAK1 GSK3B TP53 | |
|  | B cell differentiation | BAK1 CD40LG IL4 TP53 | |
|  | leukocyte apoptotic process | BAK1 BCL2L1 TP53 | |
|  | response to ionizing radiation | BAK1 BCL2L1 MAPK14 TP53 | |
|  | regulation of release of cytochrome c from mitochondria | BAK1 BCL2L1 TP53 | |
|  | response to UV | BAK1 MMP1 NPM1 TP53 | |
|  | mitochondrial transport | BAK1 BCL2L1 GSK3B TP53 | |
|  | negative regulation of mitochondrion organization | BAK1 BCL2L1 TP53 | |
|  | negative regulation of organelle organization | BAK1 BCL2L1 NPM1 SNCA TP53 | |
|  | response to gamma radiation | BAK1 BCL2L1 TP53 | |
|  | apoptotic mitochondrial changes | BAK1 BCL2L1 TP53 | |
|  | B cell activation | BAK1 CD40LG IL4 TP53 | |
|  | cellular response to ionizing radiation | BCL2L1 MAPK14 TP53 | |
|  | response to endoplasmic reticulum stress | BAK1 GSK3B CXCL8 TP53 | |
|  | regulation of leukocyte apoptotic process | BCL2L1 IL2 TP53 | |
|  | female sex differentiation | BAK1 BCL2L1 VEGFA | |
|  | epithelial cell proliferation | BAK1 BCL2L1 TNF | |
|  | sex differentiation | BAK1 BCL2L1 VEGFA | |
|  | reproductive structure development | BAK1 BCL2L1 VEGFA | |
|  | reproductive system development | BAK1 BCL2L1 VEGFA | |
|  | cellular response to external stimulus | BAK1 FOS TP53 | |
|  | brain development | APP BAK1 GSK3B TP53 | |
|  | cellular response to tumor necrosis factor | CALCA MAPK14 EDN1 FOS CXCL8 TNF TP53 | |
|  | response to tumor necrosis factor | CALCA MAPK14 EDN1 FOS CXCL8 TNF TP53 | |
|  | cellular response to cytokine stimulus | CALCA MAPK14 EDN1 FOS GSK3B IL6R CXCL8 NOS2 TNF TP53 | |
|  | response to lipopolysaccharide | MAPK14 EDN1 FOS CXCL8 NOS2 SNCA TNF | |
|  | response to molecule of bacterial origin | MAPK14 EDN1 FOS CXCL8 NOS2 SNCA TNF | |
|  | cellular response to biotic stimulus | MAPK14 GSK3B CXCL8 NOS2 TNF TP53 | |
|  | response to bacterium | MAPK14 EDN1 FOS IL6R CXCL8 NOS2 SNCA TNF | |
|  | positive regulation of miRNA transcription | FOS TNF TP53 | |
|  | osteoclast differentiation | MAPK14 FOS TNF | |
|  | positive regulation of miRNA metabolic process | FOS TNF TP53 | |
|  | regulation of miRNA transcription | FOS TNF TP53 | |
|  | cellular response to lipopolysaccharide | MAPK14 CXCL8 NOS2 TNF | |
|  | regulation of miRNA metabolic process | FOS TNF TP53 | |
|  | regulation of cytokine production involved in inflammatory response | MAPK14 NOS2 TNF | |
|  | cellular response to molecule of bacterial origin | MAPK14 CXCL8 NOS2 TNF | |
|  | positive regulation of myeloid cell differentiation | MAPK14 FOS TNF | |
|  | glucose metabolic process | MAPK14 TNF TP53 | |
|  | hexose metabolic process | MAPK14 TNF TP53 | |
|  | monosaccharide metabolic process | MAPK14 TNF TP53 | |
|  | transmembrane receptor protein serine/threonine kinase signaling pathway | MAPK14 FOS TP53 | |
|  | response to nutrient levels | MAPK14 FOS TNF TP53 | |
|  | carbohydrate metabolic process | MAPK14 GSK3B TNF TP53 | |
|  | response to extracellular stimulus | MAPK14 FOS TNF TP53 | |
|  | regulation of cell cycle process | APP BCL2L1 CCND3 CDK1 CDK2 MAPK14 EDN1 NPM1 TNF TP53 | |
|  | cellular response to DNA damage stimulus | BAK1 BCL2L1 CDK1 CDK2 MAPK14 MCL1 NPM1 TNF TP53 | |
|  | regulation of cell cycle G2/M phase transition | APP CDK1 CDK2 NPM1 TP53 | |
|  | regulation of cell cycle phase transition | APP CCND3 CDK1 CDK2 MAPK14 NPM1 TP53 | |
|  | G2/M transition of mitotic cell cycle | APP CCNA2 CDK1 CDK2 | |
|  | cell cycle G2/M phase transition | APP CCNA2 CDK1 CDK2 | |
|  | cellular senescence | CDK2 MAPK14 NPM1 TP53 | |
|  | regulation of mitotic cell cycle | APP CCND3 CDK1 CDK2 EDN1 TNF TP53 | |
|  | mitotic cell cycle phase transition | APP CCNA2 CCND3 CDK1 CDK2 | |
|  | mitotic cell cycle process | APP CCNA2 CCND3 CDK1 CDK2 HTT TP53 | |
|  | cell cycle phase transition | APP CCNA2 CCND3 CDK1 CDK2 | |
|  | negative regulation of cell cycle | CDK1 CDK2 MAPK14 NPM1 TNF TP53 | |
|  | mitotic cell cycle | APP CCNA2 CCND3 CDK1 CDK2 HTT TP53 | |
|  | DNA damage checkpoint signaling | CDK1 CDK2 MAPK14 TP53 | |
|  | DNA integrity checkpoint signaling | CDK1 CDK2 MAPK14 TP53 | |
|  | signal transduction in response to DNA damage | CDK1 CDK2 MAPK14 TP53 | |
|  | negative regulation of cell cycle process | CDK1 CDK2 MAPK14 NPM1 TP53 | |
|  | regulation of DNA replication | CCNA2 CDK1 CDK2 TP53 | |
|  | DNA metabolic process | CD40LG CDK1 CDK2 FOS NPM1 TOP1 TP53 | |
|  | cell cycle checkpoint signaling | CDK1 CDK2 MAPK14 TP53 | |
|  | regulation of mitotic cell cycle phase transition | APP CCND3 CDK1 CDK2 TP53 | |
|  | mitotic DNA damage checkpoint signaling | CDK1 CDK2 TP53 | |
|  | mitotic DNA integrity checkpoint signaling | CDK1 CDK2 TP53 | |
|  | negative regulation of mitotic cell cycle | CDK1 CDK2 TNF TP53 | |
|  | cell cycle G1/S phase transition | CCNA2 CCND3 CDK2 | |
|  | centrosome cycle | CDK1 CDK2 NPM1 | |
|  | negative regulation of cell cycle phase transition | CDK1 CDK2 MAPK14 TP53 | |
|  | regulation of G2/M transition of mitotic cell cycle | APP CDK1 CDK2 | |
|  | cell division | CCNA2 CCND3 CDK1 CDK2 TOP1 | |
|  | microtubule organizing center organization | CDK1 CDK2 NPM1 | |
|  | mitotic cell cycle checkpoint signaling | CDK1 CDK2 TP53 | |
|  | negative regulation of mitotic cell cycle phase transition | CDK1 CDK2 TP53 | |
|  | regulation of G1/S transition of mitotic cell cycle | CCND3 CDK2 TP53 | |
|  | Ras protein signal transduction | CCNA2 CDK2 TP53 | |
|  | DNA replication | CDK1 CDK2 TOP1 | |
|  | regulation of cell cycle G1/S phase transition | CCND3 CDK2 TP53 | |
|  | DNA repair | CDK1 CDK2 NPM1 TP53 | |
|  | microtubule cytoskeleton organization | CDK1 CDK2 HTT NPM1 | |
|  | small GTPase mediated signal transduction | CCNA2 CDK2 TP53 | |
|  | regulation of peptidyl-tyrosine phosphorylation | APP IL2 IL4 IL6R TNF TP53 VEGFA | |
|  | positive regulation of peptidyl-tyrosine phosphorylation | IL2 IL4 IL6R TNF TP53 VEGFA | |
|  | negative regulation of cell differentiation | APP GSK3B IL2 IL4 TNF TP53 VEGFA | |
|  | negative regulation of cell development | GSK3B TNF TP53 VEGFA | |
|  | embryonic organ development | EDN1 CXCL8 TNF TP53 VEGFA | |
|  | cytokine-mediated signaling pathway | IL6R CXCL8 TNF TP53 | |
|  | regulation of cell development | GSK3B TNF TP53 VEGFA | |
|  | regulation of neurogenesis | TNF TP53 VEGFA | |
|  | negative regulation of phosphate metabolic process | BAK1 GSK3B HSPB1 IL2 NPM1 SNCA TNF TP53 | |
|  | negative regulation of phosphorus metabolic process | BAK1 GSK3B HSPB1 IL2 NPM1 SNCA TNF TP53 | |
|  | negative regulation of protein modification process | BAK1 GSK3B HSPB1 IL2 NPM1 SNCA TNF | |
|  | negative regulation of catalytic activity | APP GSK3B HSPB1 NPM1 SNCA TNF TP53 VEGFA | |
|  | negative regulation of cell population proliferation | APP BAK1 IL2 CXCL8 NPM1 TNF TP53 | |
|  | regulation of cellular amide metabolic process | APP HSPB1 NPM1 SNCA TNF TP53 | |
|  | negative regulation of protein phosphorylation | BAK1 HSPB1 IL2 NPM1 SNCA | |
|  | negative regulation of phosphorylation | BAK1 HSPB1 IL2 NPM1 SNCA | |
|  | regulation of translational initiation | HSPB1 NPM1 TNF | |
|  | regulation of cellular macromolecule biosynthetic process | APP GSK3B HSPB1 NPM1 TNF | |
|  | negative regulation of protein kinase activity | HSPB1 NPM1 SNCA | |
|  | regulation of translation | APP HSPB1 NPM1 TNF | |
|  | negative regulation of kinase activity | HSPB1 NPM1 SNCA | |
|  | negative regulation of catabolic process | MAPK14 MCL1 NOS2 PIK3CG SNCA TNF TP53 | |
|  | regulation of reactive oxygen species metabolic process | MAPK14 F2RL1 SNCA TNF TP53 | |
|  | positive regulation of reactive oxygen species metabolic process | MAPK14 F2RL1 SNCA TP53 | |
|  | negative regulation of cellular catabolic process | MAPK14 MCL1 PIK3CG SNCA TP53 | |
|  | negative regulation of autophagy | MCL1 SNCA TP53 | |
|  | regulation of histone modification | SNCA TP53 VEGFA | |
|  | stem cell differentiation | MAPK14 EDN1 TP53 | |
|  | monocarboxylic acid metabolic process | MAPK14 EDN1 SNCA TP53 | |
|  | fatty acid metabolic process | MAPK14 EDN1 SNCA | |
|  | response to inorganic substance | APP BAK1 CCNA2 CDK1 CDK2 EDN1 FOS SNCA | |
|  | response to oxidative stress | APP BAK1 CDK1 EDN1 FOS SNCA TP53 | |
|  | cellular response to inorganic substance | APP CCNA2 CDK2 EDN1 FOS SNCA | |
|  | cellular response to oxidative stress | CDK1 EDN1 FOS SNCA TP53 | |
|  | cellular response to chemical stress | CDK1 EDN1 FOS SNCA TP53 | |
|  | response to copper ion | APP CDK1 SNCA | |
|  | response to reactive oxygen species | BAK1 CDK1 EDN1 FOS | |
|  | response to metal ion | APP CDK1 EDN1 FOS SNCA | |
|  | cellular response to metal ion | APP EDN1 FOS SNCA | |
|  | response to hydrogen peroxide | BAK1 CDK1 EDN1 | |
|  | cellular response to reactive oxygen species | CDK1 EDN1 FOS | |
|  | organelle fusion | BAK1 CDK1 SNCA | |
|  | positive regulation of cytosolic calcium ion concentration | BAK1 CALCA EDN1 F2RL1 IL2 PIK3CG | |
|  | response to mechanical stimulus | BAK1 MAPK14 EDN1 FOS HTT TNF | |
|  | response to interferon-gamma | EDN1 NOS2 SNCA TNF TP53 | |
|  | response to activity | CDK1 EDN1 FOS TNF | |
|  | regulation of secretion by cell | EDN1 F2RL1 GSK3B IL4 NOS2 SNCA TNF | |
|  | regulation of blood pressure | CALCA EDN1 F2RL1 NOS2 TNF | |
|  | regulation of system process | APP CALCA EDN1 F2RL1 IL2 PIK3CG TNF | |
|  | regulation of heart contraction | CALCA EDN1 IL2 PIK3CG TNF | |
|  | regulation of secretion | EDN1 F2RL1 GSK3B IL4 NOS2 SNCA TNF | |
|  | regulation of systemic arterial blood pressure | CALCA EDN1 F2RL1 TNF | |
|  | blood circulation | CALCA EDN1 F2RL1 NOS2 TNF VEGFA | |
|  | cellular response to interferon-gamma | EDN1 NOS2 TNF TP53 | |
|  | positive regulation of cytosolic calcium ion concentration involved in phospholipase C-activating G protein-coupled signaling pathway | CALCA EDN1 F2RL1 | |
|  | regulation of blood circulation | CALCA EDN1 IL2 PIK3CG TNF | |
|  | vascular process in circulatory system | CALCA EDN1 F2RL1 TNF VEGFA | |
|  | circulatory system process | CALCA EDN1 F2RL1 NOS2 TNF VEGFA | |
|  | regulation of tube diameter | CALCA EDN1 F2RL1 TNF | |
|  | blood vessel diameter maintenance | CALCA EDN1 F2RL1 TNF | |
|  | regulation of tube size | CALCA EDN1 F2RL1 TNF | |
|  | negative regulation of blood pressure | CALCA NOS2 TNF | |
|  | vasodilation | CALCA F2RL1 TNF | |
|  | regulation of protein catabolic process | CDK2 GSK3B NOS2 SNCA TNF | |
|  | positive regulation of oxidoreductase activity | EDN1 SNCA TNF | |
|  | positive regulation of interleukin-8 production | F2RL1 NOS2 TNF | |
|  | sensory perception of pain | CALCA EDN1 TNF | |
|  | regulation of organic acid transport | EDN1 SNCA TNF | |
|  | regulation of glucose transmembrane transport | MAPK14 EDN1 TNF | |
|  | second-messenger-mediated signaling | EDN1 CXCL8 NOS2 TNF | |
|  | regulation of anion transport | EDN1 SNCA TNF | |
|  | cell fate commitment | EDN1 IL6R MCL1 TP53 | |
|  | positive regulation of smooth muscle cell proliferation | EDN1 IL6R TNF | |
|  | phospholipase C-activating G protein-coupled receptor signaling pathway | CALCA EDN1 F2RL1 | |
|  | regulation of interleukin-8 production | F2RL1 NOS2 TNF | |
|  | reactive oxygen species metabolic process | EDN1 NOS2 TP53 | |
|  | secretion | EDN1 NOS2 PIK3CG SNCA VEGFA | |
|  | regulation of oxidoreductase activity | EDN1 SNCA TNF | |
|  | endocrine system development | BAK1 EDN1 IL6R | |
|  | regulation of wound healing | EDN1 F2RL1 TNF | |
|  | response to glucocorticoid | EDN1 FOS TNF | |
|  | calcium-mediated signaling | EDN1 CXCL8 TNF | |
|  | response to corticosteroid | EDN1 FOS TNF | |
|  | regulation of response to wounding | EDN1 F2RL1 TNF | |
|  | regulation of muscle contraction | CALCA EDN1 PIK3CG | |
|  | secretion by cell | EDN1 NOS2 PIK3CG SNCA | |
|  | regulation of smooth muscle cell proliferation | EDN1 IL6R TNF | |
|  | negative regulation of secretion | EDN1 SNCA TNF | |
|  | signal release | EDN1 NOS2 SNCA | |
|  | cellular response to xenobiotic stimulus | EDN1 NOS2 TP53 | |
|  | export from cell | EDN1 NOS2 PIK3CG SNCA | |
|  | muscle contraction | CALCA EDN1 TNF | |
|  | regulation of hormone secretion | EDN1 NOS2 TNF | |
|  | regulation of hormone levels | DPP4 EDN1 NOS2 TNF | |
|  | regulation of muscle system process | CALCA EDN1 PIK3CG | |
|  | regulation of protein secretion | F2RL1 NOS2 TNF | |
|  | response to steroid hormone | EDN1 FOS TNF | |
|  | muscle system process | CALCA EDN1 TNF | |
|  | defense response to bacterium | IL6R NOS2 TNF | |
|  | regulation of DNA metabolic process | CCNA2 CDK1 CDK2 IL2 IL4 NPM1 TNF TP53 | |
|  | positive regulation of leukocyte mediated immunity | F2RL1 IL2 IL4 NOS2 TNF | |
|  | positive regulation of cell adhesion | CD40LG DPP4 GSK3B IL2 IL4 TNF VEGFA | |
|  | regulation of cell-cell adhesion | CD40LG MAPK14 DPP4 IL2 IL4 TNF VEGFA | |
|  | positive regulation of tyrosine phosphorylation of STAT protein | IL2 IL4 IL6R TNF | |
|  | regulation of immunoglobulin production | CD40LG IL2 IL4 TNF | |
|  | regulation of production of molecular mediator of immune response | CD40LG F2RL1 IL2 IL4 TNF | |
|  | regulation of tyrosine phosphorylation of STAT protein | IL2 IL4 IL6R TNF | |
|  | regulation of immune effector process | CD40LG F2RL1 IL2 IL4 NOS2 TNF | |
|  | regulation of leukocyte activation | CD40LG DPP4 F2RL1 IL2 IL4 SNCA TNF | |
|  | regulation of leukocyte mediated immunity | F2RL1 IL2 IL4 NOS2 TNF | |
|  | negative regulation of response to external stimulus | MAPK14 DPP4 EDN1 IL2 IL4 TNF | |
|  | positive regulation of leukocyte activation | CD40LG DPP4 F2RL1 IL2 IL4 TNF | |
|  | positive regulation of immune effector process | F2RL1 IL2 IL4 NOS2 TNF | |
|  | positive regulation of cell activation | CD40LG DPP4 F2RL1 IL2 IL4 TNF | |
|  | regulation of cell activation | CD40LG DPP4 F2RL1 IL2 IL4 SNCA TNF | |
|  | positive regulation of leukocyte cell-cell adhesion | CD40LG DPP4 IL2 IL4 TNF | |
|  | positive regulation of interleukin-10 production | CD40LG F2RL1 IL4 | |
|  | positive regulation of B cell mediated immunity | IL2 IL4 TNF | |
|  | positive regulation of immunoglobulin mediated immune response | IL2 IL4 TNF | |
|  | lymphocyte costimulation | CD40LG DPP4 IL4 | |
|  | positive regulation of DNA metabolic process | CDK1 CDK2 IL2 IL4 TNF | |
|  | positive regulation of mononuclear cell proliferation | BCL2L1 CD40LG IL2 IL4 | |
|  | positive regulation of cell-cell adhesion | CD40LG DPP4 IL2 IL4 TNF | |
|  | positive regulation of leukocyte proliferation | BCL2L1 CD40LG IL2 IL4 | |
|  | regulation of endothelial cell apoptotic process | CD40LG IL4 TNF | |
|  | positive regulation of leukocyte differentiation | FOS IL2 IL4 TNF | |
|  | positive regulation of hemopoiesis | FOS IL2 IL4 TNF | |
|  | regulation of leukocyte cell-cell adhesion | CD40LG DPP4 IL2 IL4 TNF | |
|  | regulation of B cell mediated immunity | IL2 IL4 TNF | |
|  | regulation of immunoglobulin mediated immune response | IL2 IL4 TNF | |
|  | regulation of interleukin-10 production | CD40LG F2RL1 IL4 | |
|  | regulation of osteoclast differentiation | FOS IL4 TNF | |
|  | regulation of hemopoiesis | MAPK14 FOS IL2 IL4 TNF | |
|  | regulation of myeloid cell differentiation | MAPK14 FOS IL4 TNF | |
|  | positive regulation of interferon-gamma production | F2RL1 IL2 TNF | |
|  | negative regulation of immune system process | MAPK14 DPP4 IL2 IL4 TNF | |
|  | regulation of mononuclear cell proliferation | BCL2L1 CD40LG IL2 IL4 | |
|  | positive regulation of T cell activation | CD40LG DPP4 IL2 IL4 | |
|  | regulation of leukocyte proliferation | BCL2L1 CD40LG IL2 IL4 | |
|  | positive regulation of T cell proliferation | CD40LG IL2 IL4 | |
|  | regulation of epithelial cell apoptotic process | CD40LG IL4 TNF | |
|  | positive regulation of adaptive immune response based on somatic recombination of immune receptors built from immunoglobulin superfamily domains | IL2 IL4 TNF | |
|  | regulation of cytokine production involved in immune response | F2RL1 IL4 TNF | |
|  | positive regulation of lymphocyte mediated immunity | IL2 IL4 TNF | |
|  | regulation of interferon-gamma production | F2RL1 IL2 TNF | |
|  | positive regulation of adaptive immune response | IL2 IL4 TNF | |
|  | negative regulation of immune effector process | IL2 IL4 TNF | |
|  | regulation of myeloid leukocyte differentiation | FOS IL4 TNF | |
|  | regulation of leukocyte differentiation | FOS IL2 IL4 TNF | |
|  | positive regulation of production of molecular mediator of immune response | F2RL1 IL2 IL4 | |
|  | activation of protein kinase activity | CALCA IL4 VEGFA | |
|  | positive regulation of lymphocyte proliferation | CD40LG IL2 IL4 | |
|  | regulation of T cell activation | CD40LG DPP4 IL2 IL4 | |
|  | positive regulation of lymphocyte activation | CD40LG DPP4 IL2 IL4 | |
|  | regulation of lymphocyte mediated immunity | IL2 IL4 TNF | |
|  | regulation of adaptive immune response based on somatic recombination of immune receptors built from immunoglobulin superfamily domains | IL2 IL4 TNF | |
|  | regulation of T cell proliferation | CD40LG IL2 IL4 | |
|  | negative regulation of inflammatory response | MAPK14 IL2 IL4 | |
|  | negative regulation of immune response | MAPK14 IL2 IL4 | |
|  | regulation of adaptive immune response | IL2 IL4 TNF | |
|  | negative regulation of cell-cell adhesion | IL2 IL4 VEGFA | |
|  | regulation of lymphocyte proliferation | CD40LG IL2 IL4 | |
|  | regulation of lymphocyte activation | CD40LG DPP4 IL2 IL4 | |
|  | negative regulation of defense response | MAPK14 IL2 IL4 | |
|  | positive regulation of immune response | F2RL1 IL2 IL4 TNF | |
|  | negative regulation of cell adhesion | IL2 IL4 VEGFA | |
|  | negative regulation of cell migration | DPP4 IL4 TNF | |
|  | negative regulation of cytokine production | F2RL1 IL4 TNF | |
|  | negative regulation of cell motility | DPP4 IL4 TNF | |
|  | negative regulation of locomotion | DPP4 IL4 TNF | |
|  | regulation of body fluid levels | CD40LG MAPK14 EDN1 F2RL1 HSPB1 PIK3CG VEGFA | |
|  | response to wounding | CD40LG CDK1 MAPK14 F2RL1 HSPB1 PIK3CG VEGFA | |
|  | blood coagulation | CD40LG MAPK14 F2RL1 HSPB1 PIK3CG | |
|  | coagulation | CD40LG MAPK14 F2RL1 HSPB1 PIK3CG | |
|  | hemostasis | CD40LG MAPK14 F2RL1 HSPB1 PIK3CG | |
|  | wound healing | CD40LG MAPK14 F2RL1 HSPB1 PIK3CG VEGFA | |
|  | platelet activation | CD40LG MAPK14 HSPB1 PIK3CG | |
|  | cell-cell adhesion | CD40LG HSPB1 PIK3CG TNF | |
|  | regulation of transmembrane transport | APP BAK1 MAPK14 EDN1 HTT PIK3CG SNCA TNF | |
|  | regulation of ion transmembrane transport | APP BAK1 EDN1 HTT PIK3CG SNCA TNF | |
|  | regulation of cation transmembrane transport | APP BAK1 EDN1 HTT PIK3CG SNCA | |
|  | positive regulation of transmembrane transport | BAK1 MAPK14 EDN1 HTT SNCA | |
|  | regulation of ion transport | APP BAK1 EDN1 HTT PIK3CG SNCA TNF | |
|  | positive regulation of cation transmembrane transport | BAK1 EDN1 HTT SNCA | |
|  | positive regulation of ion transmembrane transport | BAK1 EDN1 HTT SNCA | |
|  | regulation of calcium ion transmembrane transport | BAK1 HTT PIK3CG SNCA | |
|  | positive regulation of calcium ion transmembrane transport | BAK1 HTT SNCA | |
|  | regulation of calcium ion transport | BAK1 HTT PIK3CG SNCA | |
|  | positive regulation of ion transport | BAK1 EDN1 HTT SNCA | |
|  | positive regulation of calcium ion transport | BAK1 HTT SNCA | |
|  | regulation of protein stability | HTT NPM1 SNCA TP53 | |
|  | establishment of organelle localization | HTT NPM1 PIK3CG SNCA | |
|  | vesicle localization | HTT PIK3CG SNCA | |
|  | regulation of metal ion transport | BAK1 HTT PIK3CG SNCA | |
|  | organelle localization | HTT NPM1 PIK3CG SNCA | |
|  | response to ethanol | BAK1 CDK1 FOS IL2 TNF | |
|  | response to alcohol | BAK1 BCL2L1 CDK1 FOS IL2 TNF | |
|  | animal organ regeneration | BAK1 CCNA2 CDK1 TNF | |
|  | regeneration | BAK1 CCNA2 CDK1 TNF | |
|  | response to toxic substance | BAK1 CDK1 FOS TNF | |
|  | positive regulation of cell cycle process | APP CCND3 CDK1 EDN1 NPM1 TNF | |
|  | positive regulation of cell cycle | APP CCND3 CDK1 EDN1 NPM1 TNF | |
|  | positive regulation of cell cycle G2/M phase transition | APP CDK1 NPM1 | |
|  | positive regulation of cell cycle phase transition | APP CCND3 CDK1 NPM1 | |
|  | positive regulation of mitotic cell cycle phase transition | APP CCND3 CDK1 | |
|  | positive regulation of mitotic cell cycle | APP CCND3 CDK1 | |
|  | regulation of granulocyte chemotaxis | DPP4 EDN1 IL4 CXCL8 | |
|  | positive regulation of granulocyte chemotaxis | EDN1 IL4 CXCL8 | |
|  | regulation of neutrophil chemotaxis | DPP4 EDN1 CXCL8 | |
|  | response to interleukin-1 | APP EDN1 CXCL8 SNCA | |
|  | endocytosis | APP BCL2L1 DPP4 CXCL8 PIK3CG SNCA | |
|  | regulation of neutrophil migration | DPP4 EDN1 CXCL8 | |
|  | regulation of G protein-coupled receptor signaling pathway | EDN1 CXCL8 SNCA | |
|  | receptor-mediated endocytosis | DPP4 CXCL8 SNCA | |
|  | locomotory behavior | APP DPP4 SNCA | |
|  | behavior | APP CALCA DPP4 FOS HTT SNCA TP53 | |
|  | response to cAMP | APP FOS PIK3CG | |
|  | feeding behavior | APP CALCA FOS | |
|  | cognition | APP FOS HTT TNF | |
|  | response to organophosphorus | APP FOS PIK3CG | |
|  | response to purine-containing compound | APP FOS PIK3CG | |
|  | learning | APP FOS HTT | |
|  | transport along microtubule | APP HTT HSPB1 | |
|  | regulation of tumor necrosis factor production | APP HSPB1 IL4 | |
|  | regulation of tumor necrosis factor superfamily cytokine production | APP HSPB1 IL4 | |
|  | cytoskeleton-dependent intracellular transport | APP HTT HSPB1 | |
|  | microtubule-based transport | APP HTT HSPB1 | |
|  | learning or memory | APP FOS HTT | |
|  | anatomical structure homeostasis | APP HSPB1 VEGFA | |
|  | microtubule-based movement | APP HTT HSPB1 | |
|  | positive regulation of positive chemotaxis | F2RL1 CXCL8 VEGFA | |
|  | regulation of positive chemotaxis | F2RL1 CXCL8 VEGFA | |
|  | vascular endothelial growth factor receptor signaling pathway | MAPK14 HSPB1 VEGFA | |
|  | cellular response to vascular endothelial growth factor stimulus | MAPK14 HSPB1 VEGFA | |
|  | enzyme-linked receptor protein signaling pathway | MAPK14 FOS GSK3B HSPB1 TP53 VEGFA | |
|  | regulation of angiogenesis | HSPB1 CXCL8 TNF VEGFA | |
|  | regulation of vasculature development | HSPB1 CXCL8 TNF VEGFA | |
|  | regulation of blood vessel endothelial cell migration | HSPB1 TNF VEGFA | |
|  | transmembrane receptor protein tyrosine kinase signaling pathway | MAPK14 GSK3B HSPB1 VEGFA | |
|  | positive regulation of angiogenesis | HSPB1 CXCL8 VEGFA | |
|  | positive regulation of vasculature development | HSPB1 CXCL8 VEGFA | |
|  | negative regulation of heart contraction | IL2 PIK3CG TNF | |
|  | negative regulation of blood circulation | IL2 PIK3CG TNF | |
|  | regulation of acute inflammatory response | IL4 PIK3CG TNF | |
|  | epithelial cell apoptotic process | BCL2L1 PIK3CG TNF | |
|  | regulation of lipid metabolic process | PIK3CG SNCA TNF | |
|  | cellular homeostasis | APP BAK1 CALCA EDN1 MCL1 NOS2 NPM1 | |
|  | aging | BAK1 CALCA EDN1 FOS | |
|  | cellular calcium ion homeostasis | APP BAK1 CALCA EDN1 | |
|  | calcium ion homeostasis | APP BAK1 CALCA EDN1 | |
|  | cellular divalent inorganic cation homeostasis | APP BAK1 CALCA EDN1 | |
|  | divalent inorganic cation homeostasis | APP BAK1 CALCA EDN1 | |
|  | regulation of transmembrane transporter activity | APP EDN1 HTT SNCA | |
|  | cellular metal ion homeostasis | APP BAK1 CALCA EDN1 | |
|  | regulation of transporter activity | APP EDN1 HTT SNCA | |
|  | regulation of supramolecular fiber organization | APP EDN1 F2RL1 SNCA | |
|  | cellular cation homeostasis | APP BAK1 CALCA EDN1 | |
|  | response to temperature stimulus | CALCA FOS HSPB1 | |
|  | positive regulation of supramolecular fiber organization | APP EDN1 F2RL1 | |
|  | metal ion homeostasis | APP BAK1 CALCA EDN1 | |
|  | cellular ion homeostasis | APP BAK1 CALCA EDN1 | |
|  | multi-organism reproductive process | CALCA EDN1 FOS | |
|  | regulation of cation channel activity | APP EDN1 HTT | |
|  | multi-multicellular organism process | CALCA EDN1 FOS | |
|  | response to ketone | BCL2L1 EDN1 FOS | |
|  | cation homeostasis | APP BAK1 CALCA EDN1 | |
|  | inorganic ion homeostasis | APP BAK1 CALCA EDN1 | |
|  | ion homeostasis | APP BAK1 CALCA EDN1 | |
|  | cellular chemical homeostasis | APP BAK1 CALCA EDN1 | |
|  | regulation of ion transmembrane transporter activity | APP EDN1 HTT | |
|  | growth | APP EDN1 TP53 | |
|  | developmental growth | APP EDN1 TP53 | |
|  | viral process | BCL2L1 CDK1 DPP4 GSK3B TP53 | |
|  | biological process involved in interaction with host | BCL2L1 CDK1 DPP4 | |
|  | biological process involved in symbiotic interaction | BCL2L1 CDK1 DPP4 | |
|  | positive regulation of cyclase activity | CALCA MAPK14 NOS2 | |
|  | regulation of cyclase activity | CALCA MAPK14 NOS2 | |
|  | regulation of phosphoprotein phosphatase activity | GSK3B HTT TNF | |
|  | regulation of organelle assembly | GSK3B HTT NPM1 TNF | |
|  | positive regulation of organelle assembly | GSK3B HTT TNF | |
|  | regulation of calcium-mediated signaling | GSK3B HTT TNF | |
|  | regulation of phosphatase activity | GSK3B HTT TNF | |
|  | regulation of protein dephosphorylation | GSK3B HTT TNF | |
|  | positive regulation of plasma membrane bounded cell projection assembly | F2RL1 GSK3B HTT | |
|  | regulation of dephosphorylation | GSK3B HTT TNF | |
|  | positive regulation of autophagy | GSK3B HTT IL4 | |
|  | positive regulation of cell projection organization | F2RL1 GSK3B HTT VEGFA | |
|  | regulation of macroautophagy | HTT IL4 TP53 | |
|  | regulation of plasma membrane bounded cell projection assembly | F2RL1 GSK3B HTT | |
|  | regulation of cell projection assembly | F2RL1 GSK3B HTT | |
|  | regulation of plasma membrane bounded cell projection organization | F2RL1 GSK3B HTT VEGFA | |
|  | regulation of cell projection organization | F2RL1 GSK3B HTT VEGFA | |
|  | regulation of tissue remodeling | CALCA IL2 TP53 | |
|  | positive regulation of cell projection organization |  | |
|  | regulation of macroautophagy | APP BAK1 CCND3 CD40LG MAPK14 DPP4 EDN1 F2RL1 HSPB1 IL2 IL4 IL6R CXCL8 PIK3CG SNCA TNF TP53 | |
|  | regulation of plasma membrane bounded cell projection assembly | APP BAK1 CCND3 CD40LG DPP4 EDN1 F2RL1 IL2 IL4 IL6R CXCL8 PIK3CG SNCA TNF TP53 | |
|  | regulation of cell projection assembly | APP CD40LG F2RL1 IL2 IL4 IL6R PIK3CG TP53 | |
|  | regulation of plasma membrane bounded | BAK1 CCND3 CD40LG DPP4 F2RL1 IL2 IL4 IL6R PIK3CG TP53 | |
|  | regulation of cell projection organization | BAK1 BCL2L1 CCND3 CD40LG CDK1 F2RL1 FOS IL2 PIK3CG TNF TP53 | |
|  | regulation of tissue remodeling | BCL2L1 CCND3 CD40LG F2RL1 IL2 PIK3CG TP53 | |
| CC | Bcl-2 family protein complex | BAK1 BCL2L1 MCL1 | |
|  | mitochondrial outer membrane | BAK1 BCL2L1 MCL1 | |
|  | organelle outer membrane | BAK1 BCL2L1 MCL1 | |
|  | outer membrane | BAK1 BCL2L1 MCL1 | |
|  | cyclin-dependent protein kinase holoenzyme complex | CCNA2 CCND3 CDK1 CDK2 | |
|  | serine/threonine protein kinase complex | CCNA2 CCND3 CDK1 CDK2 | |
|  | protein kinase complex | CCNA2 CCND3 CDK1 CDK2 | |
|  | transferase complex, transferring phosphorus-containing groups | CCNA2 CCND3 CDK1 CDK2 PIK3CG | |
|  | centrosome | BCL2L1 CDK1 CDK2 GSK3B NPM1 TP53 | |
|  | germ cell nucleus | CDK2 TOP1 TP53 | |
|  | axon | APP CALCA GSK3B HTT HSPB1 SNCA | |
|  | neuronal cell body | APP CALCA SNCA TNF TOP1 | |
|  | presynapse | APP BCL2L1 CALCA HTT SNCA | |
|  | cell body | APP CALCA SNCA TNF TOP1 | |
|  | transport vesicle | APP BCL2L1 EDN1 SNCA | |
|  | synaptic vesicle | APP BCL2L1 SNCA | |
|  | exocytic vesicle | APP BCL2L1 SNCA | |
|  | distal axon | APP CALCA SNCA | |
|  | postsynapse | APP GSK3B HTT SNCA | |
|  | perinuclear region of cytoplasm | APP HTT NOS2 SNCA | |
|  | early endosome | APP F2RL1 HTT | |
|  | spindle | APP CDK1 MAPK14 HSPB1 NPM1 | |
|  | focal adhesion | DPP4 HSPB1 NPM1 | |
|  | cell-substrate junction | DPP4 HSPB1 NPM1 | |
|  | platelet alpha granule | APP SNCA VEGFA | |
|  | secretory granule lumen | APP MAPK14 VEGFA | |
|  | cytoplasmic vesicle lumen | APP MAPK14 VEGFA | |
|  | vesicle lumen | APP MAPK14 VEGFA | |
|  | protein-DNA complex | FOS NPM1 TOP1 | |
|  | membrane raft | APP DPP4 TNF | |
|  | membrane microdomain | APP DPP4 TNF | |
|  | apical part of cell | APP DPP4 IL6R | |
| MF | signaling receptor activator activity | APP CALCA CD40LG DPP4 EDN1 IL2 IL4 CXCL8 TNF VEGFA | |
|  | signaling receptor regulator activity | APP CALCA CD40LG DPP4 EDN1 IL2 IL4 CXCL8 TNF VEGFA | |
|  | receptor ligand activity | CALCA CD40LG DPP4 EDN1 IL2 IL4 CXCL8 TNF VEGFA | |
|  | cytokine activity | CD40LG EDN1 IL2 IL4 CXCL8 TNF VEGFA | |
|  | cytokine receptor binding | CD40LG IL2 IL4 IL6R CXCL8 TNF VEGFA | |
|  | growth factor receptor binding | APP IL2 IL4 IL6R VEGFA | |
|  | G protein-coupled receptor binding | CALCA EDN1 IL2 CXCL8 | |
|  | growth factor activity | IL2 IL4 VEGFA | |
|  | heparin binding | APP CXCL8 VEGFA | |
|  | glycosaminoglycan binding | APP CXCL8 VEGFA | |
|  | sulfur compound binding | APP CXCL8 VEGFA | |
|  | kinase regulator activity | CCNA2 CCND3 CD40LG HSPB1 IL2 NPM1 SNCA | |
|  | protein kinase regulator activity | CCNA2 CCND3 CD40LG HSPB1 NPM1 SNCA | |
|  | protein kinase inhibitor activity | HSPB1 NPM1 SNCA | |
|  | kinase inhibitor activity | HSPB1 NPM1 SNCA | |
|  | enzyme inhibitor activity | APP HSPB1 NPM1 SNCA | |
|  | protein homodimerization activity | BAK1 BCL2L1 DPP4 HSPB1 IL6R MCL1 NOS2 NPM1 VEGFA | |
|  | kinase binding | BCL2L1 CCNA2 CCND3 MAPK14 GSK3B HTT HSPB1 NPM1 TP53 | |
|  | protein kinase binding | BCL2L1 CCNA2 CCND3 MAPK14 GSK3B HSPB1 NPM1 TP53 | |
|  | RNA polymerase II-specific DNA-binding transcription factor binding | MAPK14 FOS GSK3B HSPB1 NPM1 TP53 | |
|  | DNA-binding transcription factor binding | MAPK14 FOS GSK3B HSPB1 NPM1 TP53 | |
|  | core promoter sequence-specific DNA binding | FOS NPM1 TP53 | |
|  | transcription factor binding | MAPK14 FOS GSK3B HSPB1 NPM1 TP53 | |
|  | chromatin binding | CDK1 FOS NPM1 TOP1 TP53 | |
|  | BH domain binding | BAK1 BCL2L1 MCL1 | |
|  | protein domain specific binding | APP BAK1 BCL2L1 CCNA2 CDK2 MCL1 TOP1 TP53 | |
|  | protein heterodimerization activity | BAK1 BCL2L1 MCL1 TP53 | |
|  | protein serine/threonine kinase activity | CCND3 CDK1 CDK2 MAPK14 GSK3B PIK3CG TOP1 | |
|  | protein kinase activity | CCND3 CDK1 CDK2 MAPK14 GSK3B PIK3CG TOP1 | |
|  | cyclin-dependent protein serine/threonine kinase activity | CCND3 CDK1 CDK2 | |
|  | cyclin-dependent protein kinase activity | CCND3 CDK1 CDK2 | |
|  | phosphotransferase activity, alcohol group as acceptor | CCND3 CDK1 CDK2 MAPK14 GSK3B PIK3CG TOP1 | |
|  | kinase activity | CCND3 CDK1 CDK2 MAPK14 GSK3B PIK3CG TOP1 | |
|  | protein serine kinase activity | CDK1 CDK2 MAPK14 GSK3B PIK3CG | |
|  | protease binding | DPP4 F2RL1 GSK3B TNF TP53 | |
|  | p53 binding | GSK3B HTT TP53 | |
|  | heat shock protein binding | BAK1 CDK1 HTT SNCA | |

Supplementary table 8. Area Under the Curve for TP53

Test Result Variable(s) Depression

| Area | Std Error^a^ | Asymptotic sig^b^ | Asymptotic 95% confidence interval | |
| --- | --- | --- | --- | --- |
|  |  |  | Lower Bound | Upper Bound |
| .889 | .027 | .000 | .835 | .942 |

1. under the nonparametric assumption
2. Null hypothesis; true area=0.5

Supplementary table 9. Area Under the Curve for TNF

Test Result Variable(s) Depression

| Area | Std Error^a^ | Asymptotic sig^b^ | Asymptotic 95% confidence interval | |
| --- | --- | --- | --- | --- |
|  |  |  | Lower Bound | Upper Bound |
| .668 | .084 | .068 | .503 | .833 |

1. under the nonparametric assumption
2. Null hypothesis; true area=0.5
